# Cooperative Lewis Acid/Metal Dual Catalysis for the Selective *ortho*-Alkylation of Phenols

**DOI:** 10.1021/acssuschemeng.5c04668

**Published:** 2025-07-25

**Authors:** Benedetta Di Erasmo, Edoardo Bazzica, Giulia Brufani, Chao-Jun Li, Luigi Vaccaro

**Affiliations:** † Laboratory of Green S.O.C. − Dipartimento di Chimica, Biologia e Biotecnologie, 9309 Università degli Studi di Perugia, Via Elce di Sotto 8, 06123 Perugia, Italy; ‡ Department of Chemistry and FRQNT Centre for Green Chemistry and Catalysis, McGill University, 801 Sherbrooke Street West, Montreal, Quebec H3A 0B8, Canada

**Keywords:** phenols, flow chemistry, heterogeneous catalysis, *ortho*-alkylation, primary alcohols, C−H functionalization

## Abstract

Alkylated phenols hold significant importance in industrial chemistry, as many pharmaceuticals and polymer additives are based on these scaffolds. However, synthesizing these products in a sustainable way is still challenging because stoichiometric amounts of Lewis acids to promote Friedel–Crafts reactions on benzene and phenol derivatives are often employed, together with drastic and unsafe reaction conditions. In this paper, we present a regioselective process to produce *ortho*-alkylated phenols, with high atom- and step-economy that minimizes the formation of waste associated with it. The key role is pursued by the dual catalytic system palladium on carbon (Pd/C) and scandium trifluoromethanesulfonate (Sc­(OTf)_3_), which together promote the partial hydrogenation of phenol to cyclohexenone, while the alcohol is oxidized to aldehyde. The so-formed partners couple in an aldol condensation promoted by Sc­(OTf)_3_, which is then rearomatized in the air atmosphere. Amylmetacresol (antiseptic) was successfully obtained with this protocol in a one-step synthesis for the first time. Moreover, this process permits the recovery and reuse of the Pd/C catalyst for up to 5 runs. This paved the way for the development of a gram scale-up flow protocol associated with this strategy in an innovative tube-in-tube setup.

## Graphical abstract

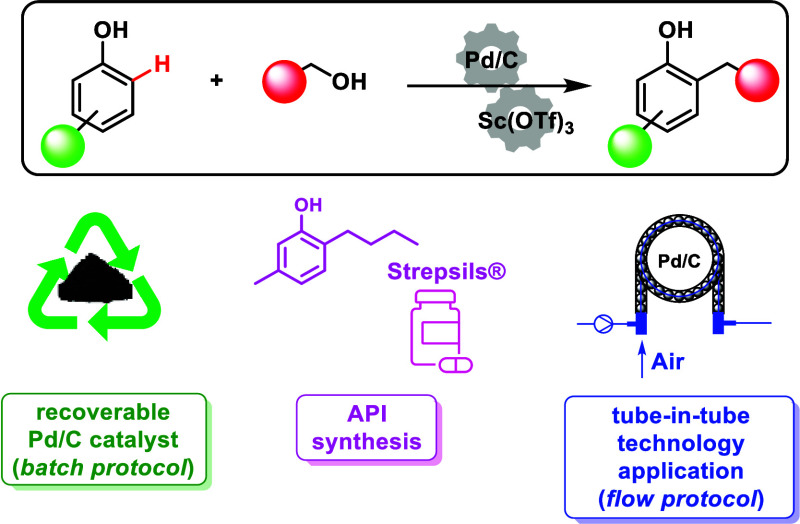

## Introduction

Functionalized phenols are important scaffolds used in different areas, from pharmaceuticals to the agricultural industry, and from polymeric materials to biofuel production.^1–9^ Even if most phenols are formed via the industrial Hock process,^10^ recently, the potential to obtain phenolic units from lignin has been enlightened. Indeed, structurally diverse phenols, typically featuring methoxy or methyl groups (i.e., guaiacols), can be derived from this natural and renewable raw material through depolymerization processes.^11–15^ Among the plethora of value-added phenolic derivatives, alkylated phenols have attracted growing attention due to their commercial potential as nonionic surfactants, phenolic resins, polymers, and polymer additives. Furthermore, the alkylated phenolic structural motif is commonly found in the scaffolds of APIs such as amylmetacresol, agrochemicals such as Dinoterb, Dinoseb, and DNOC, and in the scaffold of catecholamines (Figure 1).^8,16–18^

**Figure 1:**
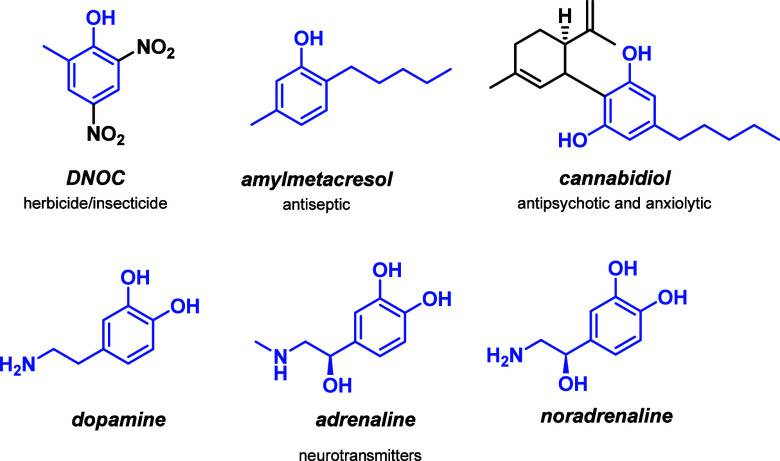
Value-added alkylated phenols.

Alkylated phenols are generally obtained from electrophilic aromatic substitutions (SEAr). Indeed, the presence of the hydroxyl group activates phenols for the SEAr.^19–21^ However, controlling the selectivity is truly challenging: the steric factor promotes the attack at the *para*-position while the statistical factor promotes the formation of the *ortho*-product. For this reason, the insertion of a directing group in the phenol is often used as a strategy to promote a more selective process.^22,23^ However, this strategy does not promote a good step economy, generating large amounts of unwanted waste. Nowadays, there are examples of C–H activation, but they often use directing groups, toxic solvents or reagents, and expensive catalysts.^24–31^

Primary alcohols are biomass-derived compounds,^32–35^ inexpensive, and abundant and highly attractive for green synthetic chemistry utilities.^36–38^ Allylic, benzylic and tertiary alcohols are used as reaction partners in the Friedel–Crafts alkylation of phenols using excess of Bro̷nsted acids or stoichiometric amounts of Lewis acids without being able to completely control the regio- and stereochemistry (Figure 2a).^39–44^ An example of this is provided by Upadhyayula and co-workers. They pursued a zeolite-mediated gas-phase alkylation of *m*-cresol with iso-propanol. The cresol reacts with 92% conversion, but the selectivity toward thymol is 71% since other regio-isomers are also formed.^45,46^ In 2022, Kumar and co-workers developed an *ortho*-alkylation of phenols catalyzed by a pincer–ruthenium complex that, in the presence of molecular oxygen, leads to both cross etherification and *ortho*-alkylation. They are selective toward the *ortho*-alkylation when they use β-naphthol with 1-phenylethanol derivatives and phenol with diphenylmethanol.^47^ Other strategies have been explored to access alkylated phenols with alcohols as coupling agents. Yi and co-workers used a homogeneous Ru catalyst to promote a dehydrative C–H alkylation with secondary and tertiary alcohols in the first step of the formation of benzofurans.^48^ Very recently, Du et al. disclosed a TiO_2_-catalyzed C–H alkylation where the formation of a Ti=C-bond with the α-carbon of the alkyl group at oxygen vacancies is the key step of the process.^49^

**Figure 2:**
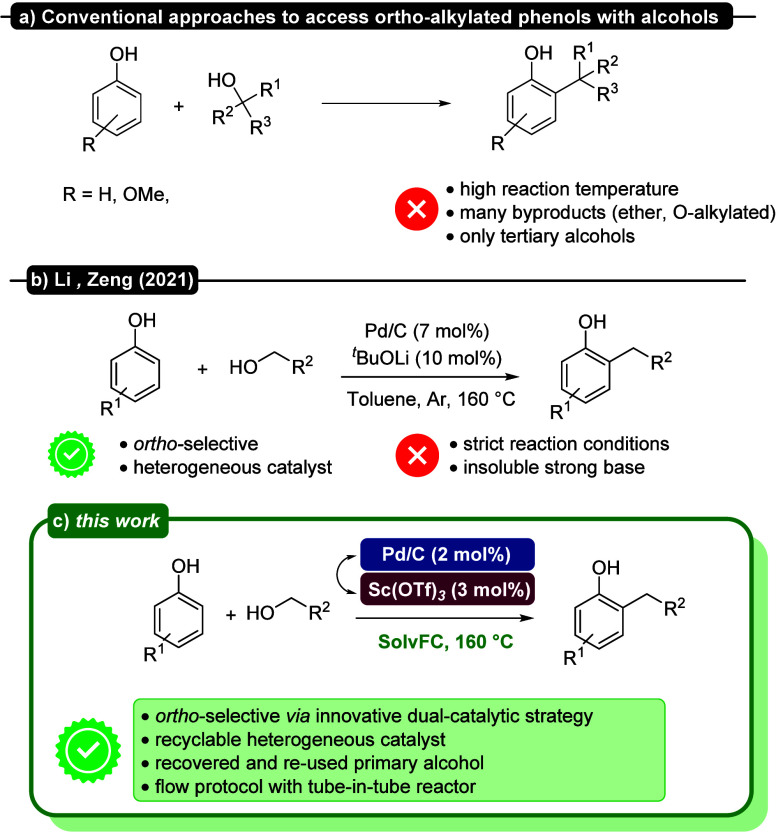
Summary of the possible pathways for *ortho*-alkylation of phenols with alcohols.

In 2021, a completely different chemistry was employed to obtain *ortho*-alkylated phenols by Zeng and co-workers (Figure 2b).^50,51^ For the first time, they proposed a hydrogenation/dehydrogenation pathway: the primary alcohol is converted to the corresponding aldehyde, forming HPdH species that can transfer hydrides to the phenol to be selectively reduced to cyclohexanone. Then, an aldol reaction occurs between the readily formed ketone and the aldehyde, giving the aldol adduct that is rearomatized by Pd/C.^50^

Despite the innovation brought by this strategy, there are some issues to address, such as the ultra-anhydrous reaction conditions, together with the use of the insoluble *t*-BuOLi, which make it difficult to scale up and smoothly implement the process in industry. The ability of Lewis acids to promote aldol condensations in mild reaction conditions^52–55^ combined with the proven ability of the Pd–Lewis acid systems to promote the selective partial reduction of phenol to cyclohexanone^56,57^ suggests that the use of these additives could be beneficial for the reaction. Furthermore, the use of a soluble additive allows the protocol to serve as an effective approach for continuous-flow applications. Herein, we present a cooperative dual-catalytic system where heterogeneous Pd/C works together with Lewis acid additives to promote a tandem dearomatization/aldol condensation/rearomatization strategy. Briefly, phenol undergoes partial hydrogenation enabled by the hydrides provided by the oxidation of the alcohol to the aldehyde, and then the Lewis additive promotes the aldol condensation followed by the rearomatization of the aldol adduct (Figure 2c). The reaction conditions were extensively optimized in solvent-free conditions (SolvFC), followed by the application of this strategy to 20 substrates (7 of them potentially derived from lignin), achieving remarkable yields. We also successfully obtained amylmetacresol in a one-step synthesis for the first time. After investigating the reaction pathway through mechanistic controls, we recycled the catalyst up to 5 runs in the batch process. The ability to recover and recycle the catalyst permitted the implementation of a flow protocol using an innovative tube-in-tube strategy.^58–60^

## Results and Discussion

We carried out optimization steps under SolvFC since this strategy implies a closer proximity between reagents that could lead to better reactivity. Furthermore, the SolvFC protocol can, in accordance with the principles of Green Chemistry, minimize the waste associated with the process. First, a screening of different Lewis acid additives to promote the aldol condensation, including some relatively strong acids such as metal triflates and weaker acids such as metal bromides, metal chlorides, and metal nitrates, was pursued (Table S1). The acid that produced the best results was scandium­(III) triflate (Sc­(OTf)_3_). The temperature that yielded the best results in SolvFC was 160 °C.

Table 1 shows various tests carried out to optimize the relative quantities of the catalyst and Lewis acid for the model reaction between 4-methoxyphenol and 1-hexanol. First, the Pd/C amount was changed, keeping the Sc­(OTf)_3_ amount constant (entries 1–4), and then the quantity of Sc­(OTf)_3_ was changed, keeping the Pd/C amount steady (entries 5–7). In the second case, with the decrease in the Lewis acid amount, conversion also decreased, defining the importance of the Lewis acid amount in initiating the reaction. Once the optimal ratio between the two quantities was found (0.7), they were changed separately (entries 8–10). The best conditions resulted in 2 mol % of Pd/C and 3 mol % of Sc­(OTf)_3_ to minimize the formation of the bis-alkylated side product. Finally, the reaction time (entries 10–12) was optimized to obtain the greatest conversion possible without loss of selectivity.

**Table 1:**
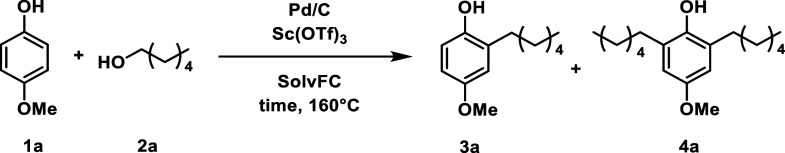
Optimization of Reaction Conditions of Pd/C and Sc­(OTf)_3_ ^a^

entry	time (h)	Pd/C (mol %)	Sc(OTf)_3_ (mol %)	Pd/C: Sc(OTf)_3_	**1a** (%)^b^	**3a** (%)^b^	**4a** (%)^b^
1	16	0	10	0	90	8	2
2	16	2	10	0.2	0	62	38
3	16	5	10	0.5	0	13	87
4	16	7	10	0.7	25	40	38
5	16	7	8	0.9	0	59	41
6	16	7	3	2.3	55	28	17
7	16	7	1	7	57	12	31
8	16	7	10	0.7	4	40	56
9	16	5	7	0.7	5	69	26
10	16	2	3	0.7	40	60	0
11	20	2	3	0.7	7	93	0
12	24	2	3	0.7	4	77	19

a Reaction conditions: **1a** (0.4 mmol), **2a** (20 equiv), Pd/C, Sc­(OTf)_3_, 160 °C, varied times, 12 mL vial.

b Determined by GLC analyses.

With the catalyst loadings and time optimized, the optimal amount of 1-hexanol was examined (Table 2). A low quantity of 1-hexanol (entry 1) leads to a more concentrated solution of the catalyst and Lewis acid and so to an excess of reactivity (only side product is formed), while beyond 20 equiv of 1-hexanol (Table 2, entry 4), Pd/C and Sc­(OTf)_3_ are more diluted, leading to a lower conversion. Therefore, optimized reaction conditions require the use of 20 equiv of 1-hexanol.

**Table 2:**
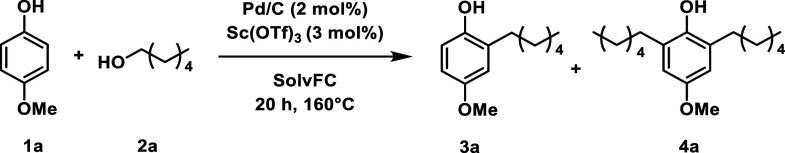
Optimization of Reaction Conditions of 1-Hexanol^a^

entry	1-hexanol amount (equiv)	**1a** **(%)** ^b^	**3a** **(%)** ^b^	**4a** **(%)** ^b^
1	5	0	0	100
2	10	20	70	10
3	20	7	93	0
4	30	24	76	0

a Reaction conditions: **1a** (0.4 mmol), **2a**, Pd/C (2 mol %), Sc­(OTf)_3_ (3 mol %), 160 °C, 20 h, 12 mL vial.

b Determined by GLC analyses.

To increase the efficiency of our procedure, we recovered 1-hexanol (81% of the initial amount) and analyzed it via ^1^H NMR spectroscopy, confirming its purity. Reusing it, very similar results were obtained compared to the fresh one (80 vs 81% of product **3a** yield).

Afterward, the applicability of this optimized strategy was explored (Table 3), first varying the phenol partner while keeping 1-hexanol as the primary alcohol. For each substrate, the optimal reaction time that led to the highest conversion to the *ortho*-alkylated product was determined by monitoring the reaction over time using GC analysis. At the beginning of our study, we compared the position effect on the starting material. We found that *para*-methoxyphenol (**1a**) gives a better yield (81%) than *meta*-methoxyphenol (**1b**, 60%) because the **1b** bis-alkylated side product is formed, while *ortho*-methoxyphenol (guaiacol) resulted in no conversion even after long reaction times (24h). The reason can be attributed to the fact that the methoxy group has a deactivated effect on the groups that are in the meta position, leading to 0% conversion. On investigating the substitution effects, we observed traces of the *para*-adduct together with product **3c** because the substrate could also be activated for the Friedel–Crafts reaction. However, by shortening the reaction time to 1.5 h, we could achieve a very good yield (64%). Product **3d** is obtained in 84% yield thanks to the 3 electron-donating groups on the starting phenol. Comparing product **3f** with product **3g,** we can notice that the latter needs a much longer reaction time to produce the same yield as the monomethylated one. This is possibly due to the steric hindrance of the methyl group at the *meta* position. Different hindered groups in the *para* position, ethyl, isopropyl, and *tert*-butyl (**3h**–**i**) give the same yields (up to 63%). The EWG-substituted 4-fluorophenol (**1k**) gives an unsatisfactory yield of **3k** due to dehalogenation and the deactivating effect of the electron-withdrawing group.

**Table 3:**
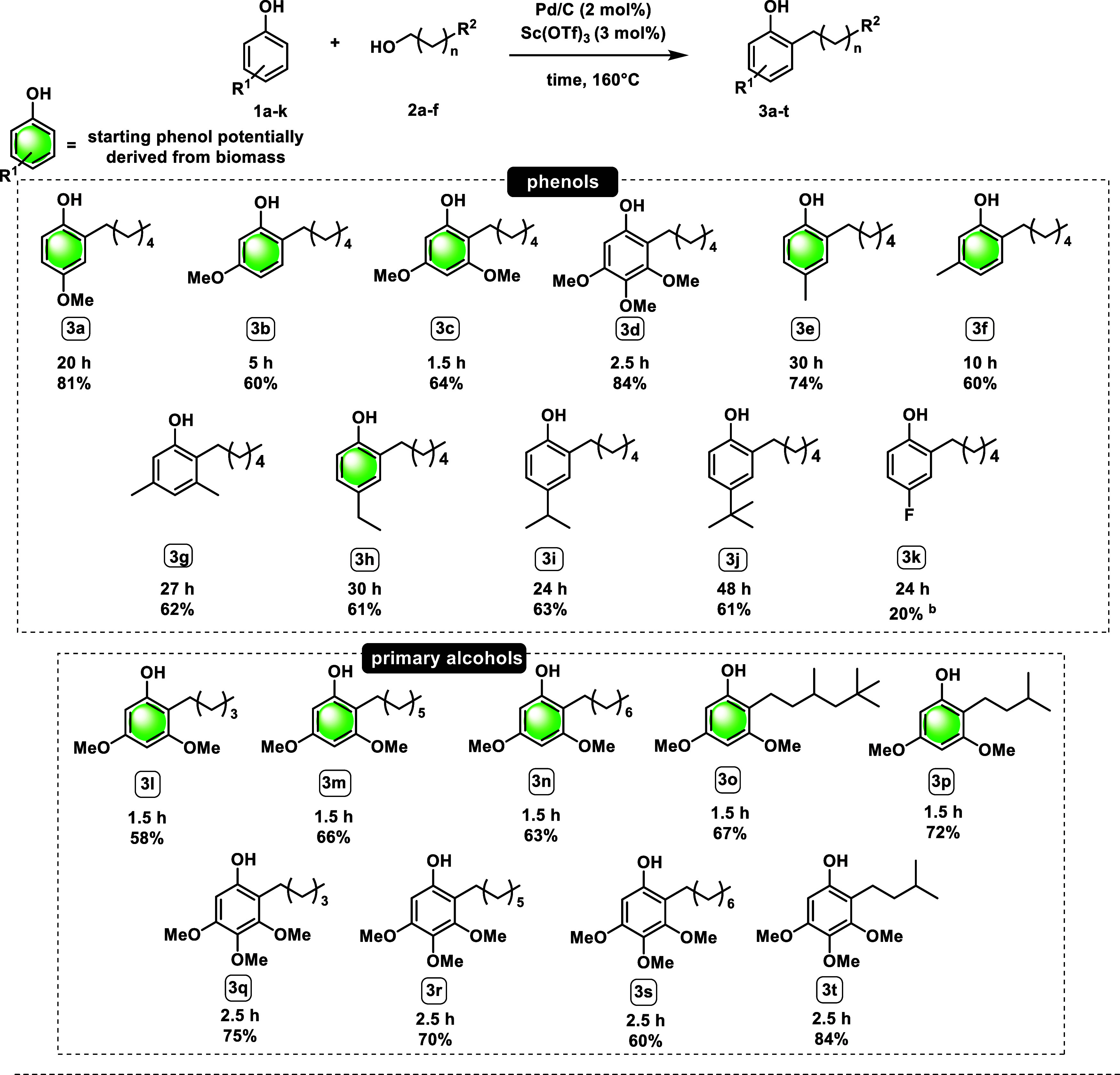
*Ortho*-Alkylation of Different Phenols with Primary Alcohols^a^

a Reaction conditions: **1a**–**l** (0.4 mmol), **2a**–**f** (20 equiv), Pd/C (2 mol %), Sc­(OTf)_3_ (3 mol %), SolvFC, 160 °C, varied times, 12 mL vial. Isolated yields are shown.

b NMR yield.

**Scheme 1:**
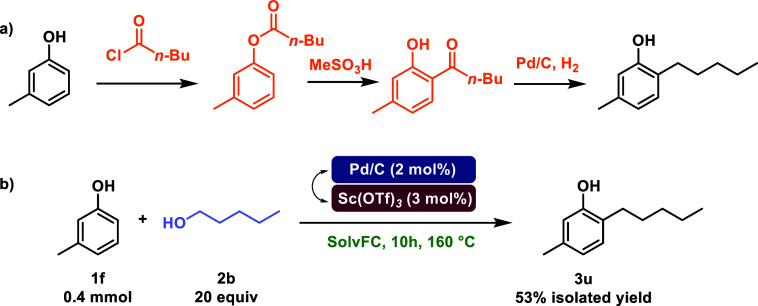
(a) Current Synthetic Route for the Synthesis of Amyl-m-cresol; (b) Amyl-m-cresol Synthesis via Our Cooperative Dual Catalytic System

**Scheme 2:**
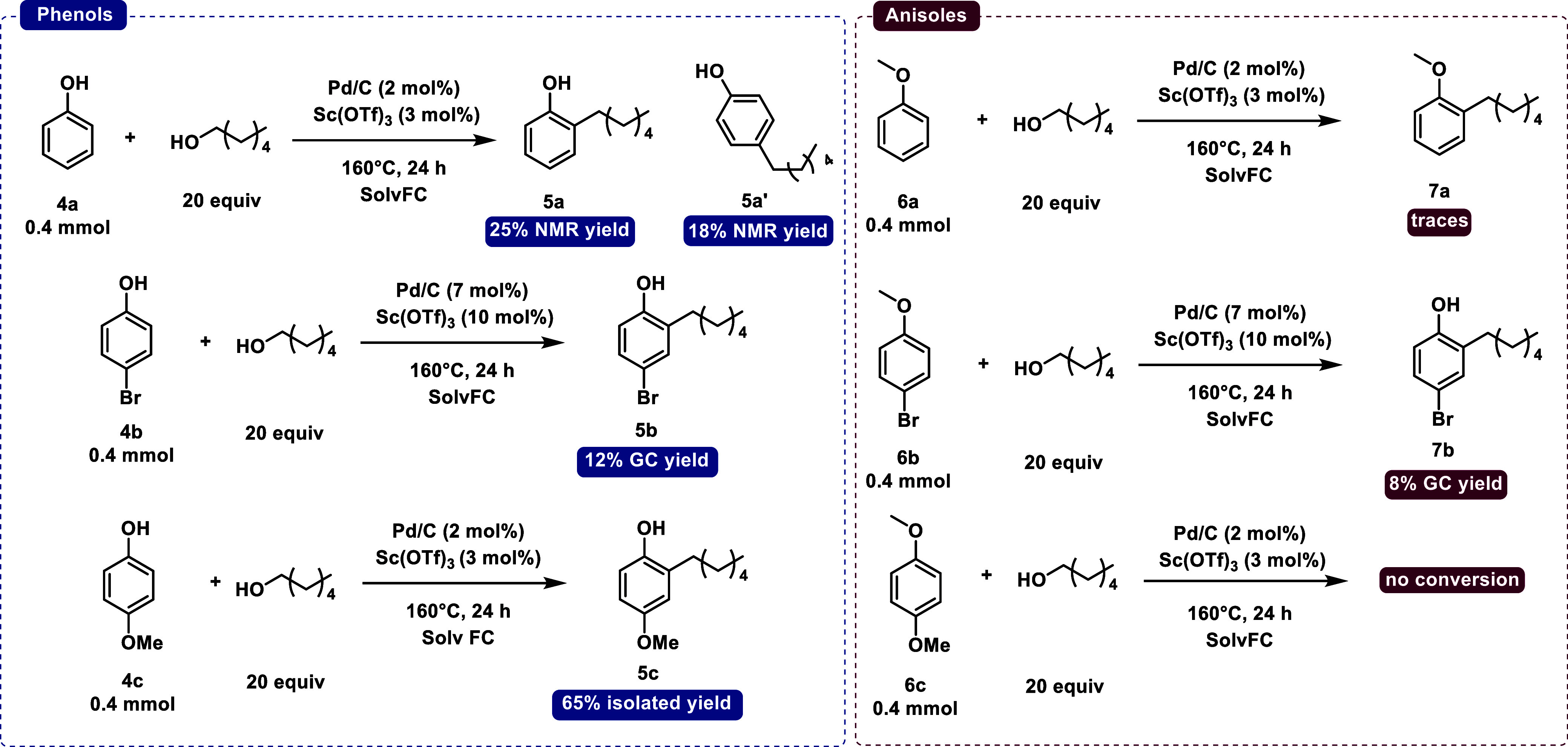
Control Tests Comparing Phenols with Anisoles^a^

a Nitrobenzene and 1,3,5-trimethoxybenzene have been used as internal standards to calculate NMR and GC yields, respectively. The remaining percentage in all of the reactions corresponds to the starting material, except for the reaction of **4c**, which gives complete conversion with side product 2,6-dihexyl-4-methoxyphenol formation in 30%.

By varying the length of the alcohol alkyl chain (**3l**–**n**), very good results were obtained, with no great differences observed between them. Also, using branched alcohols (**3o** and **3p**) allows the formation of *ortho*-alkylated phenols in excellent yields, up to 75%. The same satisfactory trend is shown in the products **3q**–**3t**. To address the limitations of the method, we tried methanol and ethanol as coupling partners: these gave no conversion because of the intrinsic nature of the reaction mechanism and because of their low boiling points.

**Scheme 3:**
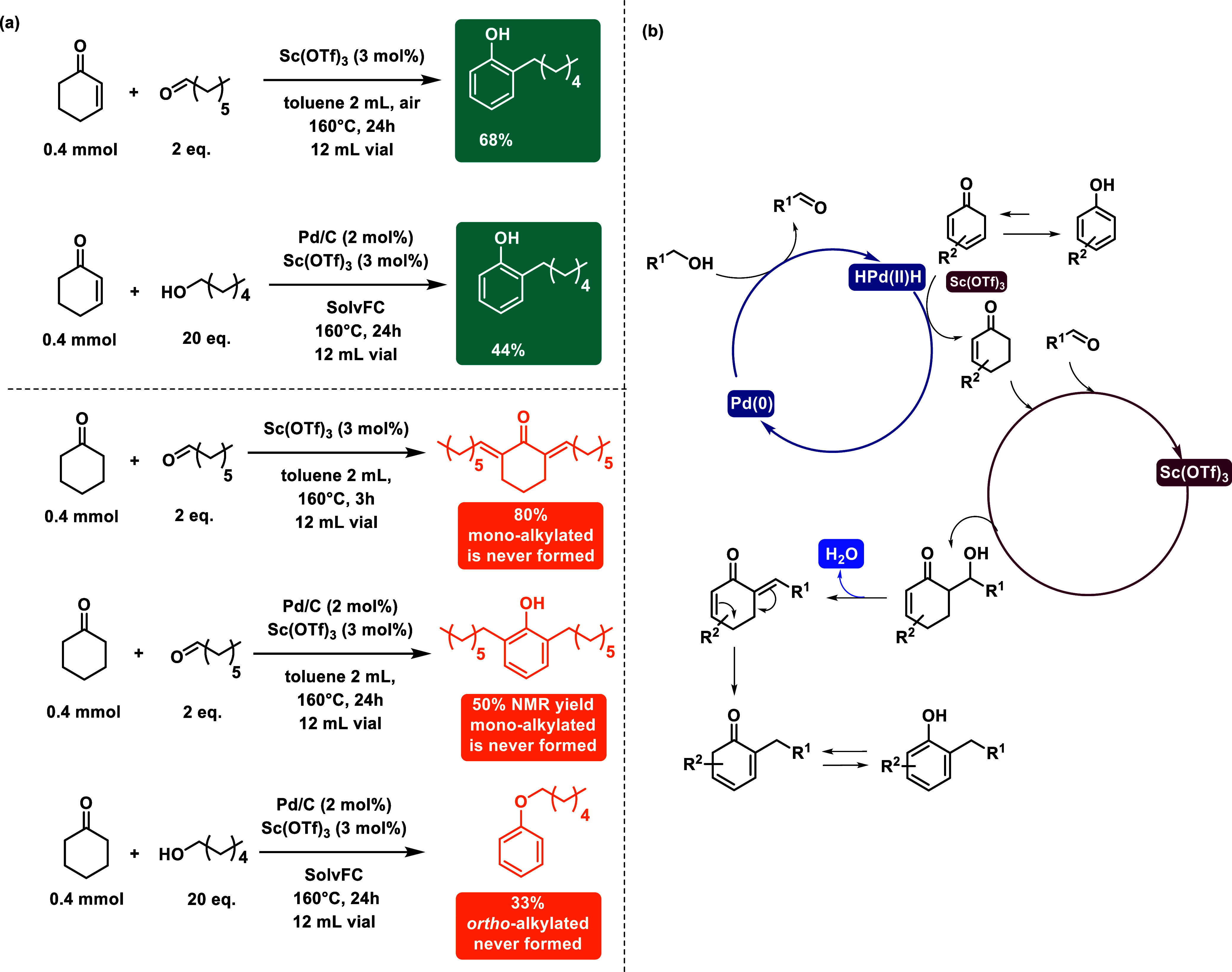
(a) Mechanistic Controls with Cyclohexenone and Cyclohexanone; (b) Proposed Cooperative Catalytic Mechanism for the *ortho*-Alkylation of Phenols with Primary Alcohols^a^

a All yields are GC yields calculated using 1,3,5-trimethoxybenzene as the internal standard. The NMR yield is calculated using nitrobenzene as the internal standard.

Among alkylated phenols of pharmaceutical interest, 2-pentyl-5-methylphenol, also known as amyl-*m*-cresol, is a useful antiseptic and germicide. It is employed in combination with 2,4-dichlorobenzyl alcohol in Strepsils, which serves for sore throats and hoarseness treatment after tracheal intubation.^61^ A common way to synthesize amyl-*m*-cresol is from *m*-cresol and valeryl chloride, followed by the in situ rearrangement of the 3-toluoyl valerate in the presence of an oxoacid to obtain valeryl m-cresol. The reduction of this species yields the desired alkylated phenol (Scheme 1a).^62^ However, this strategy involves the use of 3 steps, with product purification for two of them. With our protocol, instead, we were able to achieve amyl-*m*-cresol in just one step, minimizing the step and atom economy together with a substantial minimization of waste associated with the process (Scheme 1b).

To investigate the possible reaction pathway, we first monitored the reaction over time using GC analysis (Table S3): we observed the formation of the *ortho-*alkylated product **3a,** without any intermediate related to it even at short reaction times. This can possibly mean that all of the intermediates involved in the reaction are transient. To investigate whether the reaction could proceed via a Friedel–Crafts pathway or not we run reactions in the optimized conditions with 1-hexanol (Scheme 2) and different functionalized phenols (phenol **4a**, 4-bromophenol **4** **b**, and 4-methoxyphenol **4c**) or the corresponding anisoles (anisole **6a**, 4-bromoanisole **6b**, and 4-methoxyanisole **6c**). If the conversion significantly decreases when anisoles (good substrates for aromatic electrophilic substitution) are used, then it means that the Friedel–Crafts pathway is not the most favorable one. Phenol **4a** to *ortho*-alkylated phenol **5a** gave 25% NMR yield, while for anisole **6a,** we obtained traces of the alkylated anisole **7a**, with the starting anisole remaining unreacted. 4-Bromophenol **4b** is a deactivated substrate that showed low conversion to the alkylated bromophenol **5b** (12%); for the corresponding anisole we obtained quite similar results, with the removal of the methoxy group to form the hydroxyl one (8%), along with the unreacted starting material. The reaction with 4-methoxyphenol **4c** and 1-hexanol gave 65% isolated yield of 2-hexyl-4-methoxyphenol **5c**: 4-methoxyanisole **6c** instead showed no conversion at all under our conditions. The significant drop in conversion when anisoles are used as substrates with respect to phenols led to the conclusion that the Friedel–Crafts mechanism is not the principal mechanism.

Mechanistic controls (Scheme 3a) have been investigated drawing on previous studies.^50,56,57,63^ We first tried to understand whether it would be possible for phenol to undergo complete hydrogenation to cyclohexanone or whether the reaction could pass through cyclohexenone. We pursued reactions with cyclohexenone (Scheme 3a, top)/cyclohexanone (Scheme 3a, bottom) and 1-heptanal/1-hexanol. Cyclohexanone controls with both 1-hexanol and 1-heptanal resulted in bis-alkylated and O-alkylated formation, while both cyclohexenone reactions yielded 2-hexylphenol. This suggested that cyclohexenone can be the key intermediate in the reaction, while cyclohexanone is mainly responsible for the formation of the bis-alkylated side product. Furthermore, the control between cyclohexenone and 1-heptanal with only Sc­(OTf)_3_ provided 68% of the product, indicating that after the aldol condensation, the proposed mechanism could involve an oxidative rearomatization of the aldol adduct without interference of the Pd catalyst. In Scheme S2, we observed that, even without Pd, the aldol adduct gives the desired product in an air atmosphere, while under Ar, the reaction stops. This experimental evidence, together with the previous cited literature,^50,56,57,63^ suggests that the mechanism could undergo a cooperative bimetallic catalytic pathway between Pd and Sc (Scheme 3b). Pd possibly mediates the formation of the transient aldehyde from primary alcohols where dihydride Pd species are formed and, together with the Lewis acid, promote the reduction of the phenol substrate to the corresponding cyclohexenone. After that, Sc­(OTf)_3_ is involved in the aldol condensation to produce the aldol product that undergoes an oxidative rearomatization with the elimination of one water molecule.

Furthermore, we investigated whether the reaction passes through a heterogeneous catalysis or undergoes a release-and-catch mechanism.^64^ The difference between the two is that for the first one, the metal nanoparticles remain on the support and the reaction happens on the active sites, while for the other one, the metal nanoparticles go out from the catalytic support into the solution and then, when the reaction is finished, the nanoparticles return to the sites.^64^ To understand which one could be our case, we performed two tests: a hot-filtration test at half the reaction time and a homogeneous test. For the first one, we measured the leaching value: we obtained 16.4 ppm at half reaction time vs 10.3 ppm after reaction completion (Figure S1). This means that during the reaction, there are more nanoparticles dispersed than at the end. For the second test, we first run the reaction without adding any phenol substrate, with only Pd/C, Sc­(OTf)_3_, and 1-hexanol; then, we filtered the catalyst on cotton, added 4-methoxyphenol **1a** in the supernatant, and then heated it again to run the reaction. Whether the starting material **1a** is converted to the product could provide a clue regarding the role of Pd nanoparticles in the mechanism. This test gave 66% conversion to the desired product **3a**, which is satisfactory compared with the heterogeneous result (93%). These two tests suggest that the reaction may pass through a so-called release-and-catch mechanism.

In order to evaluate the catalyst recovery with the main goal of implementing a flow protocol, we conducted recycle tests of Pd/C for the reaction between 4-methoxyphenol **1a** and 1-hexanol **2a** (Scheme 4 and Table S4). Between one run and the other, the catalyst was recovered using a Hirsh funnel and washed with EtOAc. For each run, GC conversion to **3a**, leaching tests, and the Pd loss percentage were calculated. We observed reproducible conversions ranging from 80% to 92% across all runs, with only 1.66% total Pd loss. The slight decrease in conversion observed in run IV cannot be attributed to Pd loss, as a conversion of 95% was achieved in run V, confirming the recyclability of the catalyst.

**Scheme 4:**
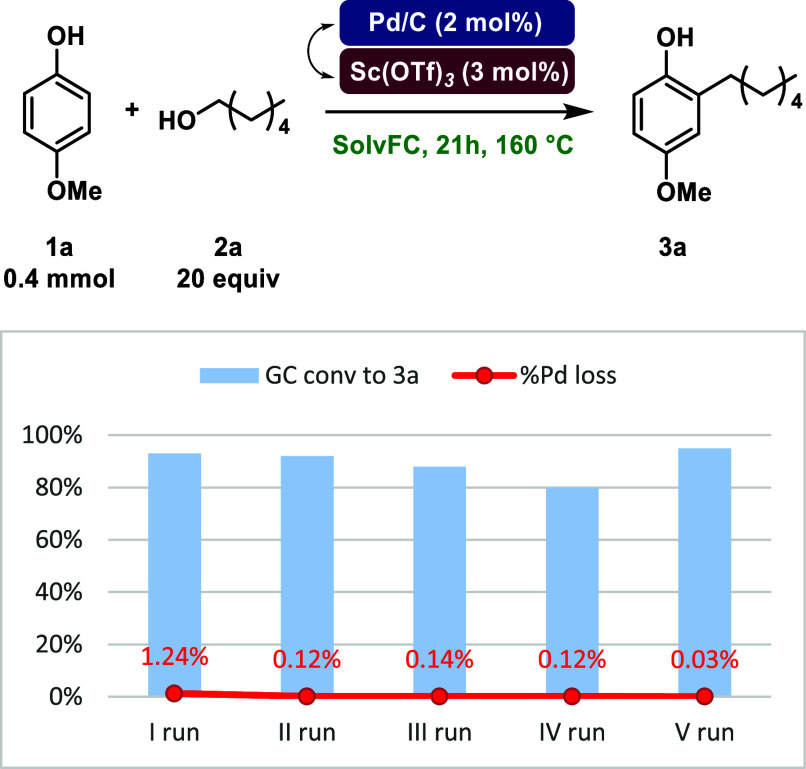
Recycling and Relative Leaching Tests of Pd/C

To prove that Pd/C retains the same morphology after five runs, we performed HR-TEM analysis of the catalyst (Figures 3 and S2–S5 in the Supporting Information). The images indicate that the nanoparticles exhibit a stable and narrow size distribution that remains largely unchanged after the recycling tests. Most nanoparticles have a diameter ranging from 1.5 to 3.5 nm; however, in the used catalyst, an increased number of nanoparticles with diameters between 3.5 and 5.5 nm was observed. This stability suggests that the recycle tests do not induce significant degradation of the nanoparticles, preserving their structural integrity.

**Figure 3:**
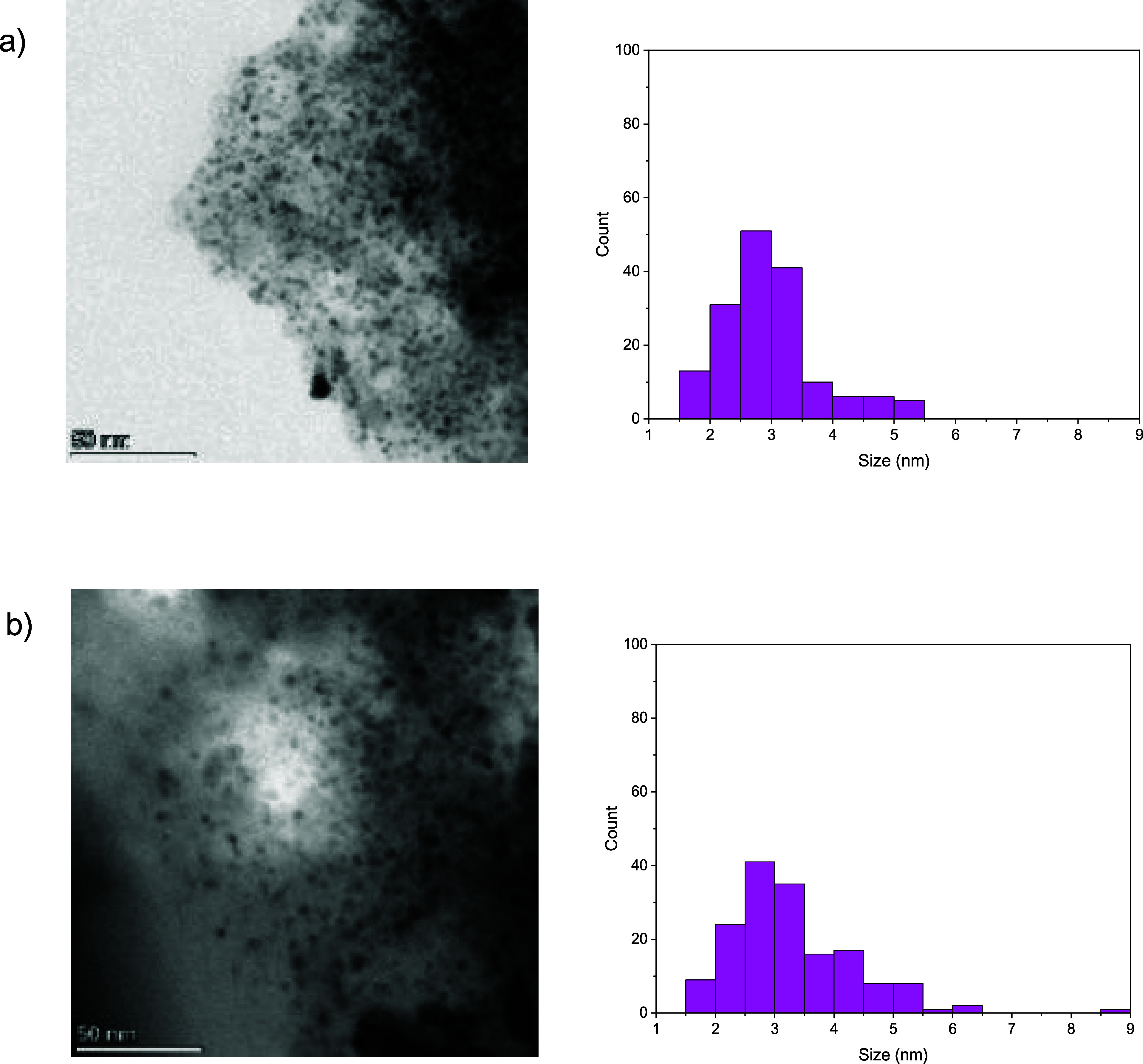
TEM images of the (a) preactivated catalyst before the reaction and (b) catalyst after 5 runs, with the relative nanoparticle size distributions.

We also performed XRD analysis (Figure S6): the Pd/C patterns exhibited the characteristic peaks of metallic palladium (Pd(111), Pd(200), Pd(220), Pd(311), and Pd(222)), which were preserved after 24 h of reaction. However, in agreement with the HR-TEM analysis, broader peaks were observed in the used catalyst compared to the freshly activated one, confirming an increase in the size of the metallic Pd nanoparticles. No significant crystallographic differences were observed between the commercial and activated Pd/C samples. Additionally, the broad signal at low angles reflects the amorphous nature of the activated charcoal support.

With these excellent results for the recyclability of Pd/C, we can now disclose the implementation of a flow protocol. The importance of the air atmosphere in this process is drastic, and several flow setups (such as the classical packed-bed reactor) led to completely unsatisfactory results (see Table S5). In agreement with the proposed mechanism, the delivery of the proper amount of air to the heterogeneous catalytic system is crucial. We therefore directed our attention to a tube-in-tube setup that we proved as an efficient approach for solid–liquid–gas conditions.^60^

The general assembly of the reactor is shown in Scheme S4, which consists of one small tubing inserted into a bigger one. The smallest tube (permeable to air) is where compressed air passes; the space between the bigger tube and the small one is packed with the Pd/C catalyst dispersed in quartz, where also the reaction mixture passes. Once the apparatus was set up, we optimized the residence time by increasing the reactor length (Table S6). The longer the reactor length, the higher the product yield, with up to 72% yield of 2-hexyl-3,5-dimethoxyphenol **3c**. We were able to form 1.4 g (5.8 mmol) of **3c** in a representative gram scale synthesis (Scheme 5). The newly defined system also resulted in low leaching, with only 0.42 ppm of Pd leached.

**Scheme 5:**
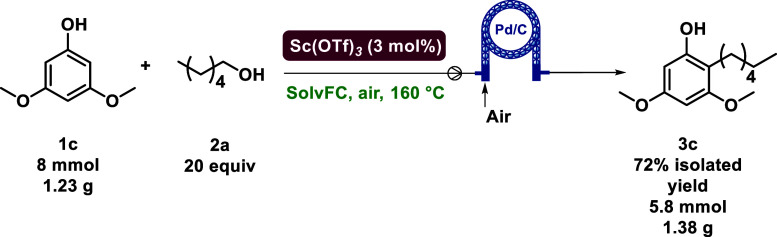
Flow Gram Scale-Up

Aiming to prove the feasibility of the flow protocol with different phenolic substrates, we also tested 3,4,5-trimethoxyphenol **1d** and 3-methoxyphenol **1b** with 1-hexanol **2a.** Scheme 6 displays the yields: an excellent 87% was obtained for product **3d** and a discrete 55% for product **3b**. We also tested whether changing the alcohol chain would affect the protocol by using 1-pentanol **2b**, 1-eptanol **2c**, 1-octanol **2d**, and iso-amyl alcohol **2f** with **1b** and **1d**. We observed that the flow protocol is applicable to all substrates with yields of up to 80%. These results demonstrate the applicability of the flow protocol to various functionalized reagents.

**Scheme 6:**
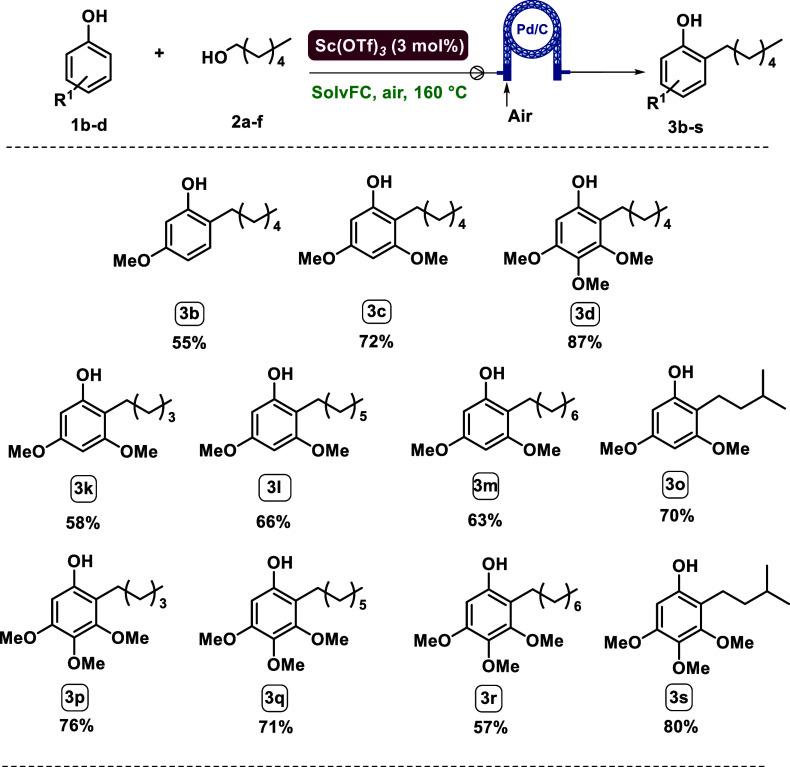
Different Phenolic Substrates Tested for the Flow Protocol

Finally, we compared the green metrics associated with this gram scale-up with previous protocols (Section 12 of the Supporting Information). We calculated the Environmental Factor (E-factor) of this work,^65^ both for the batch and the flow processes, which were 112 and 13, respectively. The main factor that makes the E-factor in the batch high (112) is the workup proportion, while in the flow process, this percentage is zero since no filtration is needed before column chromatography. We also performed a qualitative Chem21 evaluation^66,67^ : in our processes, we were able to minimize the red flags to 2 while other protocols have a minimum of 4 red flags. This is mostly due to the use of a safer coupling reagent such as 1-hexanol without employing hazardous or problematic solvents such as 1,2-dichloroethane, dichloromethane, or toluene. The current limitation of our protocol is the relatively high reaction temperature. However, such conditions are common in C–H functionalization chemistry and we were able to render it practical by the implementation of a flow setup. Future studies will focus on minimizing the thermal input.

## Conclusions

A cooperative dual catalytic system was developed to obtain a selective *ortho*-alkylation of phenols with primary alcohols combining the activity of heterogeneous Pd/C with a catalytic amount of Lewis acid Sc­(OTf)_3_. This efficient strategy permits the recovery and reuse of the heterogeneous catalyst up to 5 runs in the batch process (with only 1.66% of total Pd loss), and the implementation of a flow protocol with a tube-in-tube reactor for a gram scale synthesis, achieving a productivity of 0.6 g/h. It should be noted that this value is only representative, but it allows one to perform the process on a larger scale, which was not possible under batch conditions in our study due to the delicate influence of the oxygen headspace. Furthermore, the effectiveness of this protocol is demonstrated by the activity of multiple phenol substrates potentially derived from lignocellulosic biomass together with different primary alcohols. This innovative reaction can be also used for synthesizing pharmaceutically relevant molecules: amyl-meta-cresol was obtained in a single step, whereas it is usually formed through a three-step process, improving atom and step economy.

## Methods Section

### General Remarks

Reactions were performed with continuous magnetic stirring in a 12 mL screw-capped vial; dry conditions are not required. The Pd/C 10 wt % loading, matrix-activated carbon support was purchased from Sigma-Aldrich. Before running the reactions, Pd/C was activated under vacuum at 130 °C for at least 2 h. Phenols and primary alcohols were purchased from Sigma-Aldrich, Alfa Aesar, and Fluorochem and applied without purification. Sc­(OTf)_3_ was purchased from Fluorochem. GC analyses were performed using a Hewlett-Packard HP 5890A equipped with a capillary column DB-35MS (30 m, 0.53 mm), an FID detector, and helium as a gas carrier. GC-EIMS analyses were carried out using a Hewlett-Packard HP 6890N Network GC system/5975 Mass Selective Detector equipped with an electron impact ionizer at 70 eV. Melting points were measured on a Buchi 510 apparatus. ^1^H NMR and ^13^C NMR spectra were recorded on a Bruker DRX-ADVANCE 400 MHz (^1^H at 400 MHz, ^13^C at 100.6 MHz) using a convenient deuterated solvent (CDCl_3_). Chemical shifts are reported in ppm (δ), coupling constants (*J*) in Hertz, and multiplicity is reported as follows: s = singlet, bs = broad singlet, d = doublet, dd = double doublet, t = triplet, and m = multiplet. Pd leaching was measured using an Agilent MP-AES 4210 instrument. SEM analyses were performed using an FE-SEM LEO 1525 ZEIS. HR-TEM analyses were performed with a Thermo Scientific Talos F200X scanning/transmission electron microscope (S/TEM) with energy-dispersive X-ray spectroscopy signal detection. High-resolution synchrotron X-ray diffraction and total scattering measurements were performed at Beamline ID31 at the European Synchrotron Radiation Facility (ESRF). The sample powders were loaded into cylindrical slots (approximately 1 mm thickness) held between Kapton windows in a high-throughput sample holder. Each sample was measured in transmission geometry with an incident X-ray energy of 75.051 keV (λ = 0.16520 Å). The measured intensities were collected using a Pilatus CdTe 2 M detector (1679 × 1475 pixels, 172 × 172 μm^2^ each) positioned with the incident beam in the corner of the detector. The sample-to-detector distance was approximately 1.5 m for high-resolution measurements and 0.3 m for the total scattering measurements. The background for the empty windows was measured and subtracted. NISTSRM 660b (LaB6) was used for geometry calibration performed with the software pyFAI followed by image integration including flat-field, geometry, solid-angle, and polarization corrections. Column chromatography (FCC) was carried out on Merck silica gel 60 (230–400 mesh), and the solvent systems used are reported in parentheses.

### Experimental Procedures

#### General Procedure for the *ortho*-Alkylation of Phenols in Batch

In a 12 mL screw-cap vial equipped with a magnetic stir bar, 8.5 mg of Pd/C (2 mol %), 5.9 mg of Sc­(OTf)_3_ (3 mol %), and phenol (0.4 mmol) are weighed. Then, primary alcohol (20 equiv) is added, and the mixture is left stirring at 160 °C. Once the reaction is finished, the mixture is cooled to room temperature and filtered on a pad of silica gel with EtOAc to remove the catalyst and the Lewis acid. The excess of alcohol is separated from the filtrate by distillation, and the residue is purified by chromatographic column or by preparative thin-layer chromatography (TLC) using a variable ratio eluent mixture of ETP and EtOAc.

#### Catalyst Recycling

Pd/C was filtered off from the reaction mixture using a Hirsh funnel and washed with EtOAc (2 mL) and water (5 mL). The recovered catalyst was dried at 130 °C under vacuum for 3 h and reused without a significant change in weight.

#### Leaching Tests for the Reaction in Batch

After the reaction was completed, the heterogeneous catalyst was filtered off from the reaction mixture using a Hirsh funnel and washed with EtOAc (2 mL). The reaction mixture was dried under a vacuum, dissolved in 2 mL of aqua regia, and digested at room temperature. The reaction mixture was transferred into a 10 mL graduated flask, and Milli-Q water was added to reach the final volume. If present, the residual solid was filtered off, and the sample was analyzed by an MP-AES 4210 instrument.

#### General Procedure for the *ortho*-Alkylation of Phenols in Flow

The flow streams driven by the HPLC pump containing the solution of Sc­(OTf)_3_ (3 mol %, 118 mg), phenol (1 mmol or 8 mmol) and 1-hexanol (20 equiv) was directed through the tube-in-tube reactor packed with Pd/C (10% w/w, 344 mg, 3.8 mmol of Pd) dispersed in quartz (99 ww%) placed in a reactor installed in an aluminum brick at 160 °C at a pressure of 5 bar of compressed air with 0.5 mL/min flow rate. The reaction mixture was continuously pumped with a residence time inside the reactor of 348 min. The reaction mixture at the outlet of the reactor was collected into a flask, and 1-hexanol was removed via distillation under vacuum, and the crude mixture was purified by column chromatography.

#### Leaching Tests for Flow

At the outlet of the reactor, samples of the reaction mixture were taken and digested into 2 mL of aqua regia for 1h. The digested material was diluted with Milli-Q water to a final volume of 10 mL, and the amount of palladium leached in the solution was measured with a microwave plasma-atomic emission spectrometer (MP-AES 4210).

## Supplementary Material



## Notes

General remarks; experimental procedures (general procedure for the hydrodeoxygenation of phenols, general procedure for the hydrodeoxygenation of lignin, catalyst recycling); catalyst screening in basic conditions; catalysts screening in acidic conditions; scope of the reaction; kinetic studies; numeric analyses of kinetic data; TEM and SEM analyses; additional mechanistic studies; spectral data, and ^1^H NMR and ^13^C NMR spectra of isolated compounds (PDF)

## Abbreviations

Sc­(OTf)_3_: scandium trifluoromethanesulfonate
Pd/C: palladium on carbon
DNOC: 4,6-dinitro-ortho-cresol
SEAr: electrophilic aromatic substitution
TiO_2_: titanium dioxide
EDGs: electron-donating Groups
NPs: nanoparticles
HR-TEM: high-resolution scanning transmission electron microscopy
SEM: scansion electronic microscopy
XRD: X-ray diffraction
TLC: thin-layer chromatography
GC: gas chromatography
