# Supplementary material for: Cooperative Lewis Acid/Metal Dual Catalysis for the Selective ortho-Alkylation of Phenols
Source: ACS Sustain Chem Eng. 2025 Jul 25;13(30):12220–31. doi: 10.1021/acssuschemeng.5c04668 (PMC12326393; doi:10.1021/acssuschemeng.5c04668)
Supplement: Supplementary file 1 [file sc5c04668_si_001.pdf]

SUPPORTING INFORMATION FILE

Cooperative Lewis acid/metal dual catalysis for the selective ortho-alkylation of phenols

Benedetta Di Erasmo,[§a] Edoardo Bazzica,[§a] Giulia Brufani,[a] Chao-Jun Li,[b] Luigi Vaccaro* [a]

[a] *Laboratory of Green S.O.C. – Dipartimento di Chimica, Biologia e Biotecnologie, Università degli Studi di Perugia, Via Elce di Sotto 8, 06123, Perugia, Italy*.

[b] *Department of Chemistry, and FRQNT Centre for Green Chemistry and Catalysis – McGill University, 801 Sherbrooke Street West, Montreal, QC H3A0B8, Canada*

[§] Both authors contributed equally to this work.

e-mail: luigi.vaccaro@unipg.it

total number of pages: 67

total number of tables: 31

total number of figures: 26

total number of schemes: 4

Table of contents:

1. General Remarks …………………………………………………………………..……..S3

2. Experimental Procedures…………………………….……………………………..……..S4

   2.1. General procedure for the ortho-alkylation of phenols in batch……………........…S4

   2.2. Catalyst recycling……………………….………………………………..……..S4

   2.3. Leaching tests for the reaction in batch ……………………………..……..……..S4

   2.4. General procedure for the ortho-alkylation of phenols in flow……….………..……..S4

   2.5. Leaching tests for the reaction in flow ……….……………………..…..…….S5

3. Optimization of the Lewis Acid……….……….……….……….…….……..…….S5

4. Optimization of the heterogeneous Pd catalyst……….……….…….……….……..…….S6

5. Optimization of the reaction time ……………..……………………………..……..S6

6. Primary alcohols……………………….………………………………….……..……..S7

7. Other mechanistic controls……….……….……….……….……….……………..…….S7

8. Hot-filtration test………………………...…………………………………….……..S8

9. Recycle tests..……….……….……….……….……….……….……..…………………S9

10. HR-TEM and SEM images; XRD patterns ……….……….……….……….……..………S10

11. Flow procedure..…………….……….……….……….……….………..……………...S12

    9.1 General assembly of the reactor..……….……….……….……….…………...…S12

    9.2 Flow initial tests..…………….….………...……….……….………..………………...S13

12. Green metrics evaluation...…………………….……….……….……….…….……....S14

13. Spectral data of isolated compounds……..………….……….……..…….…………..S18

14. ^{1}H-NMR and ^{13}C-NMR spectra of isolated compounds……….……….……….……..………S38

15. Chem21 evaluation………………………………….….…………………………S58

1. General Remarks

Reactions were performed with continuous magnetic stirring in a 12 mL screw-capped vial; dry conditions are not required. Pd/C 10 wt.% loading, matrix activated carbon support was purchased from Sigma Aldrich. Before running the reactions, Pd/C is activated under vacuum at 130°C for at least 2 hours. Phenols and primary alcohols were purchased from Sigma-Aldrich, Alfa Aesar and Fluorochem and applied without purification. $Sc(OTf)_3$ was purchased from Fluorochem. GC analyses were performed using a Hewlett-Packard HP 5890A equipped with a capillary column DB-35MS (30 m, 0.53 mm), an FID detector, and helium as a gas carrier. GC-EIMS analyses were carried out using a Hewlett-Packard HP 6890N Network GC system/5975 Mass Selective Detector equipped with an electron impact ionizer at 70 eV. Melting points were measured on a Buchi 510 apparatus. ^1H NMR, ^{13}C NMR spectra were recorded on a Bruker DRX-ADVANCE 400 MHz (^1H at 400 MHz, ^{13}C at 100.6 MHz) using a convenient deuterated solvent ($CDCl_3$). Chemical shifts are reported in ppm (δ), coupling constants (J) in Hertz and multiplicity are reported as follows: s = singlet, bs = broad singlet, d = doublet, dd = double doublet, t = triplet, m = multiplet. Pd leaching was measured using an Agilent MP-AES 4210 instrument. SEM analyses were performed using FE-SEM LEO 1525 ZEIS. HR-TEM analyses were performed with a Thermo Scientific™ Talos™ F200X scanning/transmission electron microscope (S/TEM) with energy dispersive x-ray spectroscopy (EDS) signal detection. High-resolution synchrotron X-ray diffraction and total scattering measurements were performed at beamline ID31 at the European Synchrotron Radiation Facility (ESRF). The sample powders were loaded into cylindrical slots (approx. 1 mm thickness) held between Kapton windows in a high-throughput sample holder. Each sample was measured in transmission geometry with an incident X-ray energy of 75.051 keV (λ = 0.16520 Å). Measured intensities were collected using a Pilatus CdTe 2M detector (1679×1475 pixels, 172×172 μm2 each) positioned with the incident beam in the corner of the detector. The sample-to-detector distance was approximately 1.5 m for the high-resolution measurements and 0.3 m for the total scattering measurements. Background measurements for the empty windows were measured and subtracted. NISTSRM 660b (LaB6) was used for geometry calibration performed with the software pyFAI followed by image integration including a flat-field, geometry, solid-angle, and polarization corrections. Column chromatography (FCC) was carried out on Merck silica gel 60 (230 – 400 mesh) and the solvent systems used were reported in parenthesis.

2. Experimental procedures

2.1. General procedure for the *ortho*-alkylation of phenols in batch

In a 12 ml screw-cap vial equipped with a magnetic stir bar, 8.5 mg of Pd/C (2 mol%), 5.9 mg of $Sc(OTf)_3$ (3 mol%) and phenol (0.4 mmol) are weighed. Then, primary alcohol (20 eq, 1 mL) is added, and the mixture is left stirring at 160°C. Once the reaction is finished, the mixture is cooled to room temperature and filtered on a pad of silica gel with EtOAc to remove the catalyst and the Lewis acid. The excess of alcohol is separated from the filtrate by distillation and the residue is purified by chromatographic column or by preparative thin layer chromatography (TLC) using a variable ratio eluent mixture of ETP and EtOAc.

2.2. Catalyst recycling

In a 12 mL screw-cap vial equipped with a magnetic stir bar, 8.5 mg of Pd/C (2 mol%), 5.9 mg of $Sc(OTf)_3$ (3 mol%) and 49.6 mg of 4-methoxyphenol (0.4 mmol) are added. Then, 1 mL of 1-hexanol (20 eq) is added, and the mixture is kept stirring at 160°C for 20 h. Once the reaction is finished, the mixture is cooled to room temperature; Pd/C was filtered off from the reaction mixture using a Hirsh funnel and washed with EtOAc (5 mL) and water (5 mL). The recovered catalyst was dried at 130°C under vacuum for 3 h and reused without significant change in weight. Pd/C was filtered off from the reaction mixture using a Hirsh funnel and washed with EtOAc (2 mL) and water (5 mL). The recovered catalyst was dried at 130°C under vacuum for 3 h and reused without significant change in weight.

2.3. Leaching tests for the reaction in batch

After reaction completion, the heterogeneous catalyst was filtered off from the reaction mixture using a Hirsh funnel and washed with EtOAc (2 mL). The reaction mixture was dried under vacuum, dissolved in 2 mL of aqua regia, and digested at room temperature. The reaction mixture was transferred into a 10 mL graduated flask and Milli-Q water was added to reach the final volume. If present, residual solid was filtered off and the sample was analysed by MP-AES 4210 instrument.

2.4. General procedure for the *ortho*-alkylation of phenols in flow

The flow streams driven by the HPLC pump containing the solution of $Sc(OTf)_3$ (3 mol%, 118 mg), phenol (1 mmol or 8 mmol) and 1-hexanol (20 equiv) was directed through the tube-in-tube reactor packed with Pd/C (10% w/w, 344 mg, 3.8 mmol of Pd) dispersed in quartz (99 ww%) placed in a reactor installed in an aluminum brick at 160°C at a pressure of 5 Bar of compressed air with 0.5 mL/min flow rate. The reaction mixture was continuously pumped with a residence time inside the

reactor of 348 min. The reaction mixture at the outlet of the reactor was collected into a flask, 1-hexanol was removed via distillation under vacuum and the crude mixture was purified by column chromatography.

2.5. Leaching tests for flow

At the outlet of the reactor, samples of the reaction mixture were taken and digested into 2 mL of aqua regia for 1h. The digested material was diluted with milli-Q water to a final volume of 10 mL and the amount of palladium leached in solution was measured with a microwave plasma-atomic emission spectrometer (MP-AES 4210).

3. Optimization of the Lewis acid

Table S1. Optimization of the Lewis acid. [a]

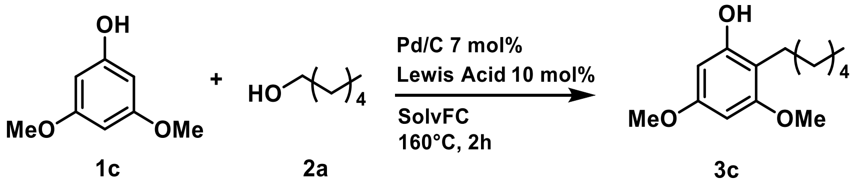

Entry	Lewis acid amount	GC conv to 3a (%)
1	-	0
2	$MnBr_2$ 10 mol%	15
3	$MnCl_2$ 10 mol%	0
4	Zr-propionate	0
5	$NiCl_2$ 10 mol%	0
6	NiI_2 10 mol%	0
7	$CuCl_2$ 10 mol%	3
8	$CuBr_2$ 10 mol%	0
9	$CoCl_2$ 10 mol%	1
10	$ZnCl_2$ 10 mol%	0
11	$FeCl_3 \cdot 6H_2O$ 10 mol%	34
12	$Fe(NO_3)_2 \cdot H_2O$ 10 mol%	0
13	$FeBr_2$ 10 mol%	19
14	$AlCl_3$ 10 mol%	0
15	$InBr_3$ 10 mol%	10
16	$Mg(ClO_4)_2$ 10 mol%	0
17	$K(ClO_4)$ 10 mol%	0
18	$Co(ClO_4)_2 \cdot 6H_2O$ 10 mol%	6
19	$Ni(ClO_4)_2 \cdot 6H_2O$ 10 mol%	16
20	$Zn(ClO_4)_2 \cdot 6H_2O$ 10 mol%	54
21	$Ag(ClO_4)$ 10 mol%	54
22	$Al(ClO_4)_3$ 10 mol%	55
23	$Y(OTf)_3$ 10 mol%	62
24	$Zn(OTf)_2$ 10 mol%	67
25	$Sc(OTf)_3$ 10 mol%	72

26	$Cu(OTf)_2$ 10 mol%	66
27	$In(OTf)_3$ 10 mol%	57
28	$Hf(OTf)_4$ 10 mol%	Only *para*-alkylated side product is formed

[a] Reaction conditions: **1a** (0.2 mmol), **2a** (20 equiv), Pd/C 7 mol%, Lewis acid 10 mol%, 160°C, 2 h. [b] Determined by GLC analysis, the remaining materials are unreacted **1c** and **2a**.

4. Optimization of the heterogeneous Pd catalyst

Table S2. Optimization of the Pd heterogeneous catalyst. [a]

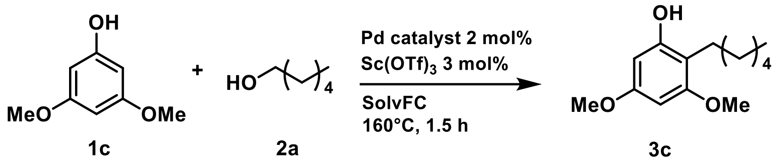

Entry	Pd catalyst	1a (%) [b]	3a (%) [b]
**1**	Pd/C (1 wt%) 2 mol%	40	60
**2**	Pd/C (5 wt%) 2 mol%	35	65
**3**	Pd/C (10 wt%) 2 mol%	34	66
**4**	$Pd/CaCO_3$ (1 wt%) 2 mol%	35	65

[a] Reaction conditions: **1a** (0.2 mmol), **2a** (20 equiv), Pd catalyst 2 mol%, Sc(OTf)3 3 mol%, 160°C, 2 h [b] Determined by GLC analysis.

5. Optimization of the reaction time

Table S3. Optimization of the reaction time.[a]

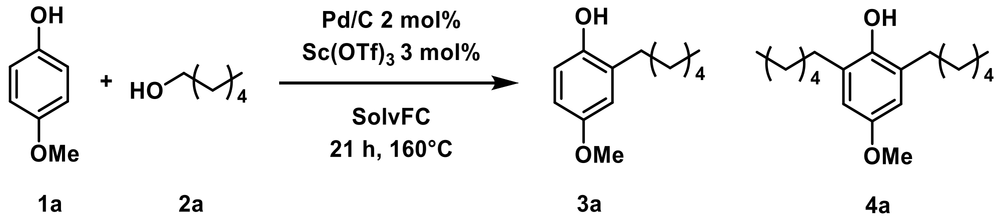

Entry	Reaction time	1a (%) [b]	3a (%) [b]	4a (%) [b]
**1**	30 min	> 99	-	-
**2**	2 h	90	10	0
**3**	6 h	66	44	0
**4**	16 h	39	61	0
**5**	20 h	7	93	0
**6**	24 h	4	77	19
**7**	30 h	0	42	58

[a] Reaction conditions: **1b** (0.2 mmol), **2a** (20 equiv), Pd/C 2 mol%, $Sc(OTf)_3$ 3 mol%,160°C, time, 12 mL vial. [b] Determined by GLC analysis.

6. Primary alcohols used in the substrate scope

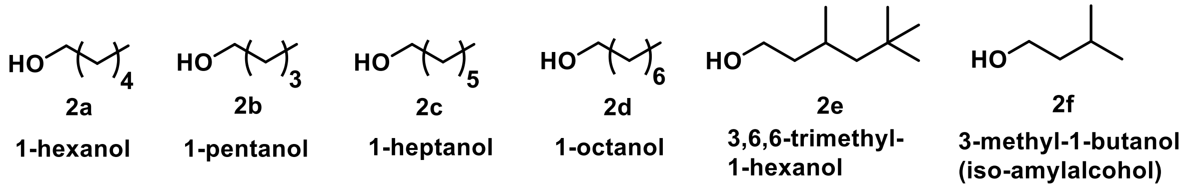

Scheme S1. Structure of the alcohols used in the substrate scope.

7. Other mechanistic controls

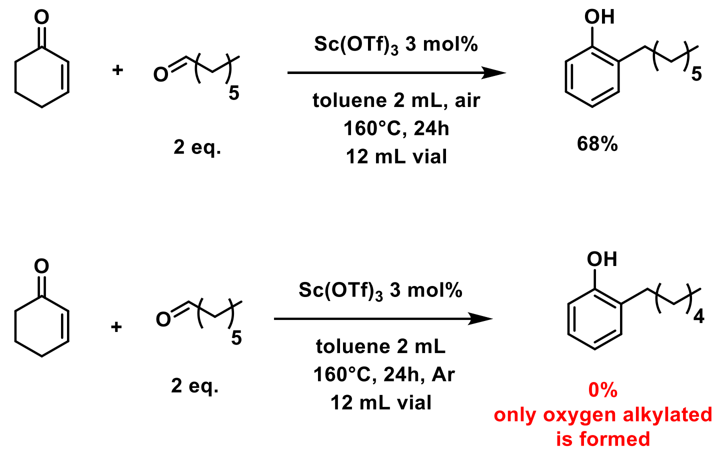

Scheme S2. Mechanistic controls between cyclohexenone and heptanal in different atmospheres.

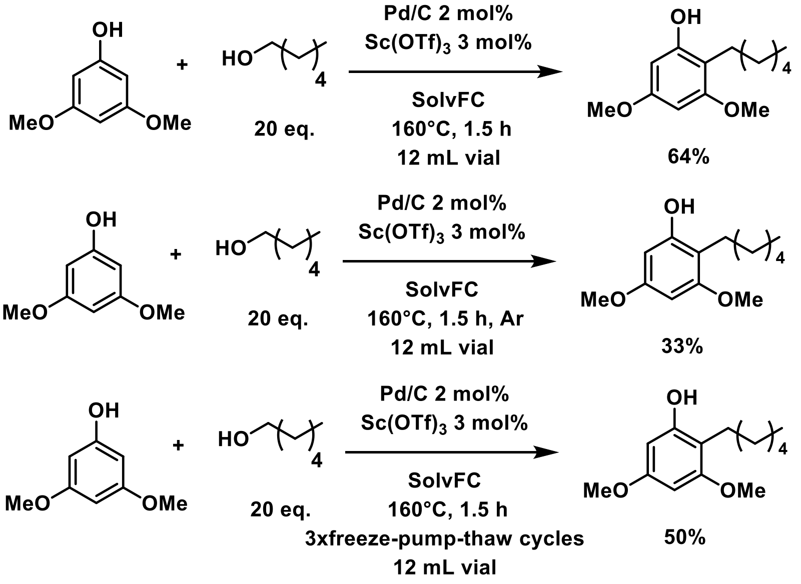

Scheme S3. Different atmospheres roles for the reaction between 3,5-dimethoxyphenol and 1-hexanol. Using argon has a detrimental role in this strategy since it flushes out the oxygen needed for the re-aromatization. Freeze-pump-thaw cycles maintain the extra-inert atmosphere that permits the Pd to re-aromatize the aldol adduct to our desired product. However, the yield is way lower than our conditions.

8. Hot filtration test

After 2 hours, the heterogeneous catalyst was filtered off from the reaction mixture using a Hirsh funnel to remove the solid support, while retaining any Pd nanoparticles in solution along with the Lewis acid. We obtained 10% towards the *ortho*-alkylated phenol. After filtration, the reaction was allowed to proceed for the remaining 18 hours. The reaction mixture was dried under vacuum, dissolved in 2 mL of aqua regia, and digested at room temperature. The reaction mixture was transferred into a 10 mL graduated flask and Milli-Q water was added to reach the final volume. If present, residual solid was filtered off and the sample was analyzed by MP-AES 4210 instrument. Obtaining a value of 16.4 ppm.

Figure S1. Reaction monitored over time in standard conditions vs hot filtration test. After 20h in standard conditions Pd leaching is 10.4 ppm while after the 2h hot filtration test is 16.4 ppm.

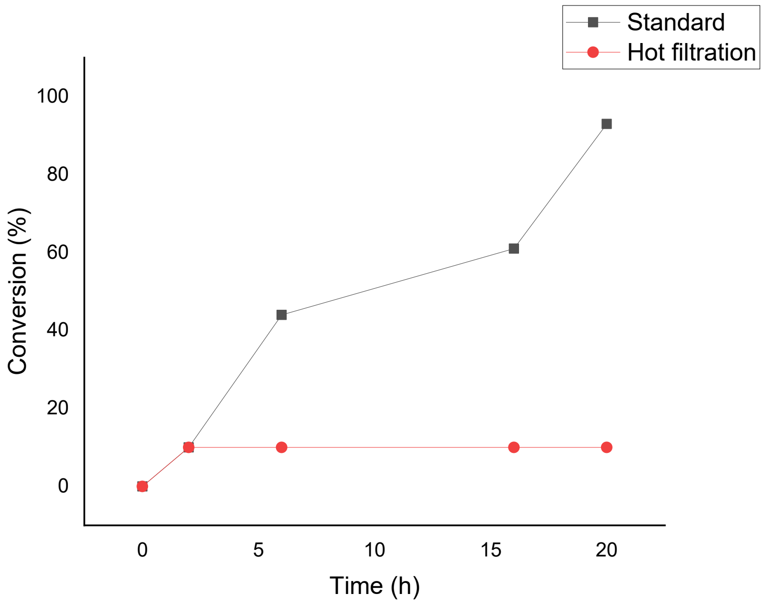

9. Recycle tests

Table S4. Recycle and relative leaching tests of Pd/C [a]

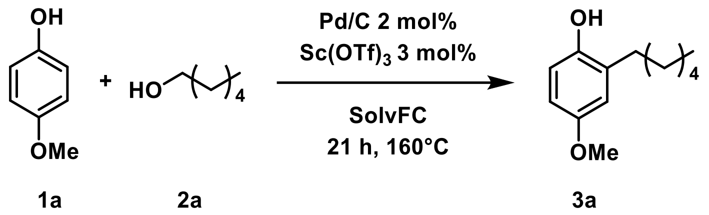

Run	3a (%) [b]	Leaching (ppm)	% Pd loss
**I**	93	10.48	1.24
**II**	92	1.05	0.12
**III**	88	1.23	0.14
**IV**	80	1.05	0.12
**V**	95	0.32	0.04
			1.66<br>TOTAL % of Pd loss

[a] Reaction conditions: **1a** (0.4 mmol), **2a** (20 equiv), Pd/C 2 mol%, $Sc(OTf)_3$ 3 mol%, 160°C, 20 h. [b] Determined by GLC analysis, the remaining materials are unreacted **1a** and **2a**.

10. HR-TEM and SEM images; XRD patterns

Figure S2. HR-TEM images of the fresh catalyst Pd/C prior pre-activation.

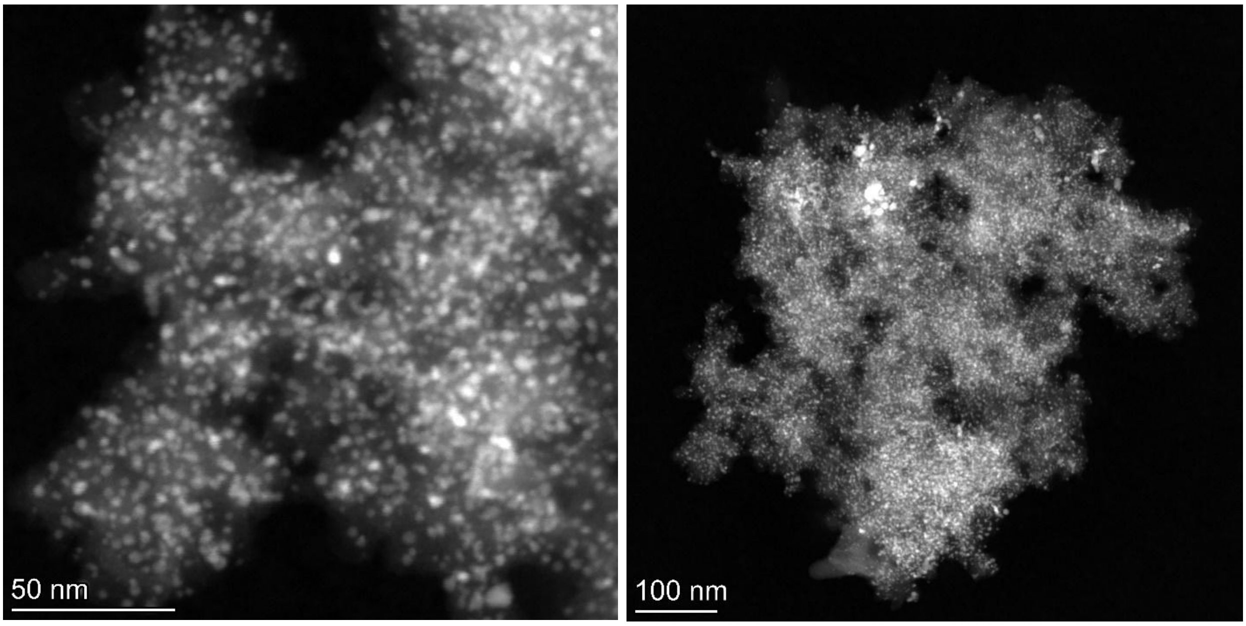

Figure S3. HR-TEM images of the catalyst Pd/C after pre-activation.
After pre-activation, we observe a more well-dispersed morphology; however, the size of the Pd nanoparticles remains unchanged.

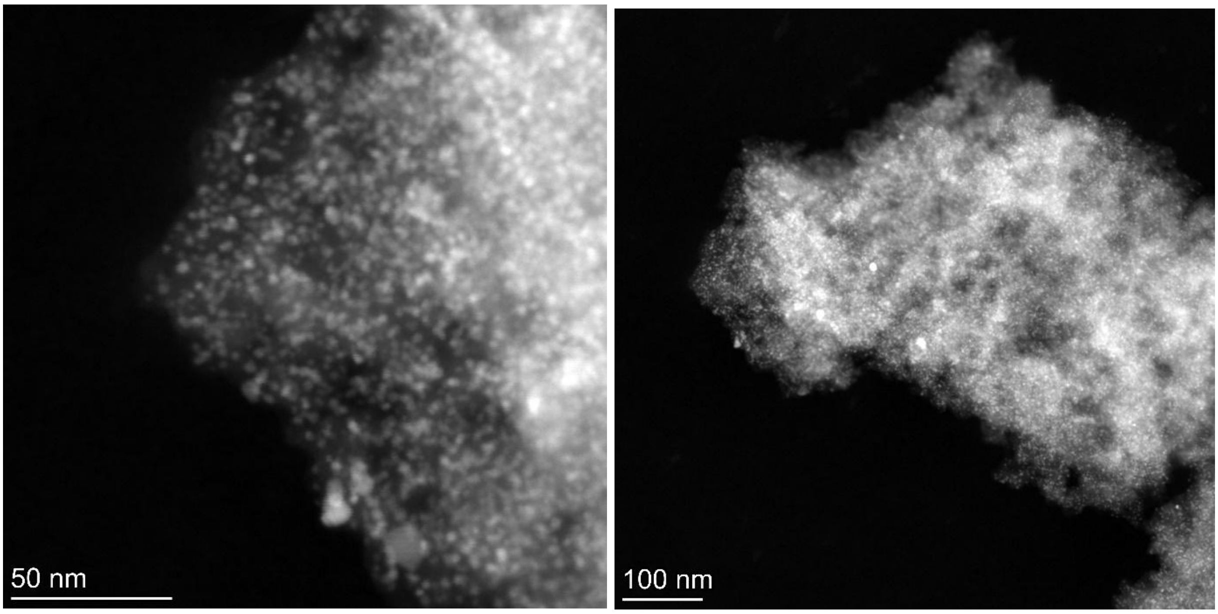

Figure S4. (a) HR-TEM and (b) SEM images of the catalyst Pd/C after 1 run.
The nanoparticles size and morphology remain the same as before the reaction.

a)
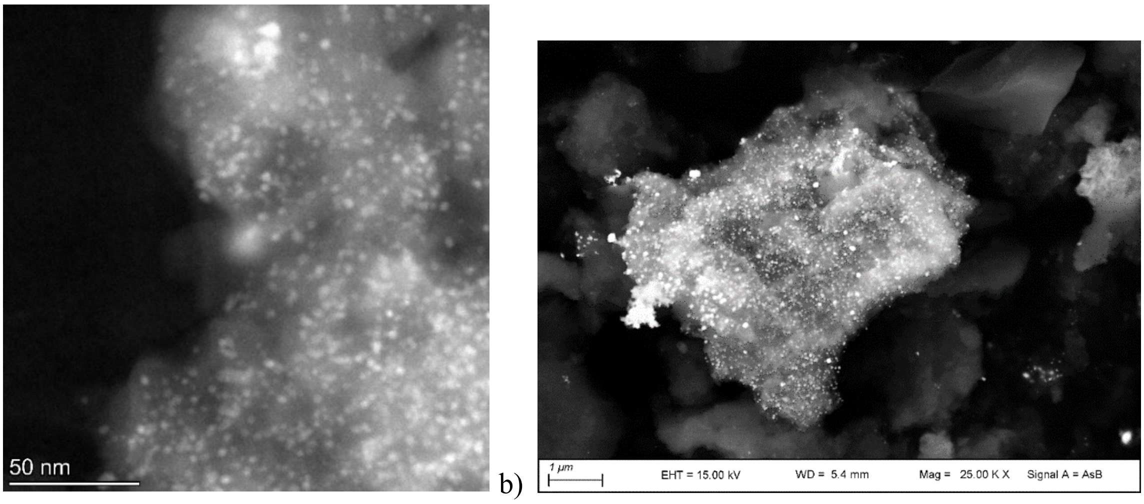

Figure S5. (a) SEM and (b) (c) HR-TEM images of the catalyst Pd/C after 5 runs.

After 5 runs, there are some nanoparticles aggregations, as we can see in **Figure S5b.** Nevertheless, some parts remain well dispersed with small nanoparticles size (**Figure S5a,S5c**).

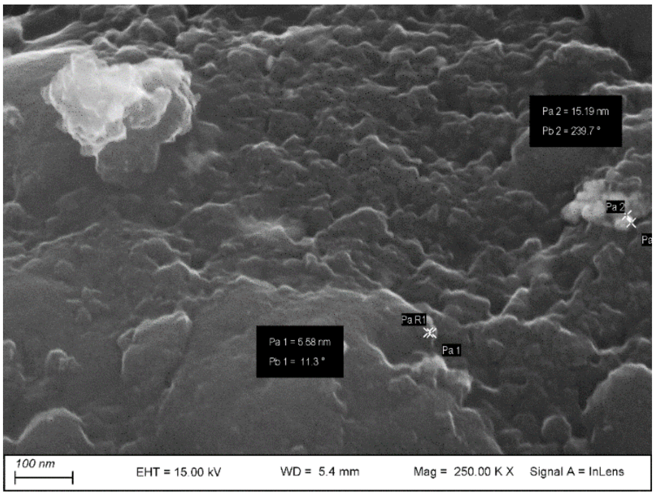

a)

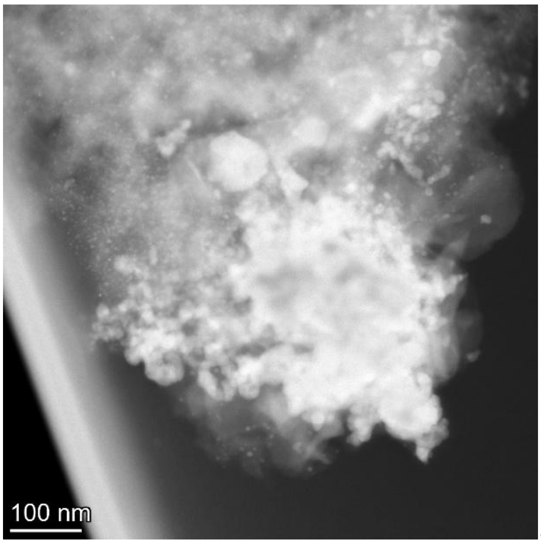

b)

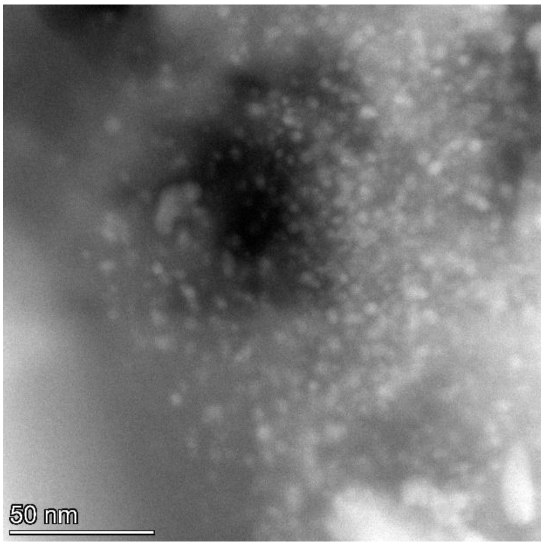

c)

Figure S6. XRD patterns of the commercial, the activated catalyst and after the first run. The same profile is maintained through all the 3 analyses proving the structural stability of the Pd specie.

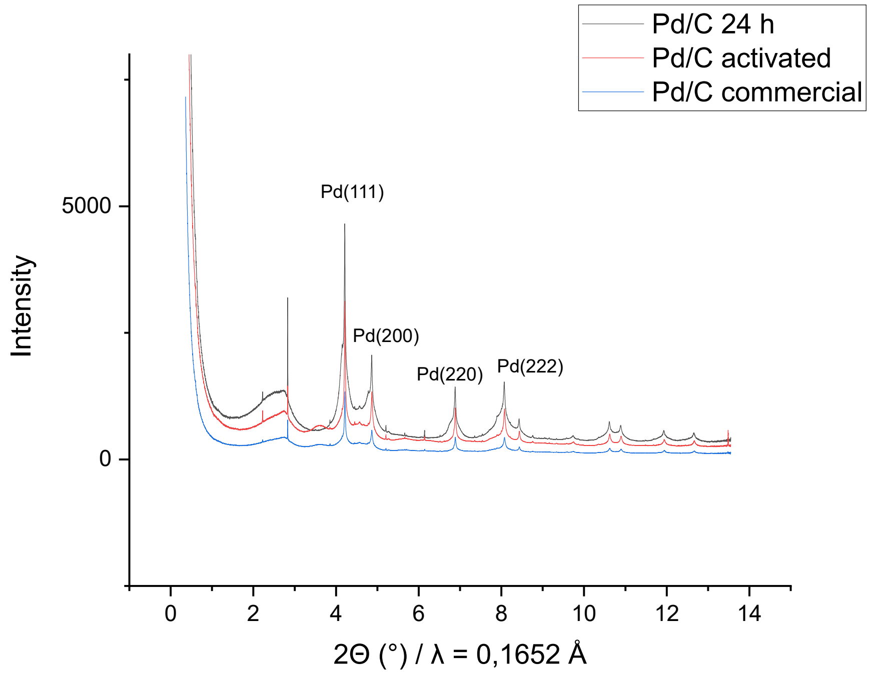

11. Flow procedure

11.1 General assembly of the reactor

The solid-liquid-gas reactor assembly was implemented with a 152 cm section of Teflon AF2400 tubing (with an internal diameter of 0.8 mm) placed within PTFE tubing (6 mm o.d., 3 mm i.d., 38 cm length). Each end was fastened to the proper Swagelok fitting (**Scheme S3**). The liquid inlet section was connected to a stainless-steel T-piece in which the compressed air was supplied. The outlet section was mounted with another T-piece stainless steel connector. The heterogeneous catalyst (Pd/C, 10% w/w) dispersed in quartz (ratio quartz/Pd/C 99:1) was packed between the 6 mm PTFE tubing and the Teflon AF2400 tube. The amount of catalyst used in the optimized conditions is 344 mg.

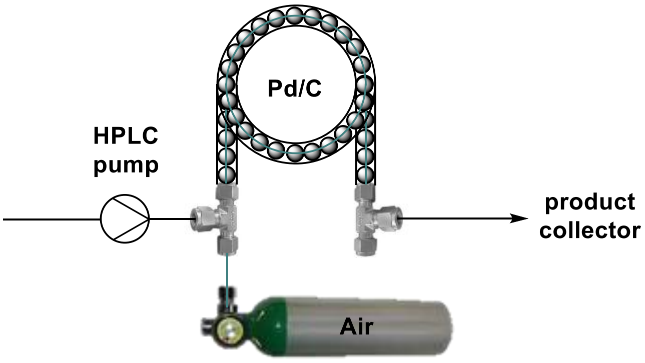

Scheme S4. Assembly of the reactor.

11.2 Flow protocol initial tests

Table S5. Flow tests with different reactors set-up. [a]

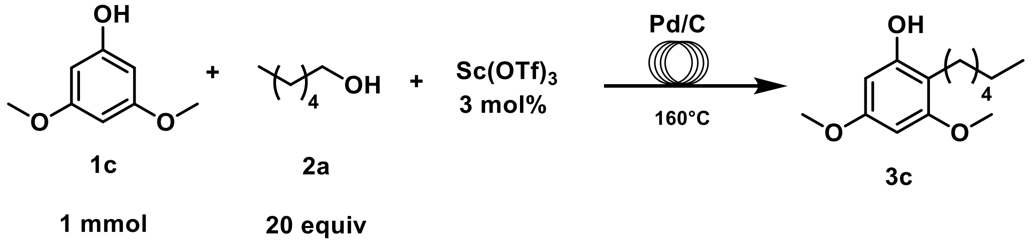

Entry	Set-up	Residence time	GC Conv in 3a(%). [b]
1	HPLC pump + packed-bed reactor with Pd/C (0.9 mmol) dispersed in quartz (7 w/w%)	27 min	**0**
2	HPLC pump + packed-bed reactor with Pd/C (0.9 mmol) dispersed in quartz (7 w/w%) (*cyclic loop*)	250 min	**0**
3	HPLC pump + packed-bed reactor with Pd/C (0.8 mmol) dispersed in quartz (7 w/w%)	200 min	**0**
4	Compressed air + packed-bed reactor with Pd/C (0.2 mmol) dispersed in quartz (7 w/w%)	350 min	**9**
5	HPLC pump + Tube-in-tube reactor with Pd/C (0.8 mmol)	27 min	**43**

[a] Reaction conditions: **1a** (1 mmol), **2a** (20 equiv), $Sc(OTf)_3$ (3 mol%), 160 °C, flow rate = 0.5 mL/min. . [b] Determined by GLC analysis, the remaining materials are unreacted **1c** and **2a**.

Table S6. Flow tests with reactor different lengths. [a]

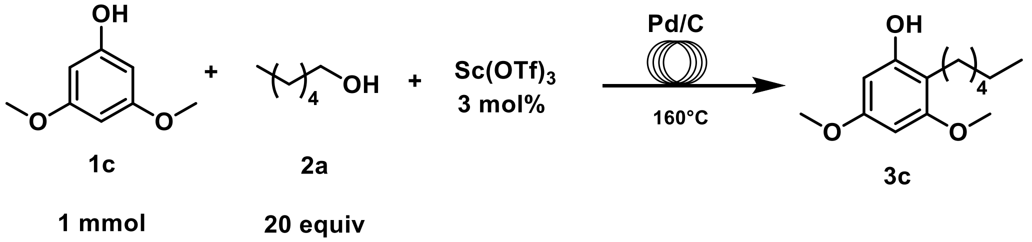

Entry	Reactor length	Residence time	GC Conv to 3c (%)[b]
1	38 cm	87 min	**43**
2 [c]	76 cm	174 min	**58**
3 [d]	152 cm	348 min	**77 (72)**

[a] Reaction conditions: **1a** (1 mmol), **2a** (20 equiv), Pd/C (0.9 mmol, 86 mg), $Sc(OTf)_3$ (3 mol%), the reactor is installed in an aluminium brick at 160°C, flow rate = 0.5 mL/min. [b] Determined by GLC analysis, the remaining materials are unreacted **1c** and **2a**. Yield of **3c** is reported in parenthesis. [c] Pd/C (1.8 mmol, 172 mg) [d] Pd/C (3.6 mmol, 344 mg)

12. Green Metrics evaluation

Herein we provide calculation of the E-factor, Atom Economy and Chem21 evaluation. It is to be noted that all the protocols require column chromatography so it has not been taken in consideration in the E-factor calculations.

For Chem21 analysis, since the yields are in the same range for all the protocols, we did not take them in consideration. The Excel files of the evaluation are provided in a separate Supporting Information file.

- *J. Am. Chem. Soc.* **2018**, *140*, 32, 10289–10296 (scale 0.5 mmol)

In a glove box, 3,5-dimethoxyphenol (0.5 mmol, 77 mg), 2-pentanone (1.0 mmol, 86 mg), and $[(C_6H_6)(PCy_3)(CO)RuH]^+ BF_4^-$ (9 mg, 3 mol %) were dissolved in 1,2- dichloroethane (2 mL) in a 25 mL Schlenk tube equipped with a Teflon stopcock and a magnetic stirring bar. The tube was brought out of the glove box and was stirred in an oil bath preset at 125°C for 24 h. The reaction tube was taken out of the oil bath and was cooled to room temperature. After the tube was opened to air, the solution was filtered through a short silica gel column by eluting with CH_2Cl_2 (10 mL), and the filtrate was analyzed by GC-MS. Analytically pure product was isolated by column chromatography on silica gel (230-460 mesh, hexanes/EtOAc).

No Health&Safety information is provided for the Ru catalyst.

Reactants	
3,5-dimethoxyphenol (MW = 154 g/mol)	0.077 g
2-pentanone (MW = 86.13 g/mol)	0.086 g
Catalysts and additives	
$(C_6H_6)(PCy_3)(CO)RuH]^+BF_4^-$	0.009 g
Reaction medium	
1,2-dichloroethane	2.5 g
Work-up	
Dichloromethane	13.3 g
Target product	
2-pentyl-3,5-dimethoxyphenol (224 g/mol)	0.061 g
E-factor	261
Atom Economy	93.3
Chem 21 evaluation	6 (red) 5 (yellow) 2 (green)

- *Angew. Chem. Int. Ed.* **2021**, *60*, 4043 (scale 0.2 mmol)

An oven-dried microwave reacting tube (10.0 mL) was charged with a magnetic stirbar, Pd/C (10 wt%, 15.0 mg, 7 mol% based on Pd contents, vacuum drying under reduced pressure for six hours) and lithium tert-butoxide (1.6 mg, 0.02 mmol). Then, toluene (1.0 mL), phenol (0.2 mmol) and alcohol (0.4 mmol) were added. The tube was sealed with rubber plug and evacuated by three freeze-pump-thaw cycles and backfilled with ultra-purified argon. Replace the rubber plug with an aluminum cover having a teflon pad. The tube was placed in a preheated oil bath at 160 oC and the mixture was stirred vigorously for 24 h. The reaction mixture was cooled to room temperature and

filtered through the pad of silica gel. The filtrate was concentrated and the resulting residue was purified via the column chromatography

In this work, the quantity of purification material employed was not reported; therefore, it was assumed to be equivalent to the amount utilized in our experimental setup. Column chromatography was used to purify the product.

Reactants	
1-hexanol (MW = 102.2 g/mol)	0.0408 g
Phenol (MW = 94 g/mol)	0.0188 g
Catalysts and additives	
Pd/C	0.015 g
t-BuOLi	0.0016 g
Reaction medium	
toluene	0.867 g
Work-up	
Ethyl acetate	7 g
Target product	
2-hexyl-phenol (MW = 179 g/mol)	0.030 g
E-factor	264
Atom Economy	91.3
Chem 21 evaluation	4 ● 5 ● 3 ●

- *J. Am. Chem. Soc.* **2021**, *143,* 33, 13428–13440 (scale 1 mmol)

In a glove box, complex 1 (18 mg, 3 mol %) was dissolved in 1,2-dichloroethane (0.5 mL) in a 25 mL Schlenk tube equipped with a Teflon stopcock and a magnetic stirring bar. 3-Methoxyphenol (124 mg, 1.0 mmol), 1-hexanal (100 mg, 1.0 mmol), 2-propanol (90 mg, 1.5 mmol) and 1,2-dichloroethane (0.5 mL) were added to the reaction tube. After the tube was sealed, it was brought out of the glove box and was stirred in an oil bath set at 120 °C for 12 h. The reaction tube was taken out of the oil bath and was cooled to room temperature. The product was isolated by a simple column chromatography on silica gel (40-63 μm particle size, hexanes/EtOAc = 80:1 to 10:1).

No Health&Safety information provided for the catalyst.

Reactants	
1-hexanal (MW = 100.16 g/mol)	0.1 g
3-methoxyphenol (MW = 124.14 g/mol)	0.124 g
Catalysts and additives	
$(C_6H_6)(PCy_3)(CO)RuH]^+BF_4^-$	0.018 g
2-propanol	0.09 g
Reaction medium	
1,2-dichloroethane	0.625 g
Work-up	
Dichloromethane	13.3 g
Target product	
1-hexyl-3-methoxyphenol (MW =208 g/mol)	0.147 g
E-factor	96

Atom Economy	92.7
Chem 21 evaluation	6 🔴 6 🟡 2 🟢

- this work in batch recovering catalyst and alcohol (scale 0.4 mmol)

In a 12 mL screw-cap vial equipped with a magnetic stir bar, 8.5 mg of Pd/C (2 mol%), 5.9 mg of $Sc(OTf)_3$ (3 mol%) and 49.6 mg of 4-methoxyphenol (0.4 mmol) are added. Then, 1 mL of 1-hexanol (20 eq) is added, and the mixture is kept stirring at 160°C for 20 h. Once the reaction is finished, the mixture is cooled to room temperature; Pd/C was filtered off from the reaction mixture using a Hirsh funnel and washed with EtOAc (5 mL) The excess of alcohol is separated from the filtrate by distillation and the residue is purified by chromatographic column using a variable ratio eluent mixture of ETP and EtOAc. The purified product is obtained as yellow oil (75% isolated yield, 62 mg). Pd/C is further washed with 5 mL water. The recovered catalyst was dried at 130°C under vacuum for 3 h and reused without significant change in weight.

Reactants	
4-methoxyphenol (MW = 124.14 g/mol)	0.0496 g
1-hexanol (MW = 102.177)	0.814 g
Catalysts and additives	
Palladium on carbon	0.0085 g
Scandium triflate	0.0059 g
Work-up	
Ethyl acetate	1.8 g
Water	5 g
Recycling-Recovery Materials	
Pd/C	0.0085 g
1-hexanol	0.659 g
Target product	
1-hexyl-4-methoxyphenol (MW = 208 g/mol)	0.062 g
E-factor	112
Atom Economy	91.9
Chem 21 evaluation	2 🔴 3 🟡 9 🟢

- this work in flow (scale 8 mmol)

The flow streams driven by the HPLC pump containing the solution of $Sc(OTf)_3$ (3 mol%, 118 mg), 3,5-dimethoxyphenol (8 mmol, 1.232 g) and 1-hexanol (20 equiv, 20 mL) was directed through the tube-in-tube reactor packed with Pd/C (10% w/w, 344 mg, 3.8 mmol of Pd) dispersed in quartz (99 ww%) placed in a reactor installed in an aluminium brick at 160°C at a pressure of 5 Bar of compressed air with 0.5 mL/min flow rate. The reaction mixture was continuously pumped with a residence time inside the reactor of 348 min. The reaction mixture at the outlet of the reactor was collected into a flask, 1-hexanol was removed via distillation under vacuum and the crude mixture was purified by column chromatography.

Reactants	
3,5-dimethoxyphenol (MW = 154 g/mol)	1.2 g
1-hexanol (MW = 102.177 g/mol)	16.28 g
Catalyst and additive	

Scandium triflate	0.118 g
Work-up	
Work-up materials	0 g
Target product	
1-hexyl-3,5-dimethoxyphenol (MW = 238 g/mol)	1.3 g
E-factor	13
Atom Economy	93.0
Chem 21 evaluation	2 🔴 1 🟡 10 🟢

13. Spectral data of isolated compounds

Table S7. Spectral data of 2-hexyl-4-methoxyphenol (**3a**).

**Chem. Name**	**2-hexyl-4-methoxyphenol**
**Lit. Ref.**	*none*

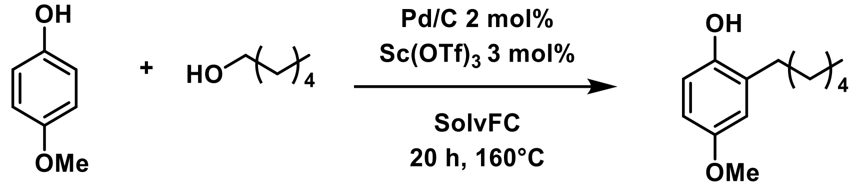

1a **2a** **3a**

METHOD:

In a 12 mL screw-cap vial equipped with a magnetic stir bar, 8.5 mg of Pd/C (2 mol%), 5.9 mg of $Sc(OTf)_3$ (3 mol%) and 49.6 mg of 4-methoxyphenol (0.4 mmol) are added. Then, 1 mL of 1-hexanol (20 eq) is added, and the mixture is kept stirring at 160°C for 20 h. Once the reaction is finished, the mixture is cooled to room temperature; Pd/C and $Sc(OTf)_3$ are filtered with EtOAc on a pad of silica gel. The excess of alcohol is separated from the filtrate by distillation and the residue is purified by chromatographic column using a variable ratio eluent mixture of ETP and EtOAc. The purified product is obtained as yellow oil (75% isolated yield, 62 mg).

**Mol Formula**		$C_{13}H_{20}O_2$	**m.p.**	Yellow oil
	**δ value**	**No. H**	**Mult.**	**J value/Hz**
**^{1}H NMR 400 MHz $CDCl_3$**	6.72-6.67	2	*m*	
	6.62	1	*d*	8.0
	4.37	1	*s*	
	3.76	3	*s*	
	2.57	2	*t*	8.0
	1.65-1.57	2	*m*	
	1.41-1.24	6	*m*	
	0.81-0.93	3	*m*	

^{13}C NMR (100.6 MHz, $CDCl_3$) δ: 153.8, 147.5, 129.9, 116.0, 115.9, 111.7, 55.8, 31.9, 30.4, 29.9, 29.3, 22.8, 14.2.

GC-EIMS (m/z, %): 208 (M+, 68), 138 (43), 137 (100), 77 (15).

**Table S8.** Spectral data of 2-hexyl-3-methoxyphenol (**3b**).	
**Chem. Name**	**2-hexyl-3-methoxyphenol (3b)**
**Lit. Ref.**	J. Yu, C. J. Li, H. Zeng, *Angew. Chem. Int. Ed.* **2021**, *60*, 4043–4048.

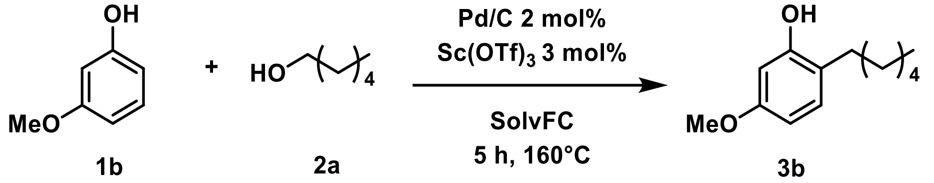

METHOD:

In a 12 mL screw-cap vial equipped with a magnetic stir bar, 8.5 mg of Pd/C (2 mol%), 5.9 mg of $Sc(OTf)_3$ (3 mol%) and 49.6 mg of 3-methoxyphenol (0.4 mmol) are added. Then, 1 mL of 1-hexanol (20 eq) is added, and the mixture is kept stirring at 160°C for 5 h. Once the reaction is finished, the mixture is cooled to room temperature; Pd/C and $Sc(OTf)_3$ are filtered with EtOAc on a pad of silica gel. The excess of alcohol is separated from the filtrate by distillation and the residue is purified by chromatographic column using a variable ratio eluent mixture of ETP and EtOAc. The purified product is obtained as yellow oil (60% isolated yield, 50 mg).

**Mol Formula**		$C_{13}H_{20}O_2$	**m.p.**	Yellow oil
	**δ value**	**No. H**	**Mult.**	**j value/Hz**
**^{1}H NMR 400 MHz $CDCl_3$**	7.02	1	*d*	8.3
	6.44	1	*dd*	8.3, 2.4
	6.37	1	*d*	2.4
	4.68	1	*s*	
	3.76	3	*s*	
	2.52	2	*t*	7.6
	1.60-1.54	2	*m*	
	1.36-1.27	6	*m*	
	0.88	3	*m*	

^{13}C NMR (100.6 MHz, $CDCl_3$) δ: 159.0, 154.3, 130.6, 120.8, 106.1, 101.8, 55.4, 31.9, 30.1, 29.4, 29.3, 22.8, 14.2.

GC-EIMS (m/z, %): 208 (M+, 10), 137 (100), 77 (16).

Table S9. Spectral data of 2-hexyl-3,5-dimethoxyphenol (**3c**).

**Chem. Name**	**2-hexyl-3,5-dimethoxyphenol (3c)**
**Lit. Ref.**	J. Yu, C. J. Li, H. Zeng, *Angew. Chem. Int. Ed.* **2021**, *60*, 4043–4048.

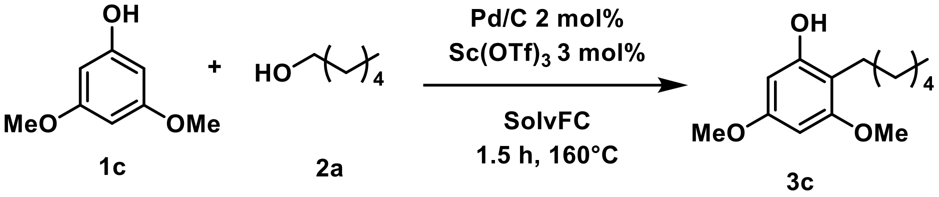

METHOD:

In a 12 mL screw-cap vial equipped with a magnetic stir bar, 8.5 mg of Pd/C (2 mol%), 5.9 mg of $Sc(OTf)_3$ (3 mol%) and 61.6 mg of 3,5-dimethoxyphenol (0.4 mmol) are added. Then, 1 mL of 1-hexanol (20 eq) is added, and the mixture is kept stirring at 160°C for 20 h. Once the reaction is finished, the mixture is cooled to room temperature; Pd/C and $Sc(OTf)_3$ are filtered with EtOAc on a pad of silica gel. The excess of alcohol is separated from the filtrate by distillation and the residue is purified by preparative TLC using 9:1 ETP:EtOAc. The purified product is obtained as brown oil (64% isolated yield, 61 mg).

**Mol Formula**		$C_{14}H_{22}O_3$	**m.p.**	Brown oil
**^{1}H NMR 400 MHz $CDCl_3$**	**δ value**	**No. H**	**Mult.**	**J value/Hz**
	6.09	1	*m*	
	6.04	1	*m*	
	4.91	1	*s*	
	3.77	3	*s*	
	3.75	3	*s*	
	2.53	2	*t*	7.4
	1.52-1.42	2	*m*	
	1.39-1.25	6	*m*	
	0.88	3	*t*	6.1

^{13}C NMR (100.6 MHz, $CDCl_3$) δ: 159.1, 158.9, 154.7, 109.5, 93.3, 91.4, 55.6, 55.3, 31.8, 29.5, 29.4, 22.7, 22.7, 14.1.

GC-EIMS (m/z, %): 238 (M+, 42), 168 (28), 167 (100), 137 (27), 109 (21)

Table S10. Spectral data of 2-hexyl-3,4,5-trimethoxyphenol (**3d**).

**Chem. Name**	**2-hexyl-3,4,5-trimethoxyphenol (3d)**
**Lit. Ref.**	G. Brufani, F. Valentini, F. Sabatelli, B. Di Erasmo, A. M. Afanasenko, C. J. Li, L. Vaccaro, *Green Chem.* **2022**, *24*, 9094.

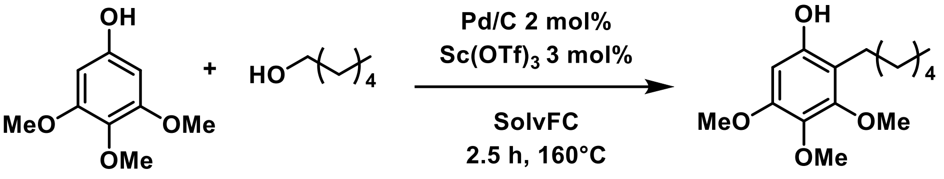

1d **2a** **3d**

METHOD:

In a 12 mL screw-cap vial equipped with a magnetic stir bar, 8.5 mg of Pd/C (2 mol%), 5.9 mg of $Sc(OTf)_3$ (3 mol%) and 73.6 mg of 3,4,5-trimethoxyphenol (0.4 mmol) are added. Then, 1 mL of 1-hexanol (20 eq) is added, and the mixture is kept stirring at 160°C for 2,5 h. Once the reaction is finished, the mixture is cooled to room temperature; Pd/C and $Sc(OTf)_3$ are filtered with EtOAc on a pad of silica gel. The excess of alcohol is separated from the filtrate by distillation and the residue is purified by chromatographic column using a variable ratio eluent mixture of ETP and EtOAc. The purified product is obtained as yellow oil (84% isolated yield, 76 mg).

**Mol Formula**	$C_{15}H_{24}O_4$	**m.p.**	Yellow oil	
	**δ value**	**No. H**	**Mult.**	**j value/Hz**
**^{1}H NMR 400 MHz $CDCl_3$**	6.21	1	*s*	
	4.60	1	*s*	
	3.87	3	*s*	
	3.80	6	*s*	
	2.52	2	*t*	7.7
	1.50-1.46	2	*m*	
	1.36-1.30	6	*m*	
	0.88-0.87	3	*m*	

^{13}C NMR (100.6 MHz, $CDCl_3$) δ: 152.5, 151.8, 149.8, 136.4, 114.4, 96.0, 61.3, 61.0, 56.0, 31.9, 30.3, 29.7, 23.8, 22.8, 14.2.

GC-EIMS (m/z, %): 268 (M+, 70), 253 (38), 197 (100), 182 (50), 139 (16).

Table S11. Spectral data of 2-hexyl-4-methylphenol (**3e**).

**Chem. Name**	**2-hexyl-4-methylphenol (3e)**
**Lit. Ref.**	*none*

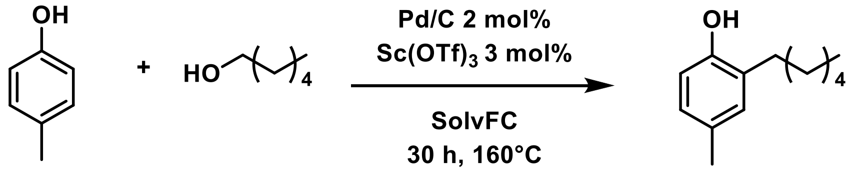

1e **2a** **3e**

METHOD:

In a 12 mL screw-cap vial equipped with a magnetic stir bar, 8.5 mg of Pd/C (2 mol%), 5.9 mg of $Sc(OTf)_3$ (3 mol%) and 41.8 mg of 4-methylphenol (0.4 mmol) are added. Then, 1 mL of 1-hexanol (20 eq) is added, and the mixture is kept stirring at 160°C for 30 h. Once the reaction is finished, the mixture is cooled to room temperature; Pd/C and $Sc(OTf)_3$ are filtered with EtOAc on a pad of silica gel. The excess of alcohol is separated from the filtrate by distillation and the residue is purified by TLC preparative using 98:2 ETP:EtOAc as eluent. The purified product is obtained as yellow oil (50% isolated yield, 38 mg).

**Mol Formula**		$C_{13}H_{20}O$	**m.p.**	Yellow oil
	**δ value**	**No. H**	**Mult.**	**J value/Hz**
**^{1}H NMR 400 MHz $CDCl_3$**	6.92	1	*s*	
	6.87	1	*d*	8.0
	6.66	1	*d*	8.0
	4.57	1	*s*	
	2.55	2	*m*	
	2.26	3	*s*	
	1.64-1.56	2	*m*	
	1.39-1.26	6	*m*	
	0.89	3	*m*	

^{13}C NMR (100.6 MHz, $CDCl_3$) δ: 151.3, 130.9, 130.0, 128.5, 127.5, 115.2, 31.9, 30.1, 30.0, 29.4, 22.8, 20.7, 14.3

GC-EIMS (m/z, %): 192 (M+,49), 122 (34), 121 (100), 91 (24), 77 (20).

**Table S12.** Spectral data of 2-hexyl-5-methylphenol (**3f**).	
**Chem. Name**	**2-hexyl-5-methylphenol (3f)**
**Lit. Ref.**	J. Yu, C. J. Li, H. Zeng, *Angew. Chem. Int. Ed.* **2021**, *60*, 4043–4048.

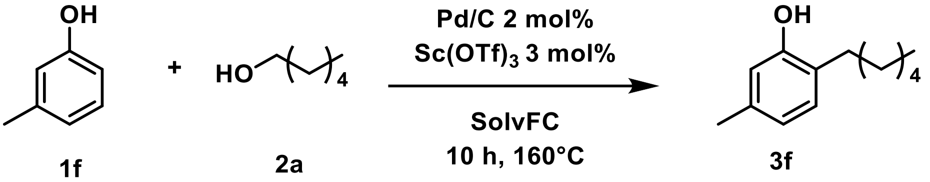

METHOD:

In a 12 mL screw-cap vial equipped with a magnetic stir bar, 8.5 mg of Pd/C (2 mol%), 5.9 mg of $Sc(OTf)_3$ (3 mol%) and 41.8 mg of 3-methylphenol (*m*-cresol) (0.4 mmol) are added. Then, 1 mL of 1-hexanol (20 eq) is added, and the mixture is kept stirring at 160°C for 10 h. Once the reaction is finished, the mixture is cooled to room temperature; Pd/C and $Sc(OTf)_3$ are filtered with EtOAc on a pad of silica gel. The excess of alcohol is separated from the filtrate by distillation and the residue is purified by chromatographic column using 95:5 ETP:EtOAc as the eluent. The purified product is obtained as yellow oil (60% isolated yield, 49 mg).

**Mol Formula**		$C_{13}H_{20}O$	**m.p.**	Yellow oil
	**δ value**	**No. H**	**Mult.**	**J value/Hz**
	6.99	1	*d*	7.5
	6.69	1	*d*	7.4
**^{1}H NMR**	6.59	1	*s*	
**400 MHz**	4.59	1	*s*	
**$CDCl_3$**	2.56	2	*t*	7.5
	2.27	3	*s*	
	1.64-1.53	2	*m*	
	1.42-1.24	6	*m*	
	0.94-0.83	3	*m*	

^{13}C NMR (100.6 MHz, $CDCl_3$) δ: 153.2, 136.9, 130.0, 125.4, 121.5, 115.9, 31.8, 29.9, 29.6, 29.2, 22.6, 21.0, 14.1.

GC-EIMS (m/z, %): 192 (M+, 28), 122 (15), 121 (100),91 (23), 77 (19).

Table S13. Spectral data of 2-hexyl-3,5-dimethylphenol (**3g**).

**Chem. Name**	**2-hexyl-3,5-dimethylphenol (3g)**
**Lit. Ref.**	*none*

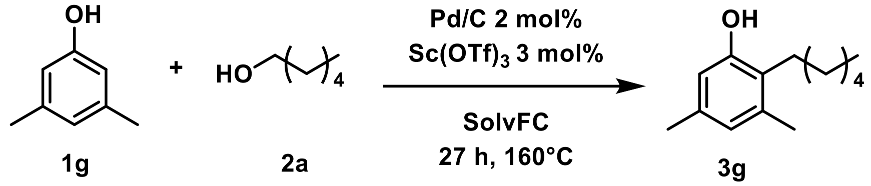

METHOD:

In a 12 mL screw-cap vial equipped with a magnetic stir bar, 8.5 mg of Pd/C (2 mol%), 5.9 mg of $Sc(OTf)_3$ (3 mol%) and 48.8 mg of 3,5-dimethylphenol (0.4 mmol) are added. Then, 1 mL of 1-hexanol (20 eq) is added, and the mixture is kept stirring at 160°C for 27 h. Once the reaction is finished, the mixture is cooled to room temperature; Pd/C and $Sc(OTf)_3$ are filtered with EtOAc on a pad of silica gel. The excess of alcohol is separated from the filtrate by distillation and the residue is purified by chromatographic column using 95:5 ETP:EtOAc as the eluent. The purified product is obtained as pale-yellow oil (62% isolated yield, 49 mg).

**Mol Formula**		$C_{14}H_{22}O$	**m.p.**	Pale-yellow oil
	**δ value**	**No. H**	**Mult.**	**J value/Hz**
	6.58	1	*s*	
	6.45	1	*s*	
**^{1}H NMR 400 MHz $CDCl_3$**	4.52	1	*s*	
	2.57	2	*t*	7.8
	2.26	3	*s*	
	2.23	3	*s*	
	1.53-1.45	2	*m*	
	1.33-1.31	6	*m*	
	0.91-0.87	3	*m*	

^{13}C NMR (100.6 MHz, $CDCl_3$) δ: 153.5, 137.6, 136.1, 124.4, 123.7, 113.7, 31.9, 29.8, 29.4, 26.2, 22.8, 21.0, 19.5, 14.2.

GC-EIMS (m/z, %): 207 (20), 206 (M+,100),136 (63), 135 (100), 105 (20), 91 (65), 79 (21), 77 (17).

Table S14. Spectral data of 2-hexyl-4-ethylphenol (**3h**).

**Chem. Name**	**2-hexyl-4-ethylphenol (3h)**
**Lit. Ref.**	*none*

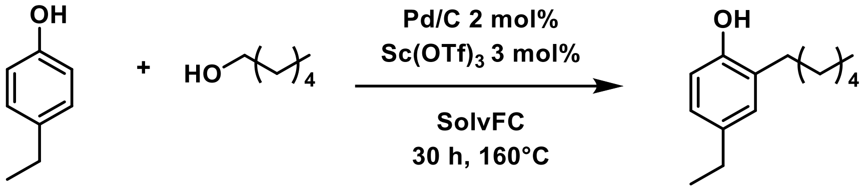

1h 2a 3h

METHOD:

In a 12 mL screw-cap vial equipped with a magnetic stir bar, 8.5 mg of Pd/C (2 mol%), 5.9 mg of $Sc(OTf)_3$ (3 mol%) and 48.8 mg of 4-ethylphenol (0.4 mmol) are added. Then, 1 mL of 1-hexanol (20 eq) is added, and the mixture is kept stirring at 160°C for 30 h. Once the reaction is finished, the mixture is cooled to room temperature; Pd/C and $Sc(OTf)_3$ are filtered with EtOAc on a pad of silica gel. The excess of alcohol is separated from the filtrate by distillation and the residue is purified by chromatographic column using 95:5 ETP:EtOAc as the eluent. The purified product is obtained as yellow oil (61% isolated yield, 50 mg).

**Mol Formula**		$C_{14}H_{22}O$	**m.p.**	Yellow oil
	**δ value**	**No. H**	**Mult.**	**J value/Hz**
	6.95	1	*s*	
	6.92-6.89	1	*m*	
**^{1}H NMR**	6.70-6.68	1	*d*	8.0
**400 MHz**	4.55	1	*s*	
**$CDCl_3$**	2.60-2.54	4	*m*	
	1.63-1.57	2	*m*	
	1.38-1.30	6	*m*	
	1.21	3	*t*	7.6
	0.90	3	*t*	6.8

^{13}C NMR (100.6 MHz, $CDCl_3$) δ: 151.3, 136.5, 129.6, 128.4, 126.1, 115.0, 31.8, 30.1, 29.1, 29.3, 28.1, 22.6, 15.9, 14.1

GC-EIMS (m/z, %): 207 (36), 206 (M+,100), 192 (18), 191 (100), 136 (62), 135 (100), 133 (19) 121 (51), 107 (50), 105 (17), 91 (68), 79 (19), 77 (25).

Table S15. Spectral data of 2-hexyl-4-ethylphenol (**3i**).

**Chem. Name**	**2-hexyl-4-isopropylphenol (3i)**
**Lit. Ref.**	*none*

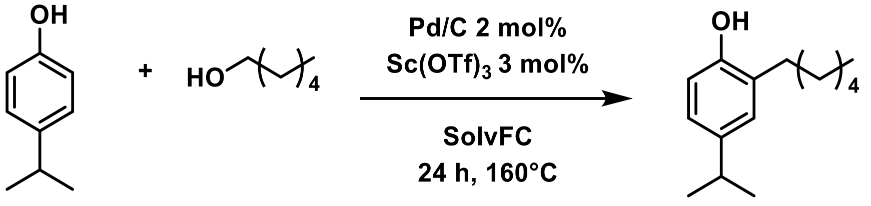

1i 2a 3i

METHOD:

In a 12 mL screw-cap vial equipped with a magnetic stir bar, 8.5 mg of Pd/C (2 mol%), 5.9 mg of $Sc(OTf)_3$ (3 mol%) and 54.4 mg of 4-isopropylphenol (0.4 mmol) are added. Then, 1 mL of 1-hexanol (20 eq) is added, and the mixture is kept stirring at 160°C for 24 h. Once the reaction is finished, the mixture is cooled to room temperature; Pd/C and $Sc(OTf)_3$ are filtered with EtOAc on a pad of silica gel. The excess of alcohol is separated from the filtrate by distillation and the residue is purified by chromatographic column using a variable ratio eluent mixture of ETP and EtOAc. The purified product is obtained as yellow oil (62% isolated yield, 54 mg).

**Mol Formula**	$C_{15}H_{24}O$	**m.p.**	Yellow oil	
	**δ value**	**No. H**	**Mult.**	**J value/Hz**
**^{1}H NMR 400 MHz $CDCl_3$**	6.97-6.96	1	*m*	
	6.94-6.92	1	*m*	
	6.70-6.68	1	*m*	
	4.53	1	*s*	
	2.83	1	*septet*	6.9
	2.58	2	*t*	7.8
	1.65-1.57	2	*m*	
	1.38-1.31	6	*m*	
	1.22	6	*d*	6.9
	0.91-0.88	3	*t*	6.8

^{13}C NMR (100.6 MHz, $CDCl_3$) δ: 151.3, 141.2, 128.2, 128.2, 124.6, 115.0, 33.3 31.7, 30.2, 29.9, 29.3, 24.3, 22.6, 14.1

GC-EIMS (m/z, %): 221 (25), 220 (M+, 94), 206 (82), 205 (100), 150 (16), 149 (90), 147 (17) 135 (63), 134 (24), 133 (33), 121 (34), 107 (40), 113 (31), 91 (41), 79 (18), 77 (24), 43 (36), 41 (20).

**Table S16.** Spectral data of 2-hexyl-4-tertbutylphenol (**3j**).	
**Chem. Name**	**2-hexyl-4-*tert*-butylphenol (3j)**
**Lit. Ref.**	H. Zeng, J. Yu, C. J. Li, *Chem. Commun.,* **2020**, *56*, 1239-1242.

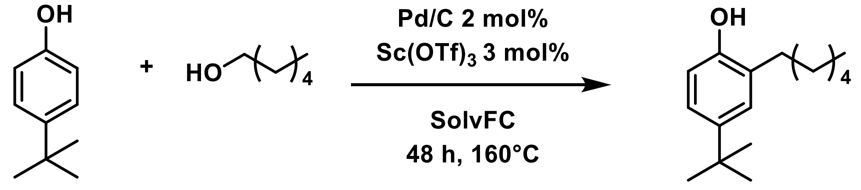

1j **2a** **3j**

METHOD:

In a 12 mL screw-cap vial equipped with a magnetic stir bar, 8.5 mg of Pd/C (2 mol%), 5.9 mg of $Sc(OTf)_3$ (3 mol%) and 60.0 mg of 4-*tert*-butylphenol (0.4 mmol) are added. Then, 1 mL of 1-hexanol (20 eq) is added, and the mixture is kept stirring at 160°C for 48 h. Once the reaction is finished, the mixture is cooled to room temperature; Pd/C and $Sc(OTf)_3$ are filtered with EtOAc on a pad of silica gel. The excess of alcohol is separated from the filtrate by distillation and the residue is purified by chromatographic column using 95:5 ETP:EtOAc as the eluent. The purified product is obtained as yellow oil (61% isolated yield, 55 mg).

**Mol Formula**		$C_{16}H_{26}O$	**m.p.**	Yellow oil
	**δ value**	**No. H**	**Mult.**	**J value/Hz**
**^{1}H NMR 400 MHz $CDCl_3$**	7.12-7.11	1	*m*	
	7.10-7.07	1	*m*	
	6.69	1	*d*	8.2
	4.49	1	*s*	
	2.60-2.56	2	*m*	
	1.64-1.55	2	*m*	
	1.33-1.30	6	*m*	
	1.29	9	*s*	
	0.91-0.87	3	*m*	

^{13}C NMR (100.6 MHz, $CDCl_3$) δ: 151.1, 143.4, 127.8, 127.2, 123.7, 114.7, 31.7, 31.6, 30.4, 29.9, 29.7, 29.3, 22.7, 14.1

GC-EIMS (m/z, %): 235 (20), 234 (M+, 100), 220 (100), 219 (100), 163 (35), 148 (20), 147 (19), 133 (22), 121 (15), 107 (22), 105 (18), 91 (24), 77 (17), 57 (18)

Table S17. Spectral data of 2-pentyl-3,5-dimethoxyphenol (**3l**).

**Chem. Name**	**2-pentyl-3,5-dimethoxyphenol (3l)**
**Lit. Ref.**	F. Roelens, K. Huvaere, W. Dhooge, M. Van Cleemput, F. Comhaire, D. De Keukeleire, *Eur J Med Chem* **2005**, *40*, 1042–1051.

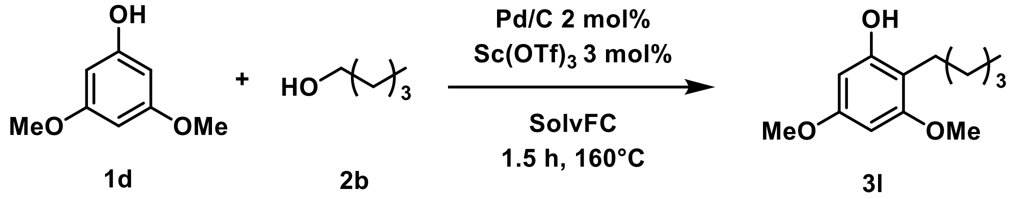

METHOD:

In a 12 mL screw-cap vial equipped with a magnetic stir bar, 8.5 mg of Pd/C (2 mol%), 5.9 mg of $Sc(OTf)_3$ (3 mol%) and 61.6 mg of 3,5-dimethoxyphenol (0.4 mmol) are added. Then, 0.87 mL of 1-pentanol (20 eq) is added, and the mixture is kept stirring at 160°C for 1.5 h. Once the reaction is finished, the mixture is cooled to room temperature; Pd/C and $Sc(OTf)_3$ are filtered with EtOAc on a pad of silica gel. The excess of alcohol is separated from the filtrate by distillation and the residue is purified by TLC preparative with 95:5 ETP:EtOAc as eluent. The purified product is obtained as orange oil (58% isolated yield, 55 mg).

**Mol Formula**		$C_{13}H_{20}O_3$	**m.p.**	Orange oil
	**δ value**	**No. H**	**Mult.**	**J value/Hz**
**^{1}H NMR 400 MHz $CDCl_3$**	6.07	1	*s*	
	6.04	1	*s*	
	4.88	1	*s*	
	3.77	3	*s*	
	3.74	3	*s*	
	2.52	2	*t*	7.4
	1.51-1.41	2	*m*	
	1.37-1.26	4	*m*	
	0.90-0.87	3	*m*	

^{13}C NMR (100.6 MHz, $CDCl_3$) δ: 159.3, 159.0, 154.8, 109.6, 93.4, 91.5, 55.7, 55.4, 32.0, 29.3, 22.74, 22.73, 14.2.

GC-EIMS (m/z, %): 224 (M+, 39), 168 (23), 167 (100), 137 (26), 109 (20).

**Table S18.** Spectral data of 2-heptyl-3,5-dimethoxyphenol (**3m**).	
**Chem. Name**	**2-heptyl-3,5-dimethoxyphenol (3m)**
**Lit. Ref.**	F. Roelens, K. Huvaere, W. Dhooge, M. Van Cleemput, F. Comhaire, D. De Keukeleire, *Eur J Med Chem* **2005**, *40*, 1042–1051.

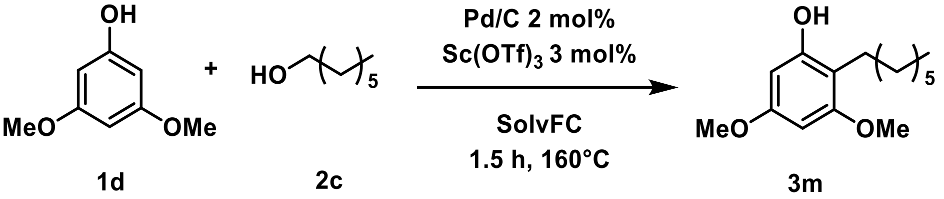

METHOD:

In a 12 mL screw-cap vial equipped with a magnetic stir bar, 8.5 mg of Pd/C (2 mol%), 5.9 mg of $Sc(OTf)_3$ (3 mol%) and 61.6 mg of 3,5-dimethoxyphenol (0.4 mmol) are added. Then, 1.1 mL of 1-eptanol (20 eq) is added, and the mixture is kept stirring at 160°C for 1.5 h. Once the reaction is finished, the mixture is cooled to room temperature; Pd/C and $Sc(OTf)_3$ are filtered with EtOAc on a pad of silica gel. The excess of alcohol is separated from the filtrate by distillation and the residue is purified by TLC preparative with 93:7 ETP:EtOAc as the eluent. The purified product is obtained as orange oil (66% isolated yield, 60 mg).

**Mol Formula**		$C_{15}H_{24}O_3$	**m.p.**	Orange oil
**^{1}H NMR 400 MHz $CDCl_3$**	**δ value**	**No. H**	**Mult.**	**j value/Hz**
	6.09	1	*s*	
	6.04	1	*s*	
	4.88	1	*s*	
	3.77	3	*s*	
	3.75	3	*s*	
	2.53	2	*t*	7.5
	1.52-1.42	2	*m*	
	1.38-1.23	8	*m*	
	0.88-0.86	3	*m*	

^{13}C NMR (100.6 MHz, $CDCl_3$) δ: 159.1, 158.9, 154.7, 109.5, 93.3, 91.4, 55.6, 55.3, 31.9, 29.7, 29.5, 29.3, 22.7, 22.7, 14.1.

GC-EIMS (m/z, %): 252 (M+, 25), 168 (19), 167 (100), 137 (16).

Table S19. Spectral data of 2-octyl-3,5-dimethoxyphenol (**3n**).

**Chem. Name**	**2-octyl-3,5-dimethoxyphenol (3n)**
**Lit. Ref.**	G. Brufani, F. Valentini, F. Sabatelli, B. Di Erasmo, A. M. Afanasenko, C. J. Li, L. Vaccaro, *Green Chem.* **2022**, *24*, 9094.

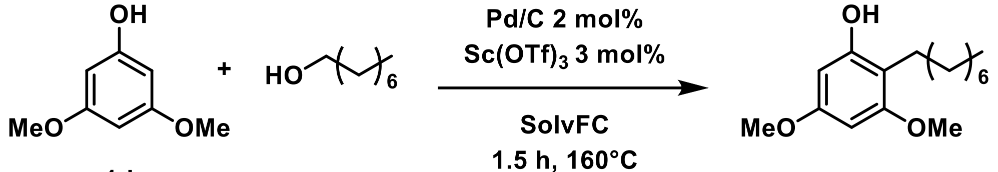

1d 2d 3n

METHOD:

In a 12 mL screw-cap vial equipped with a magnetic stir bar, 8.5 mg of Pd/C (2 mol%), 5.9 mg of $Sc(OTf)_3$ (3 mol%) and 61.6 mg of 3,5-dimethoxyphenol (0.4 mmol) are added. Then, 1.3 mL of 1-octanol (20 eq) is added, and the mixture is kept stirring at 160°C for 1.5 h. Once the reaction is finished, the mixture is cooled to room temperature; Pd/C and $Sc(OTf)_3$ are filtered with EtOAc on a pad of silica gel. The excess of alcohol is separated from the filtrate by distillation and the residue is purified by TLC preparative with 9:1 ETP:EtOAc as the eluent. The purified product is obtained as orange oil (63% isolated yield, 58 mg).

**Mol Formula**		$C_{16}H_{26}O_3$	**m.p.**	Orange oil
	**δ value**	**No. H**	**Mult.**	**j value/Hz**
^{1}**H NMR 400 MHz CDCl$_3$**	6.09	1	*s*	
	6.04	1	*s*	
	4.81	1	*s*	
	3.77	3	*s*	
	3.76	3	*s*	
	2.53	2	*t*	7.4
	1.52-1.42	2	*m*	
	1.38-1.21	10	*m*	
	0.92-0.85	3	*m*	

^{13}**C NMR (100.6 MHz, CDCl$_3$) δ:** 159.1, 158.9, 154.7, 109.4, 93.3, 91.4, 55.6, 55.3, 31.9, 29.7, 29.6, 29.5, 29.3, 22.7, 22.7, 14.1.

GC-EIMS (m/z, %): 266 (M+, 12), 167 (100).

Table S20. Spectral data of 3,5-dimethoxy-2-(3,5,5-trimethylhexyl)phenol (**3o**).

**Chem. Name**	**3,5-dimethoxy-2-(3,5,5-trimethylhexyl)phenol (3o)**
**Lit. Ref.**	*none*

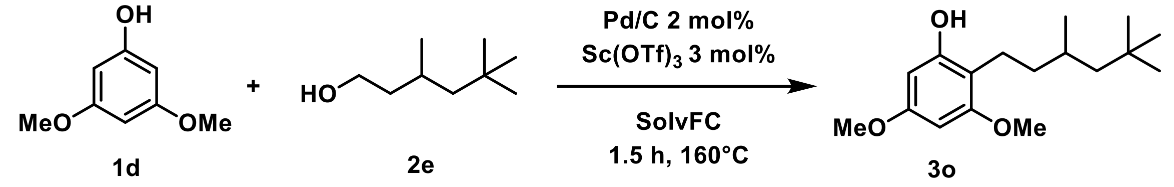

METHOD:

In a 12 mL screw-cap vial equipped with a magnetic stir bar, 8.5 mg of Pd/C (2 mol%), 5.9 mg of $Sc(OTf)_3$ (3 mol%) and 61.6 mg of 3,5-dimethoxyphenol (0.4 mmol) are added. Then, 1.2 mL of 3,5,5-trimethyl-1-hexanol (20 eq) is added, and the mixture is kept stirring at 160°C for 1.5 h. Once the reaction is finished, the mixture is cooled to room temperature; Pd/C and $Sc(OTf)_3$ are filtered with EtOAc on a pad of silica gel. The excess of alcohol is separated from the filtrate by distillation and the residue is purified by TLC preparative with 9:1 ETP:EtOAc as the eluent. The purified product is obtained as yellow oil (67% isolated yield, 75 mg).

**Mol Formula**		$C_{17}H_{28}O_3$	**m.p.**	Yellow oil
**^{1}H NMR 400 MHz $CDCl_3$**	**δ value**	**No. H**	**Mult.**	**j value/Hz**
	6.09-6.08	1	*m*	
	6.03-6.02	1	*m*	
	4.75-4.68	1	*m*	
	3.77	3	*s*	
	3.75	3	*s*	
	2.53-2.50	2	*m*	
	1.56-1.40	2	*m*	
	1.33-1.26	2	*m*	
	1.09-1.04	1	*m*	
	0.99	3	*d*	6.6
	0.89	9	*s*	

^{13}C NMR (100.6 MHz, $CDCl_3$) δ: 159.1, 158.9, 154.5, 109.7, 93.3, 91.4, 55.6, 55.3, 51.1, 39.0, 31.1, 30.0, 29.7, 22.6, 20.5

GC-EIMS (m/z, %): 280 (M+, 18), 168 (11), 167 (100)

Table S21. Spectral data of 2-isopentyl-3,5-dimethoxyphenol (**3p**).

**Chem. Name**	**2-isopentyl-3,5-dimethoxyphenol (3p)**
**Lit. Ref.**	G. Brufani, F. Valentini, F. Sabatelli, B. Di Erasmo, A. M. Afanasenko, C. J. Li, L. Vaccaro, *Green Chem.* **2022**, *24*, 9094.

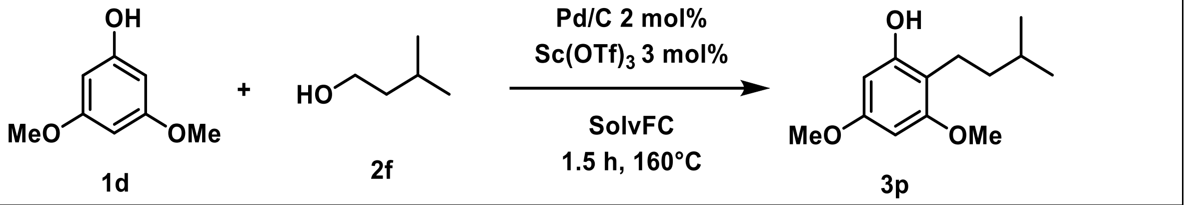

METHOD:

In a 12 mL screw-cap vial equipped with a magnetic stir bar, 8.5 mg of Pd/C (2 mol%), 5.9 mg of $Sc(OTf)_3$ (3 mol%) and 61.6 mg of 3,5-dimethoxyphenol (0.4 mmol) are added. Then, 0.87 mL of isoamyl alcohol (20 eq) is added, and the mixture is kept stirring at 160°C for 1.5 h. Once the reaction is finished, the mixture is cooled to room temperature; Pd/C and $Sc(OTf)_3$ are filtered with EtOAc on a pad of silica gel. The excess of alcohol is separated from the filtrate by distillation and the residue is purified by TLC preparative with 9:1 ETP:EtOAc as the eluent. The purified product is obtained as yellow oil (72% isolated yield, 64 mg).

**Mol Formula**		$C_{13}H_{20}O_3$	**m.p.**	Yellow oil
	**δ value**	**No. H**	**Mult.**	**j value/Hz**
**^{1}H NMR 400 MHz $CDCl_3$**	6.09-6.08	1	*m*	
	6.04-6.03	1	*m*	
	4.73	1	*s*	
	3.77	3	*s*	
	3.75	3	*s*	
	2.55-2.51	2	*m*	
	1.59-1.56	1	*m*	
	1.38-1.32	2	*m*	
	0.94	6	*d*	6.6

^{13}C NMR (100.6 MHz, $CDCl_3$) δ: 159.1, 158.9, 154.6, 109.6, 93.3, 91.4, 55.6, 55.3, 38.6, 28.2, 22.6, 20.5

GC-EIMS (m/z, %): 224 (M+, 24), 168 (11), 167 (100), 137 (13)

Table S22. Spectral data of 2-pentyl-3,4,5-dimethoxyphenol (**3q**).

**Chem. Name**	**2-pentyl-3,4,5-trimethoxyphenol (3q)**
**Lit. Ref.**	*none*

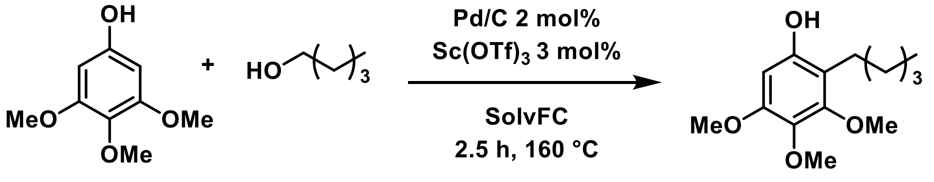

1e **2b** **3q**

METHOD:

In a 12 mL screw-cap vial equipped with a magnetic stir bar, 8.5 mg of Pd/C (2 mol%), 5.9 mg of $Sc(OTf)_3$ (3 mol%) and 73.6 mg of 3,4,5-dimethoxyphenol (0.4 mmol) are added. Then, 0.87 mL of 1-pentanol (20 eq) is added, and the mixture is kept stirring at 160°C for 2.5 h. Once the reaction is finished, the mixture is cooled to room temperature; Pd/C and $Sc(OTf)_3$ are filtered with EtOAc on a pad of silica gel. The excess of alcohol is separated from the filtrate by distillation and the residue is purified by chromatographic column using 95:5 ETP:EtOAc as the eluent. The purified product is obtained as red oil (75% isolated yield, 76 mg).

**Mol Formula**		$C_{14}H_{22}O_4$	**m.p.**	Red oil
	**δ value**	**No. H**	**Mult.**	**j value/Hz**
**^{1}H NMR 400 MHz $CDCl_3$**	6.21	1	*s*	
	4.52	1	*s*	
	3.88	3	*s*	
	3.80	6	*s*	
	2.53	2	*t*	7.8
	1.52-1.49	2	*m*	
	1.37-1.33	4	*m*	
	0.90	3	*t*	7.0

^{13}C NMR (100.6 MHz, $CDCl_3$) δ: 152.5, 151.8, 149.7, 136.5, 114.4, 96.0, 61.3, 61.0, 56.1, 32.1, 30.0, 23.7, 22.7, 14.2.

GC-EIMS (m/z, %): 255 (34), 254 (M+, 100), 240 (16), 239 (98), 198 (40), 197 (100), 183 (24), 182 (100), 167 (16), 152 (19), 139 (32), 69 (18)

**Table S23.** Spectral data of 2-heptyl-3,4,5-dimethoxyphenol (**3r**).	
**Chem. Name**	**2-heptyl-3,4,5-trimethoxyphenol (3r)**
**Lit. Ref.**	*none*

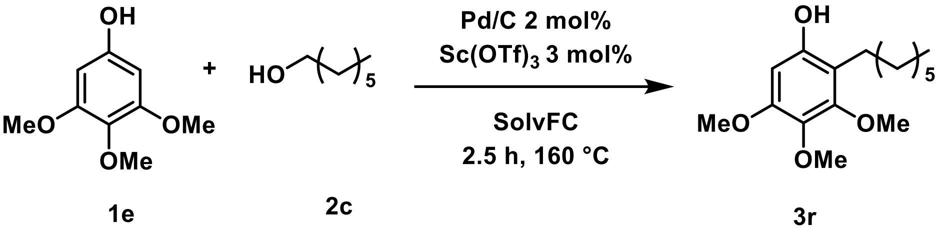

METHOD:

In a 12 mL screw-cap vial equipped with a magnetic stir bar, 8.5 mg of Pd/C (2 mol%), 5.9 mg of $Sc(OTf)_3$ (3 mol%) and 73.6 mg of 3,4,5-trimethoxyphenol (0.4 mmol) are added. Then, 0.87 mL of 1-heptanol (20 eq) is added, and the mixture is kept stirring at 160°C for 2.5 h. Once the reaction is finished, the mixture is cooled to room temperature; Pd/C and $Sc(OTf)_3$ are filtered with EtOAc on a pad of silica gel. The excess of alcohol is separated from the filtrate by distillation and the residue is purified by chromatographic column using a variable ratio eluent mixture of ETP and EtOAc. The purified product is obtained as red oil (70% isolated yield, 79 mg).

**Mol Formula**		$C_{16}H_{26}O_4$	**m.p.**	Red oil
	**δ value**	**No. H**	**Mult.**	**j value/Hz**
**^{1}H NMR 400 MHz $CDCl_3$**	6.20	1	*s*	
	4.80	1	*s*	
	3.87	3	*s*	
	3.80	3	*s*	
	3.78	3	*s*	
	2.54-2.51	2	*m*	
	1.53-1.46	2	*m*	
	1.33-1.28	8	*m*	
	0.89-0.86	3	*m*	

^{13}C NMR (100.6 MHz, $CDCl_3$) δ: 152.5, 151.7, 149.8, 136.3, 114.5, 96.0, 61.3, 61.0, 56.0, 32.0, 30.3, 29.9, 29.3, 23.8, 22.8, 14.2.

GC-EIMS (m/z, %): 283 (40), 282 (M+, 100), 268 (17), 267 (92), 198 (39), 197 (100), 183 (21), 182 (100), 152 (15), 139 (25).

Table S24. Spectral data of 2-octyl-3,4,5-dimethoxyphenol (**3s**).

**Chem. Name**	**2-octyl-3,4,5-trimethoxyphenol (3s)**
**Lit. Ref.**	*none*

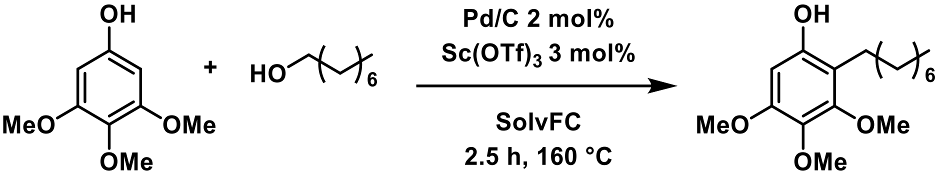

1e **2d** **3s**

METHOD:

In a 12 mL screw-cap vial equipped with a magnetic stir bar, 8.5 mg of Pd/C (2 mol%), 5.9 mg of $Sc(OTf)_3$ (3 mol%) and 73.6 mg of 3,4,5-trimethoxyphenol (0.4 mmol) are added. Then, 1.3 mL of 1-octanol (20 eq) is added, and the mixture is kept stirring at 160°C for 2.5 h. Once the reaction is finished, the mixture is cooled to room temperature; Pd/C and $Sc(OTf)_3$ are filtered with EtOAc on a pad of silica gel. The excess of alcohol is separated from the filtrate by distillation and the residue is purified by chromatographic column using a variable ratio eluent mixture of ETP and EtOAc. The purified product is obtained as red oil (60% isolated yield, 67 mg).

**Mol Formula**		$C_{17}H_{28}O_4$	**m.p.**	Red oil
	**δ value**	**No. H**	**Mult.**	**j value/Hz**
**¹H NMR 400 MHz $CDCl_3$**	6.19	1	*s*	
	5.18	1	*s*	
	3.87	3	*s*	
	3.79	3	*s*	
	3.74	3	*s*	
	2.53	2	*t*	7.6
	1.53-1.46	2	*m*	
	1.39-1.24	10	*m*	
	0.88-0.85	3	*m*	

¹³C NMR (100.6 MHz, $CDCl_3$) δ: 152.5, 151.6, 150.0, 136.2, 114.7, 96.1, 61.2, 61.0, 55.9, 32.0, 30.3, 30.0, 29.6, 29.4, 23.7, 22.8, 14.2.

GC-EIMS (m/z, %): 297 (39), 296 (M+, 100), 282 (15), 281 (78), 198 (37), 197 (100), 183 (19), 182 (88), 139 (21).

**Table S25.** Spectral data of 2-isopentyl-3,4,5-dimethoxyphenol (**3t**).				
**Chem. Name**	**2-isopentyl-3,5-dimethoxyphenol (3t)**			
**Lit. Ref.**	G. Brufani, F. Valentini, F. Sabatelli, B. Di Erasmo, A. M. Afanasenko, C. J. Li, L. Vaccaro, *Green Chem.* **2022**, *24*, 9094.			

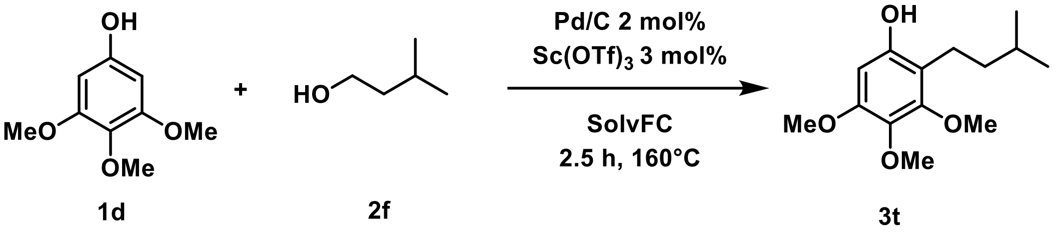

METHOD:

In a 12 mL screw-cap vial equipped with a magnetic stir bar, 8.5 mg of Pd/C (2 mol%), 5.9 mg of $Sc(OTf)_3$ (3 mol%) and 73.6 mg of 3,4,5-trimethoxyphenol (0.4 mmol) are added. Then, 0.87 mL of isoamylalcohol (20 eq) is added, and the mixture is kept stirring at 160°C for 2.5 h. Once the reaction is finished, the mixture is cooled to room temperature; Pd/C and $Sc(OTf)_3$ are filtered with EtOAc on a pad of silica gel. The excess of alcohol is separated from the filtrate by distillation and the residue is purified by TLC preparative with 9:1 ETP:EtOAc as the eluent. The purified product is obtained as yellow solid (84% isolated yield, 85 mg).

**Mol Formula**	$C_{14}H_{22}O_4$	**m.p.**	Yellow solid	
	**δ value**	**No. H**	**Mult.**	**j value/Hz**
**^{1}H NMR 400 MHz $CDCl_3$**	6.19	1	*s*	
	4.91	1	*s*	
	3.88	3	*s*	
	3.80	3	*s*	
	3.76	3	*s*	
	2.55-2.51	2	*m*	
	1.66-1.57	1	*m*	
	1.41-1.35	2	*m*	
	0.96	3	*s*	
	0.94	3	*s*	

^{13}C NMR (100.6 MHz, $CDCl_3$) δ: 152.3, 151.5, 149.7, 136.1, 114.8, 96.0, 61.2, 60.5, 55.9, 39.3, 28.4, 22.5, 21.6

GC-EIMS (m/z, %): 255 (M+1, 11), 254 (M+, 69), 239 (26), 198 (11), 197 (100), 183 (9), 182 (40)

Table S26. Spectral data of 2-pentyl-4-methylphenol (**3u**).

**Chem. Name**	**2-pentyl-4-methylphenol (3u)**
**Lit. Ref.**	*Efficient and cost-effective process for the manufacture of amyl m-cresol; patent n. WO2012017204A1*

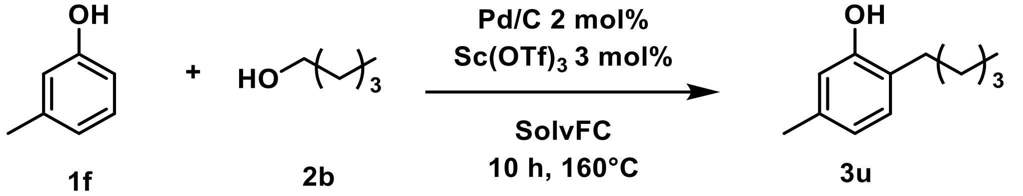

METHOD:

In a 12 mL screw-cap vial equipped with a magnetic stir bar, 8.5 mg of Pd/C (2 mol%), 5.9 mg of $Sc(OTf)_3$ (3 mol%) and 41.8 mg of 3-methylphenol (*m*-cresol) (0.4 mmol) are added. Then, 0.87 mL of 1-pentanol (20 eq) is added, and the mixture is kept stirring at 160°C for 10 h. Once the reaction is finished, the mixture is cooled to room temperature; Pd/C and $Sc(OTf)_3$ are filtered with EtOAc on a pad of silica gel. The excess of alcohol is separated from the filtrate by distillation and the residue is purified by TLC preparative with 96:4 ETP:EtOAc as the eluent. The purified product is obtained as brown solid (43% isolated yield, 30.6 mg).

**Mol Formula**		$C_{12}H_{18}O$	**m.p.**	24°C
	**δ value**	**No. H**	**Mult.**	**J value/Hz**
**^{1}H NMR 400 MHz $CDCl_3$**	7.00	1	*d*	7.6
	6.69	1	*d*	7.6
	6.60	1	*s*	
	4.68	1	*s*	
	2.57	2	*t*	7.5
	2.28	3	*s*	
	1.65-1.57	2	*m*	
	1.39-1.33	4	*m*	
	0.91	3	*t*	6.8

^{13}C NMR (100.6 MHz, $CDCl_3$) δ: 153.3, 137.1, 130.1, 125.6, 121.6, 116.1, 31.8, 29.8, 29.7, 22.7, 21.1, 14.2.

GC-EIMS (m/z, %): 178 (M+, 37), 122 (20), 121 (100),91 (22), 77 (19).

14. ^{1}H-NMR and ^{13}C-NMR spectra of isolated compounds

2-hexyl-4-methoxyphenol

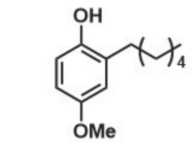

3a

Figure S7. ^{1}H NMR and ^{13}C NMR of 2-hexyl-4-methoxypheno (**3a**)

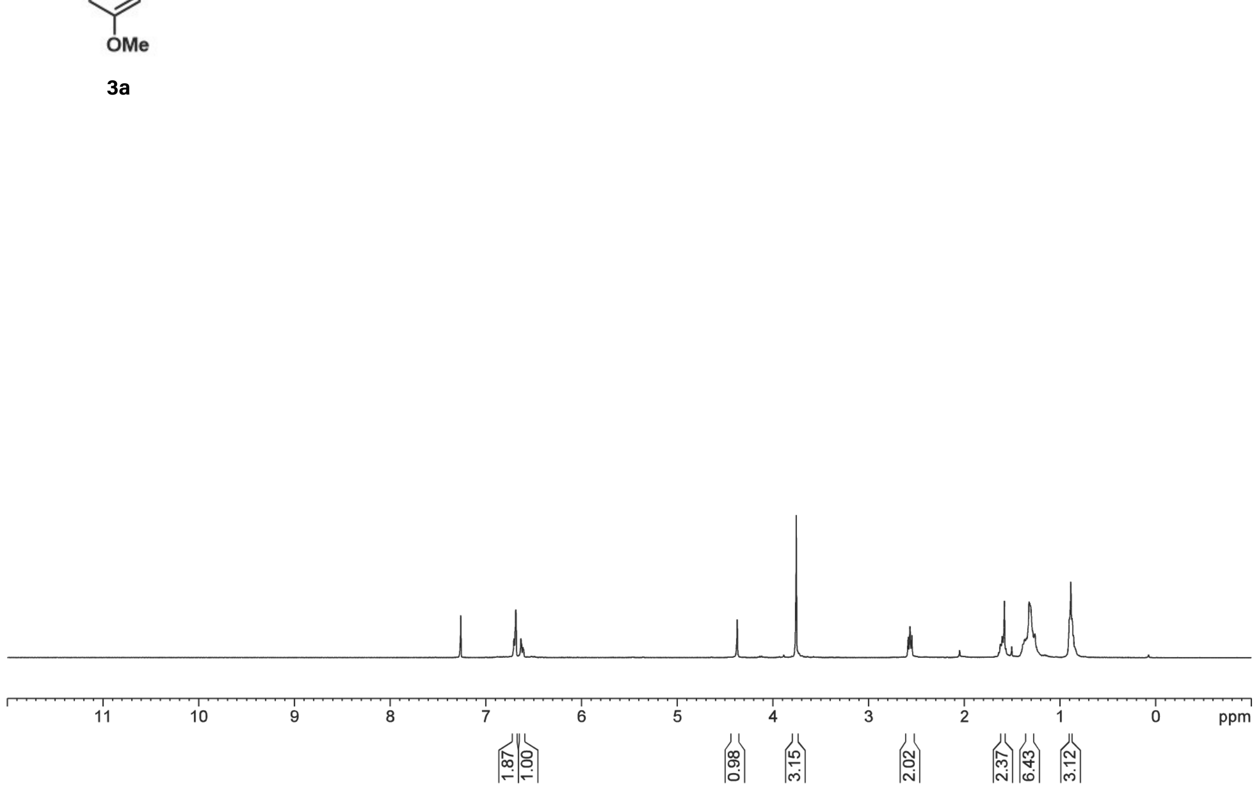

2-hexyl-4-methoxyphenol

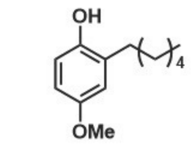

3a

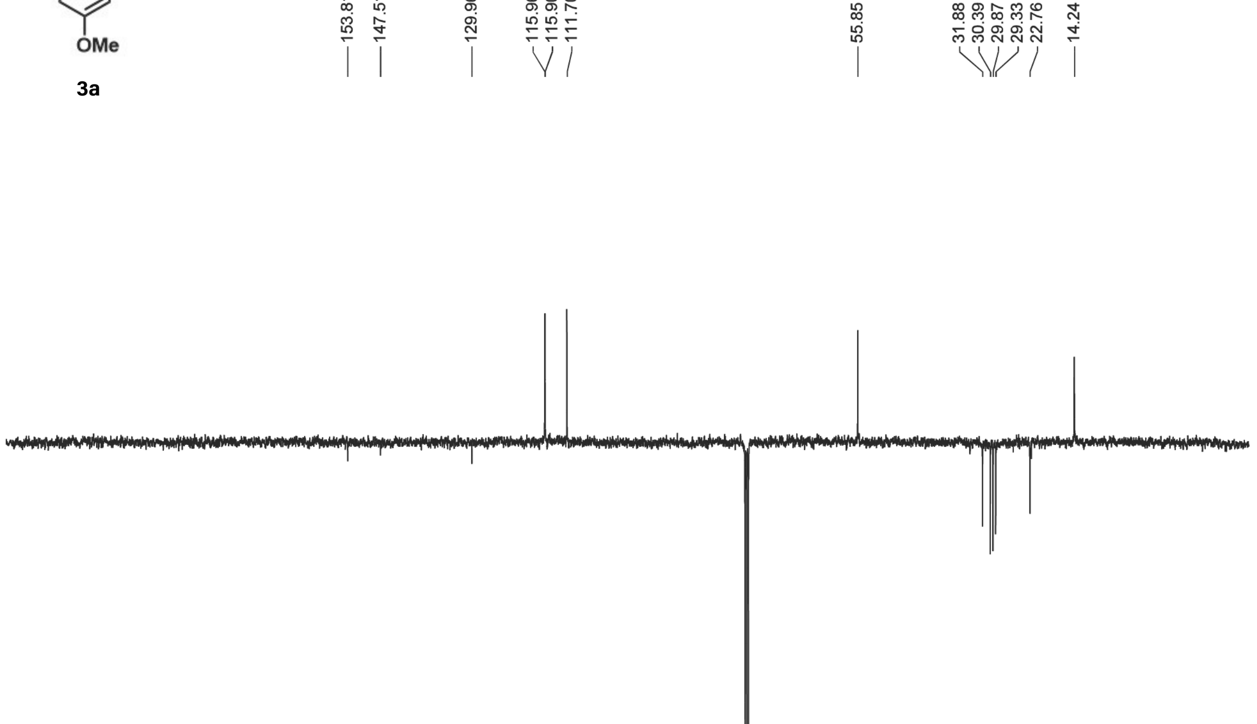

Figure S8. ^{1}H NMR and ^{13}C NMR of 2-hexyl-3-methoxyphenol (**3b**)

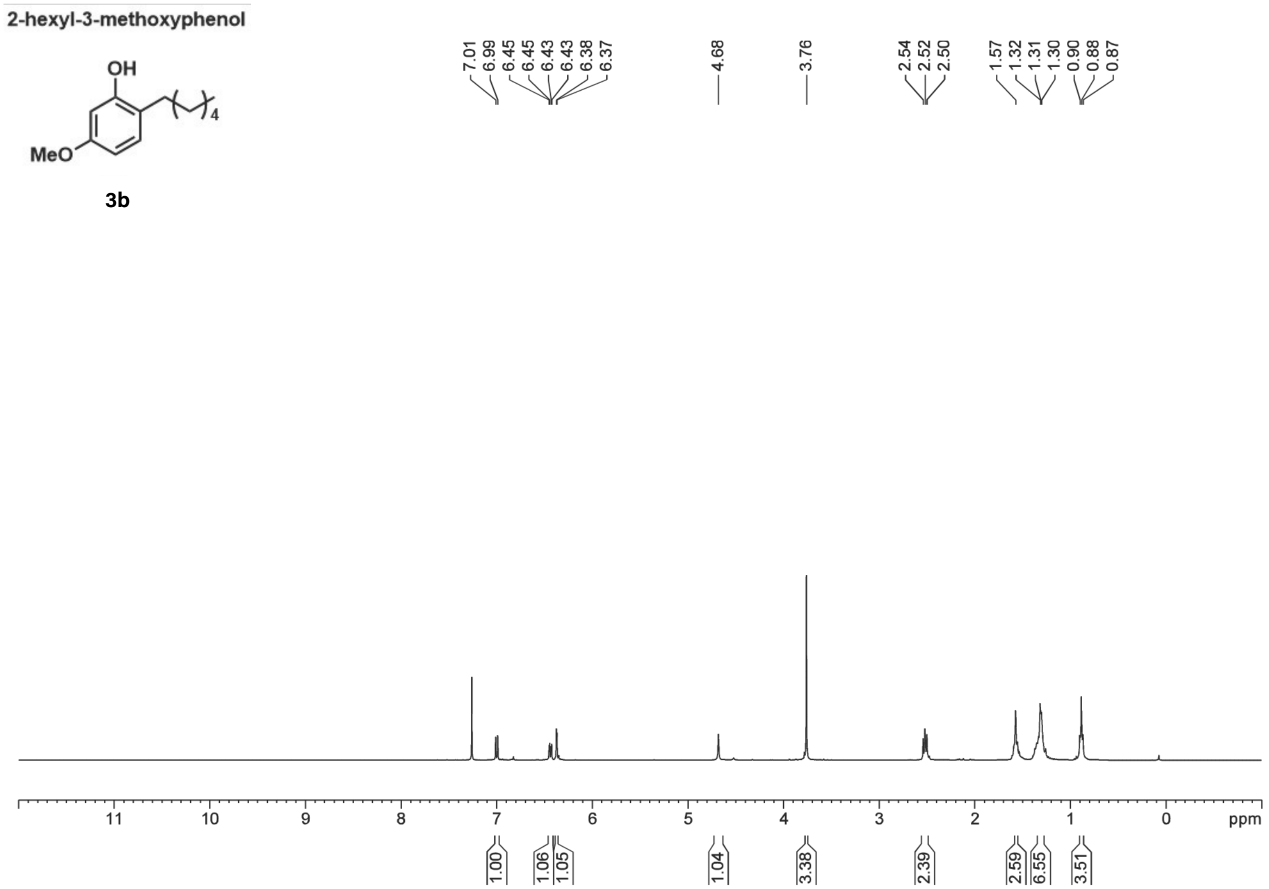

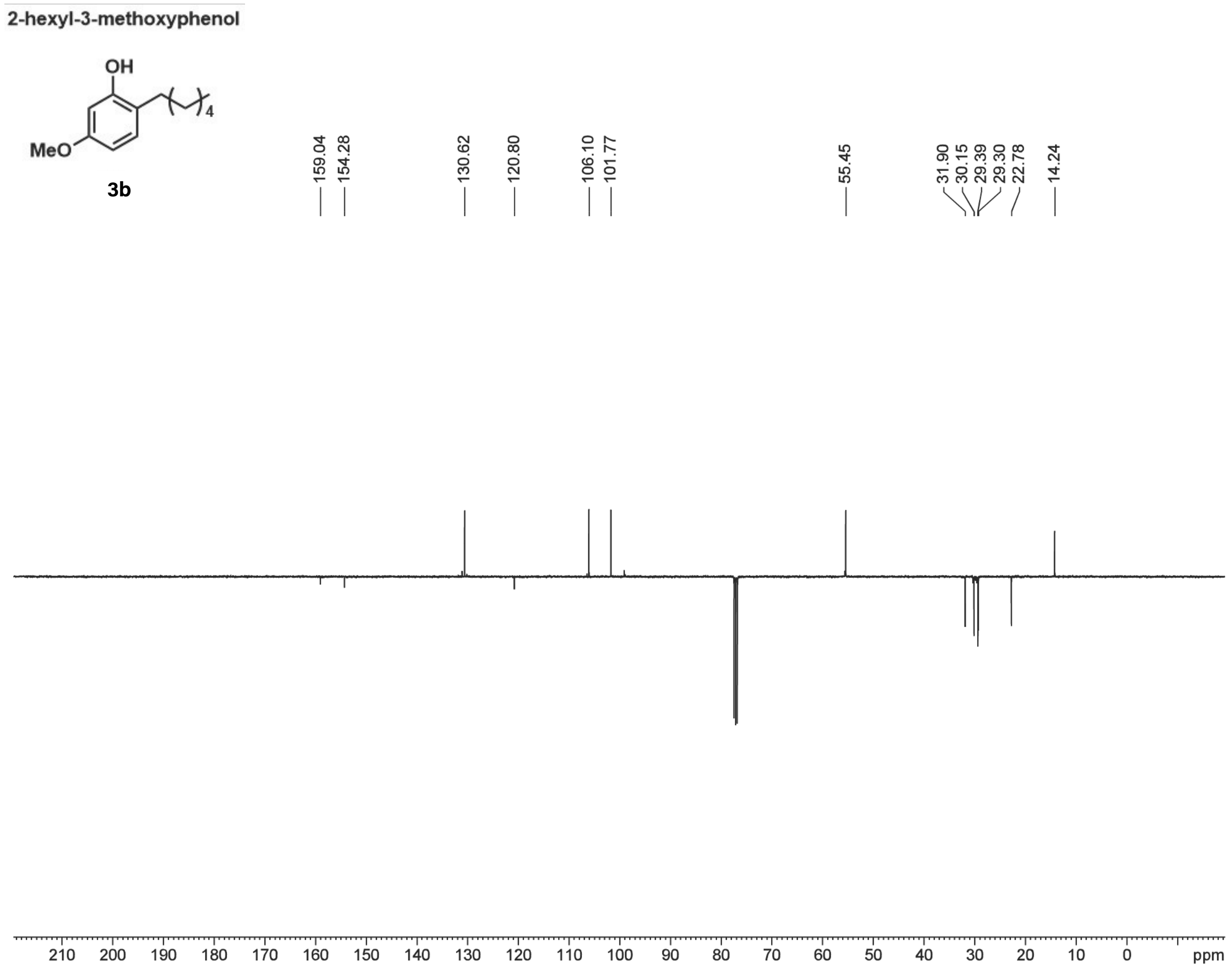

Figure S9. ^{1}H NMR and ^{13}C NMR of 2-hexyl-3,5-dimethoxyphenol (**3c**)

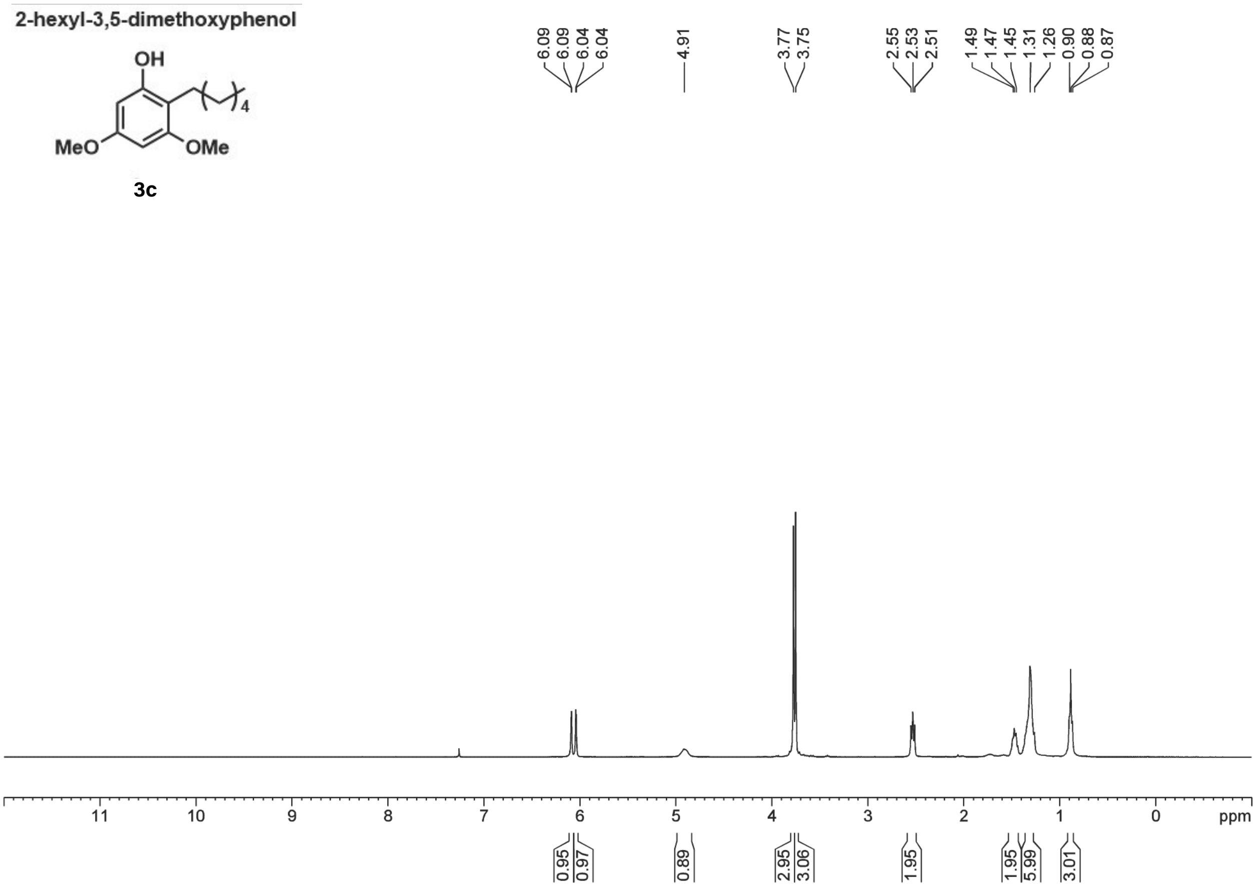

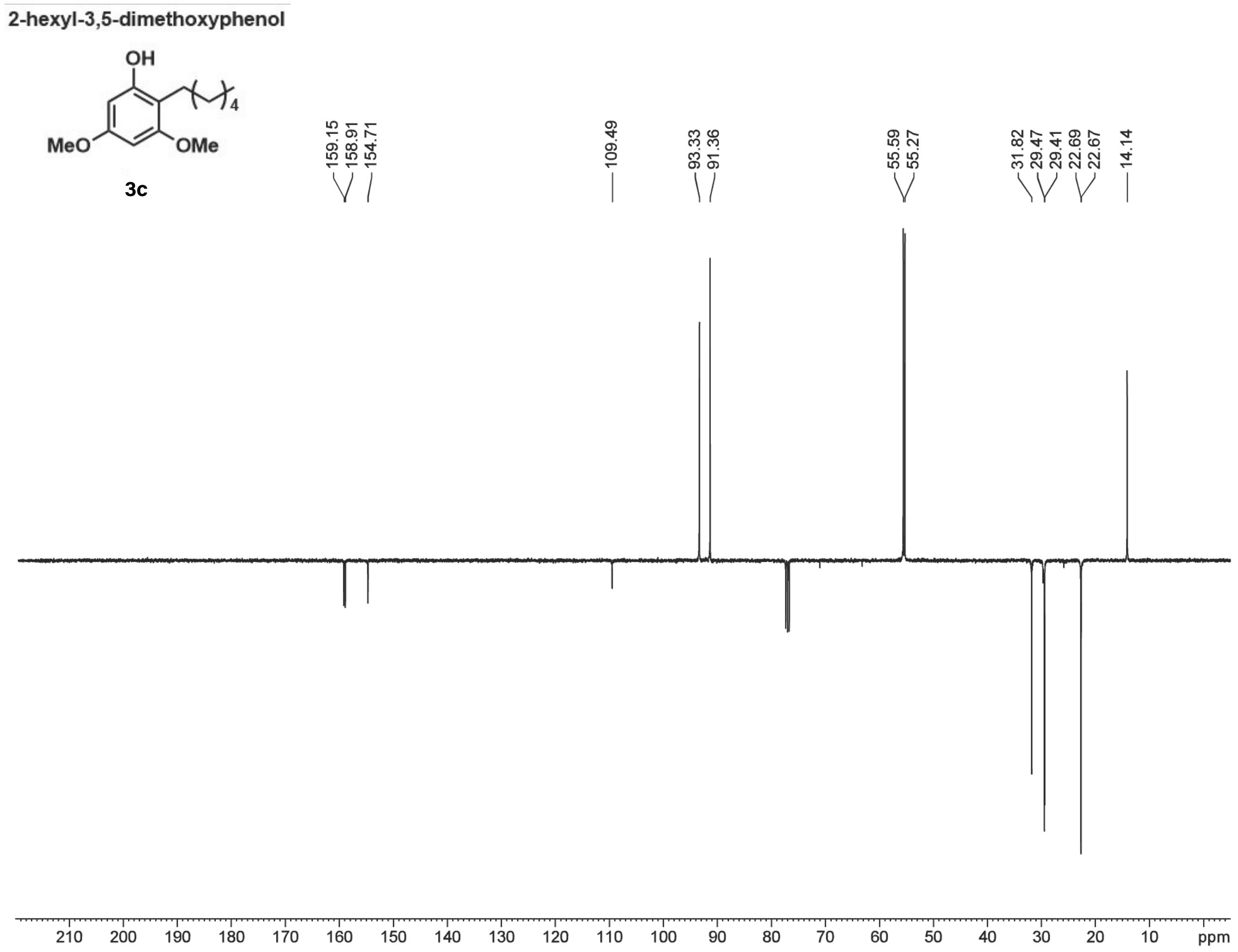

Figure S10. ^{1}H NMR and ^{13}C NMR of 2-hexyl-3,4,5-trimethoxyphenol (**3d**)

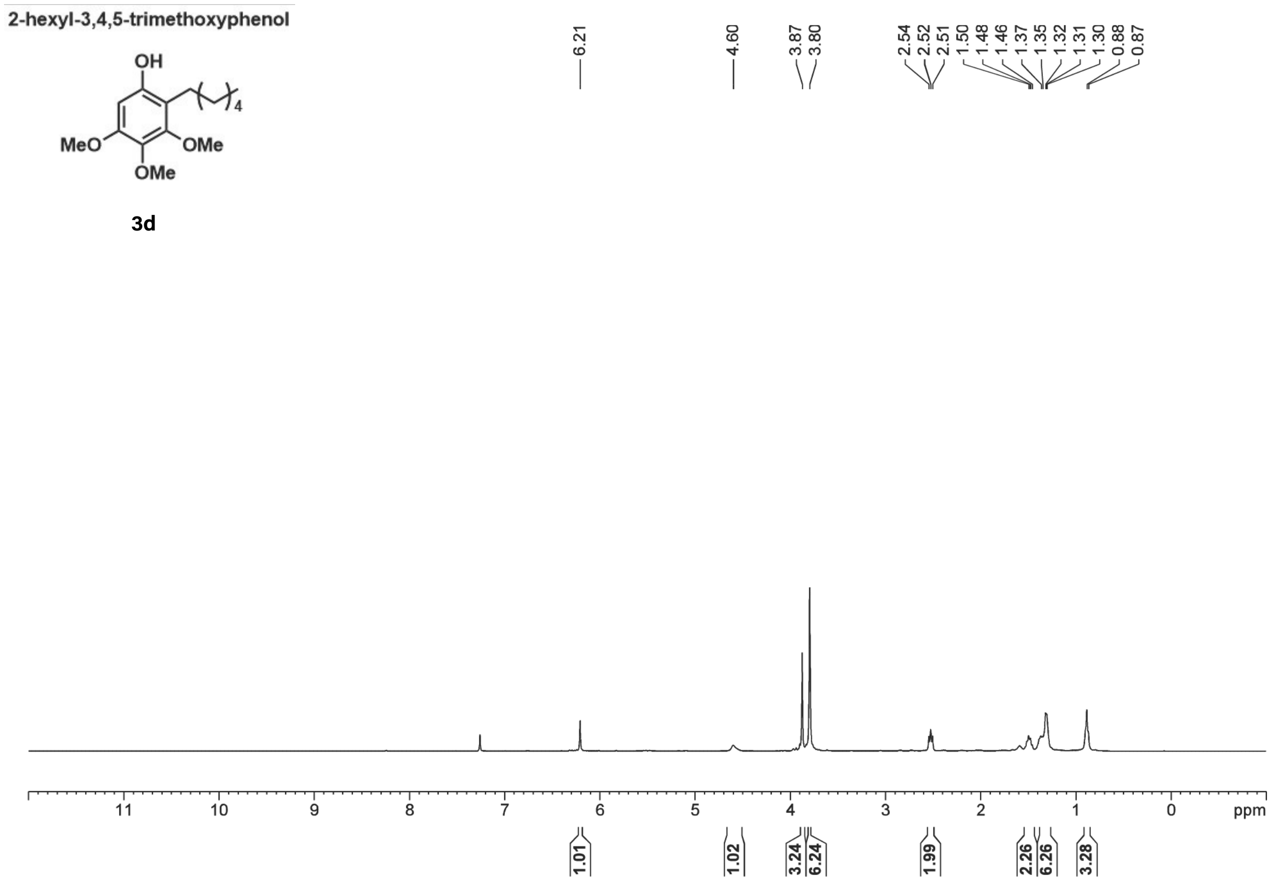

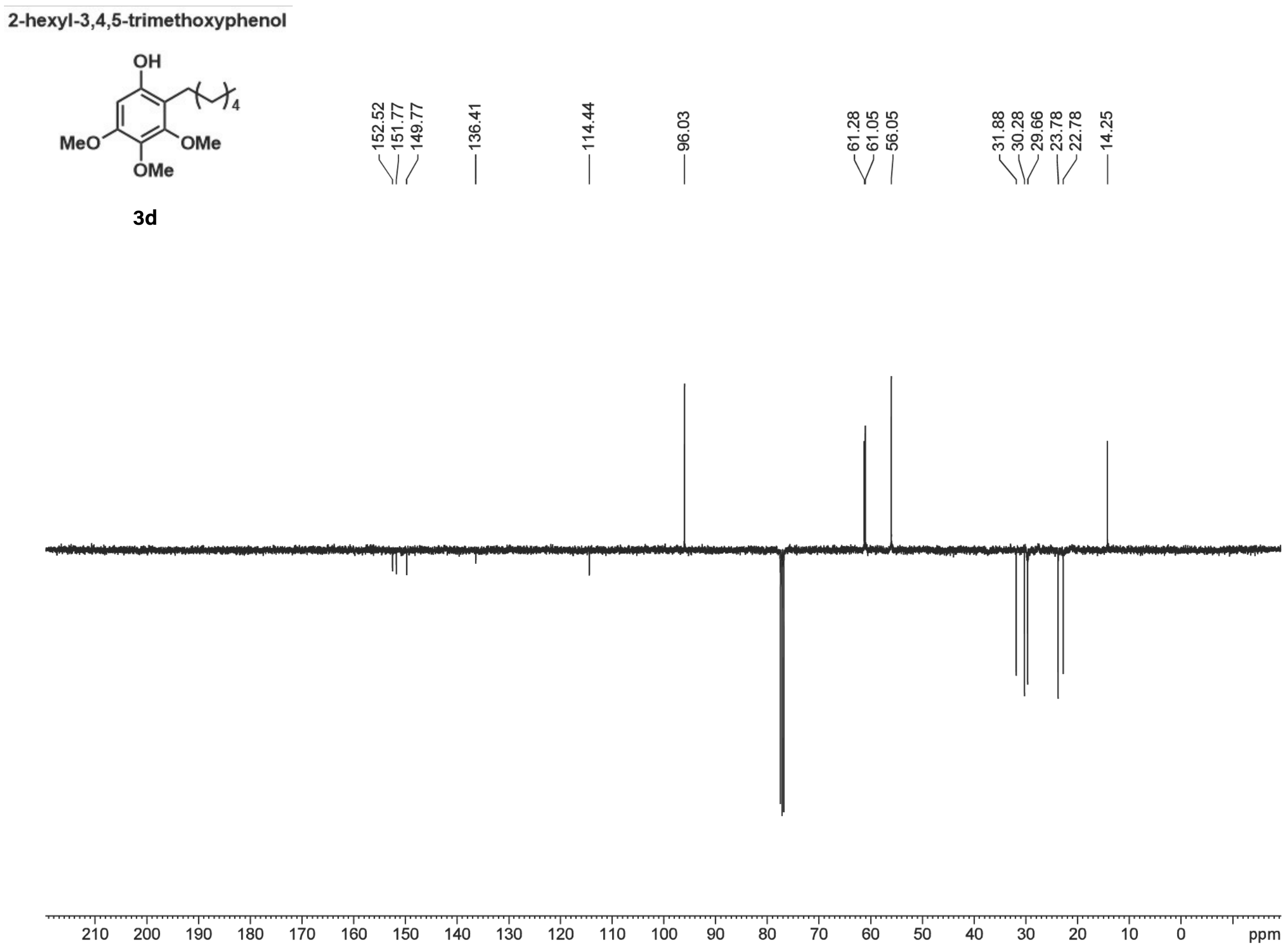

Figure S11. ^1H NMR and ^{13}C NMR of 2-hexyl-4-methylphenol (**3e**)

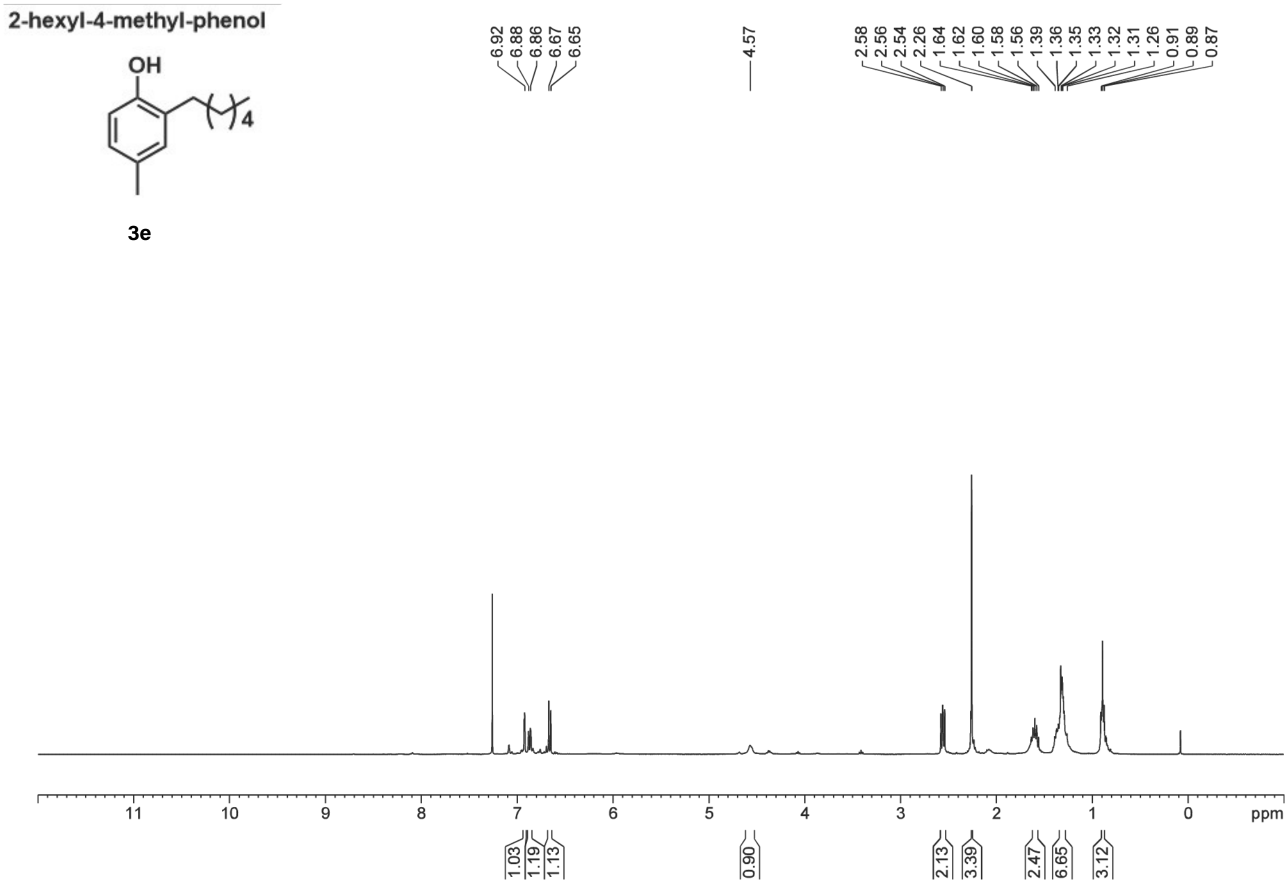

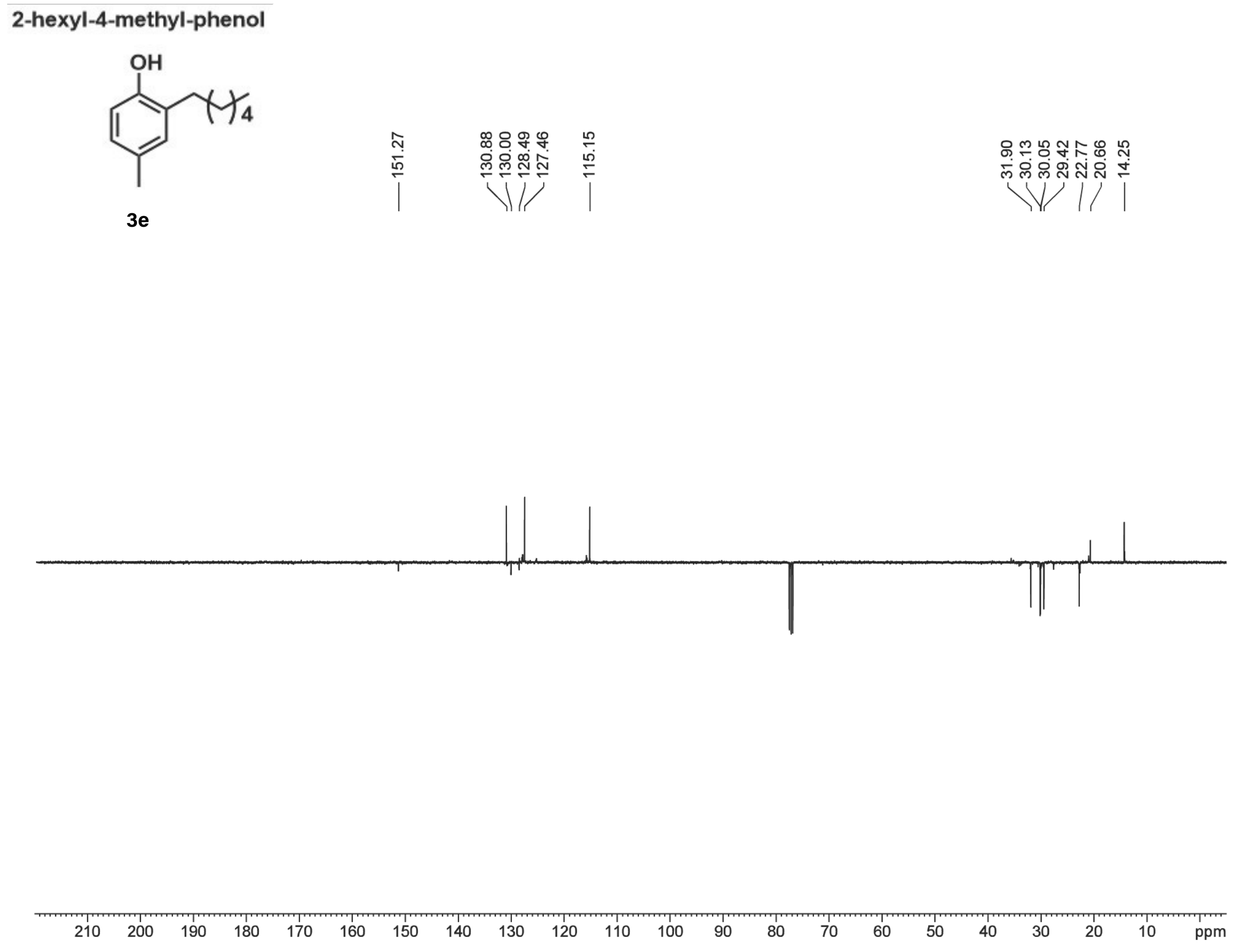

Figure S12. ^{1}H NMR and ^{13}C NMR of 2-hexyl-5-methylphenol (**3f**)

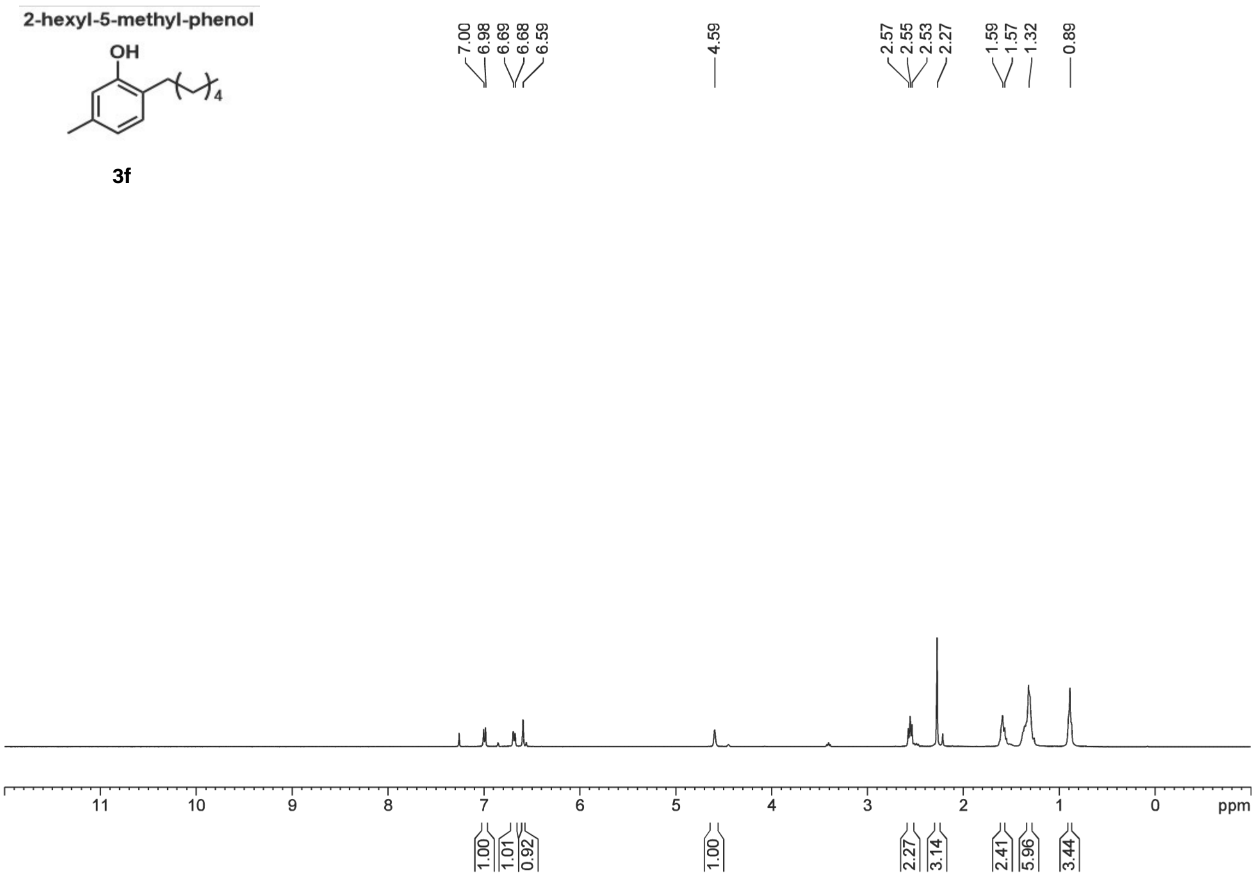

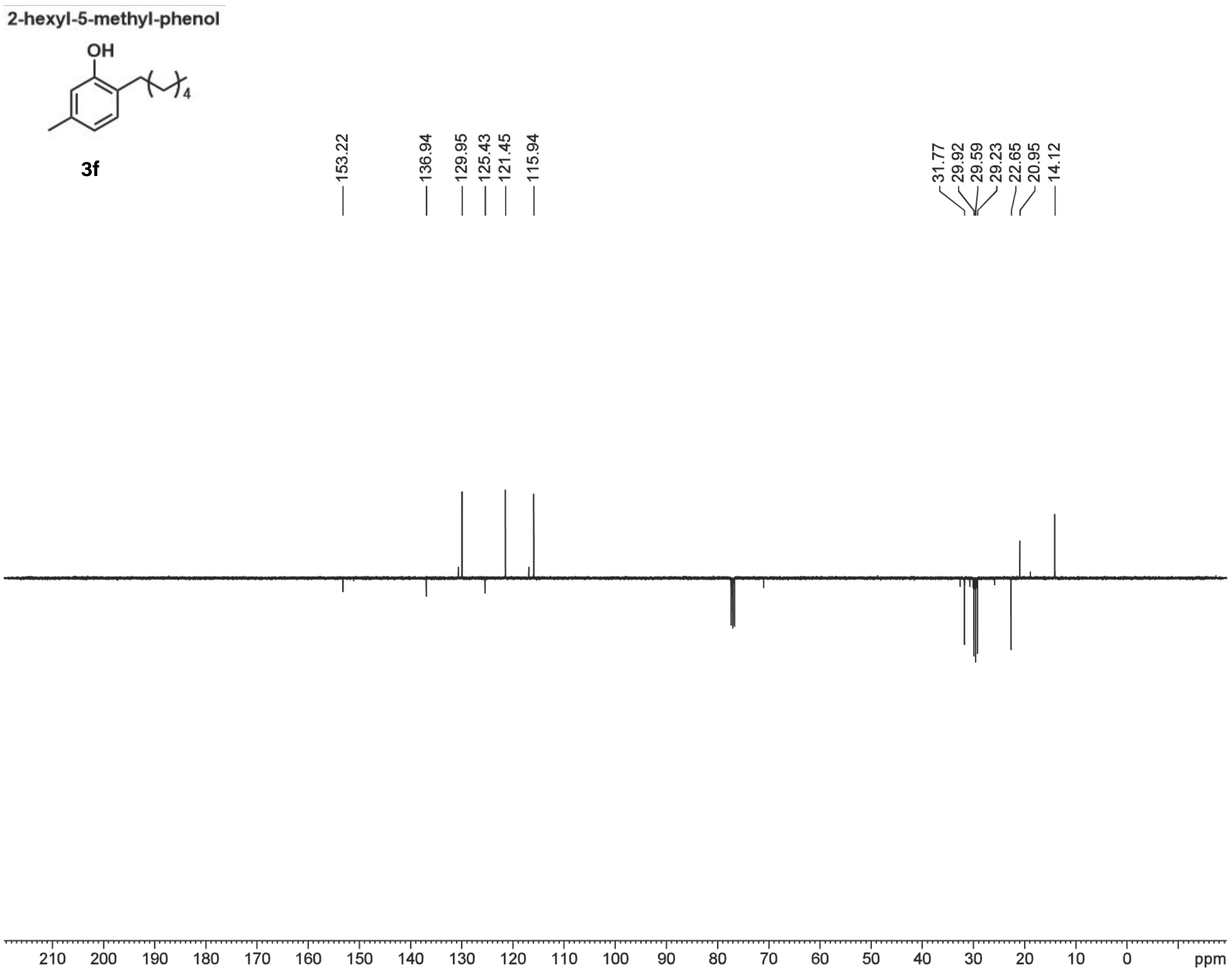

Figure S13. ^{1}H NMR and ^{13}C NMR of 2-hexyl-3,5-dimethylphenol (**3g**)

2-hexyl-3,5-dimethylphenol

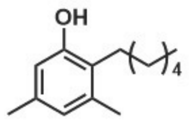

3g

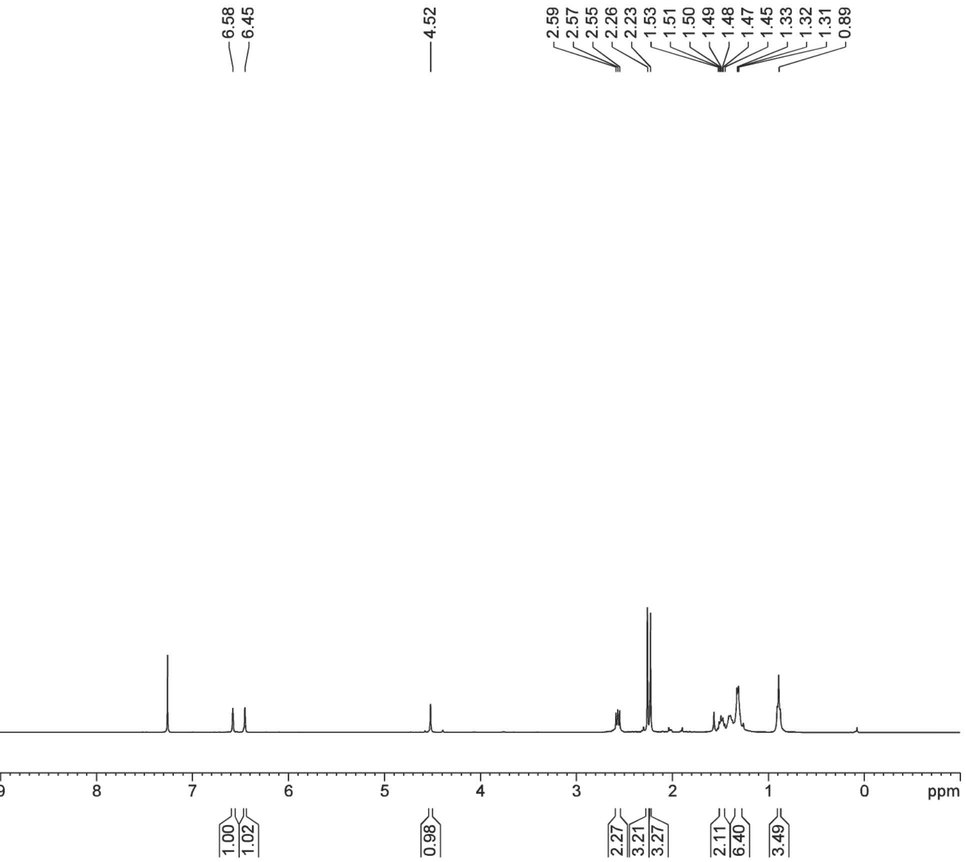

2-hexyl-3,5-dimethylphenol

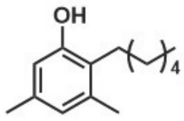

3g

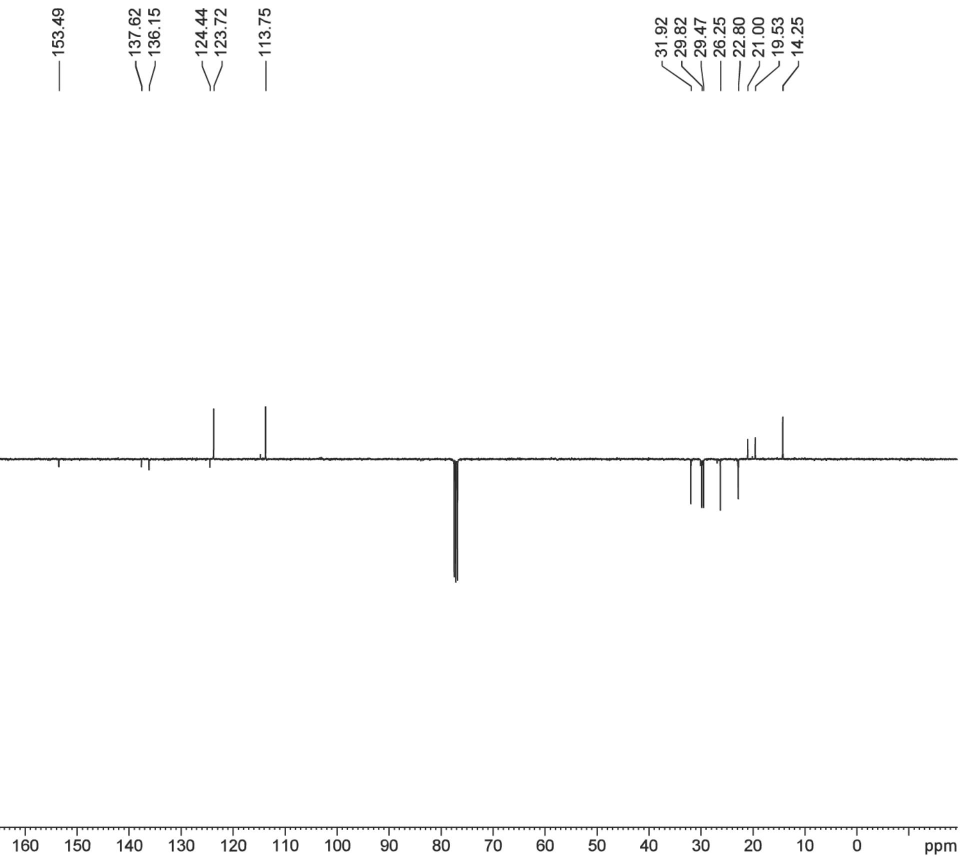

Figure S14. ^{1}H NMR and ^{13}C NMR of 2-hexyl-4-ethylphenol (**3h**)

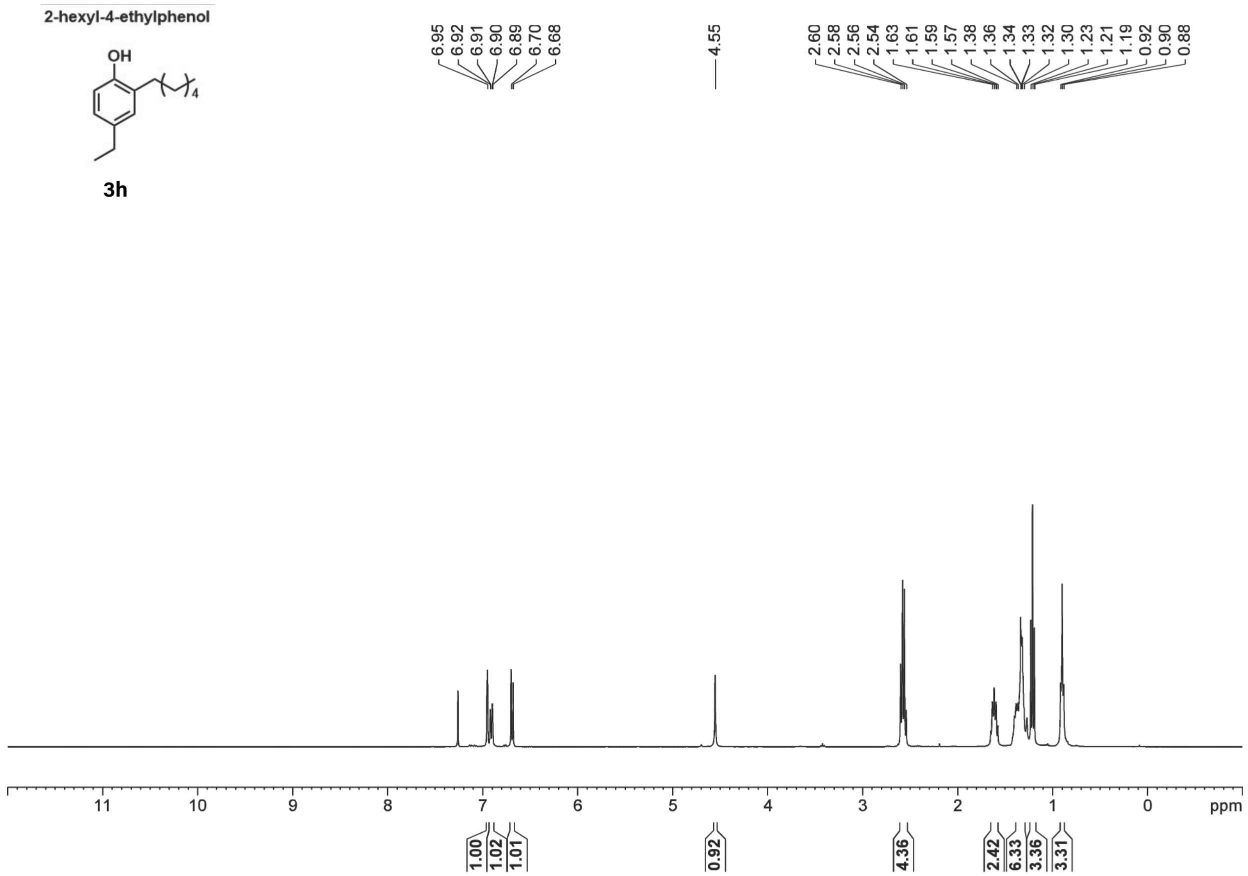

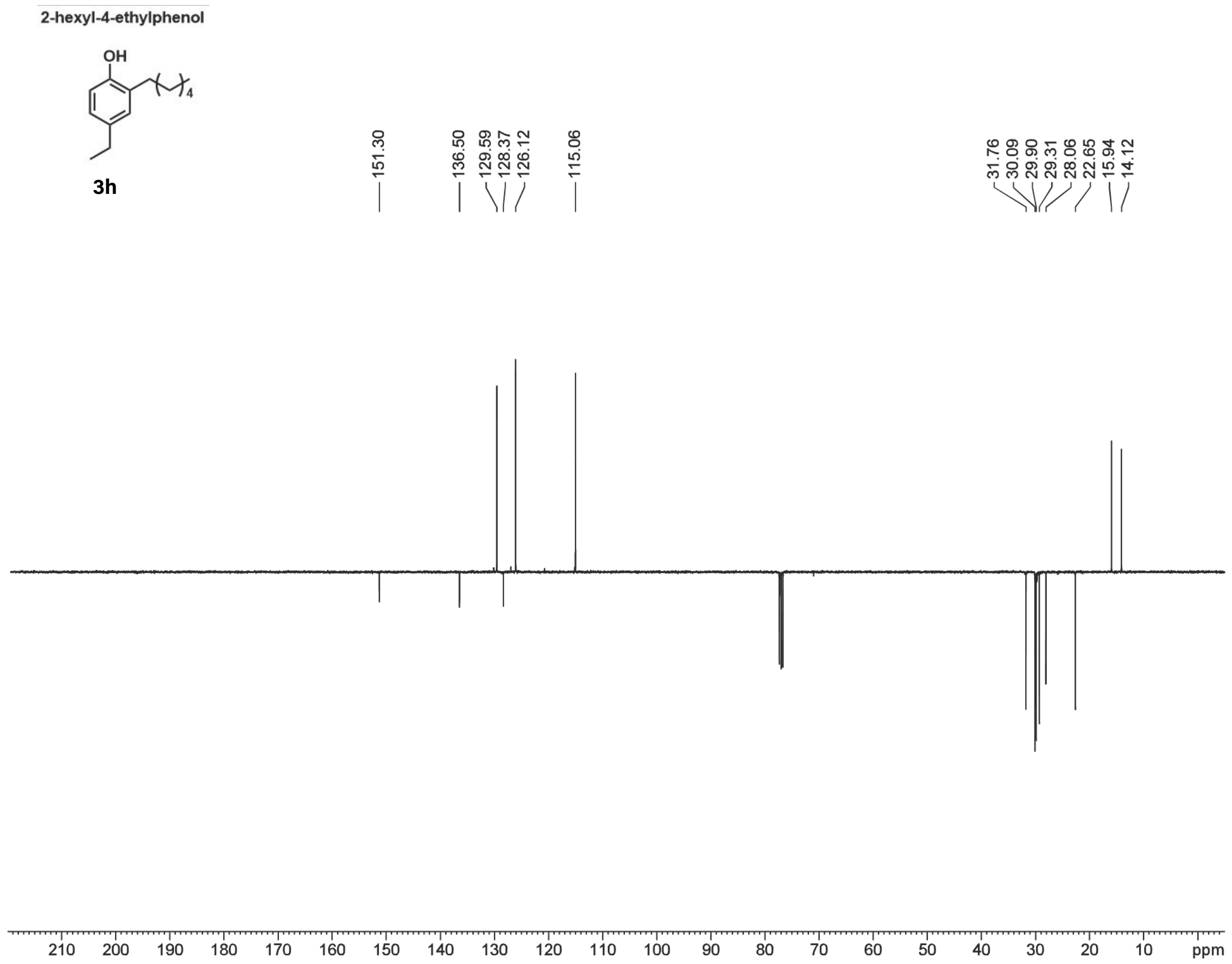

Figure S15. ^{1}H NMR and ^{13}C NMR of 2-hexyl-4-isopropylphenol (**3i**)

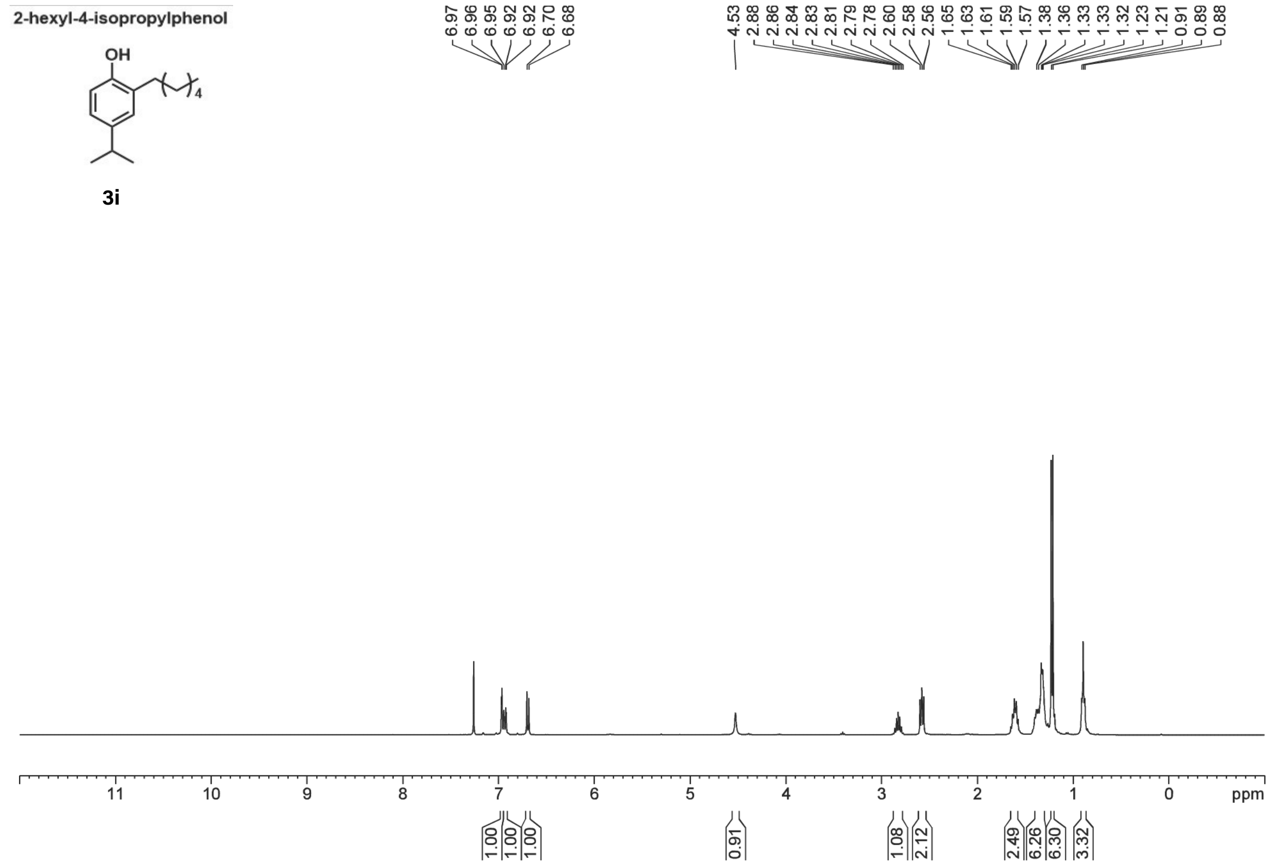

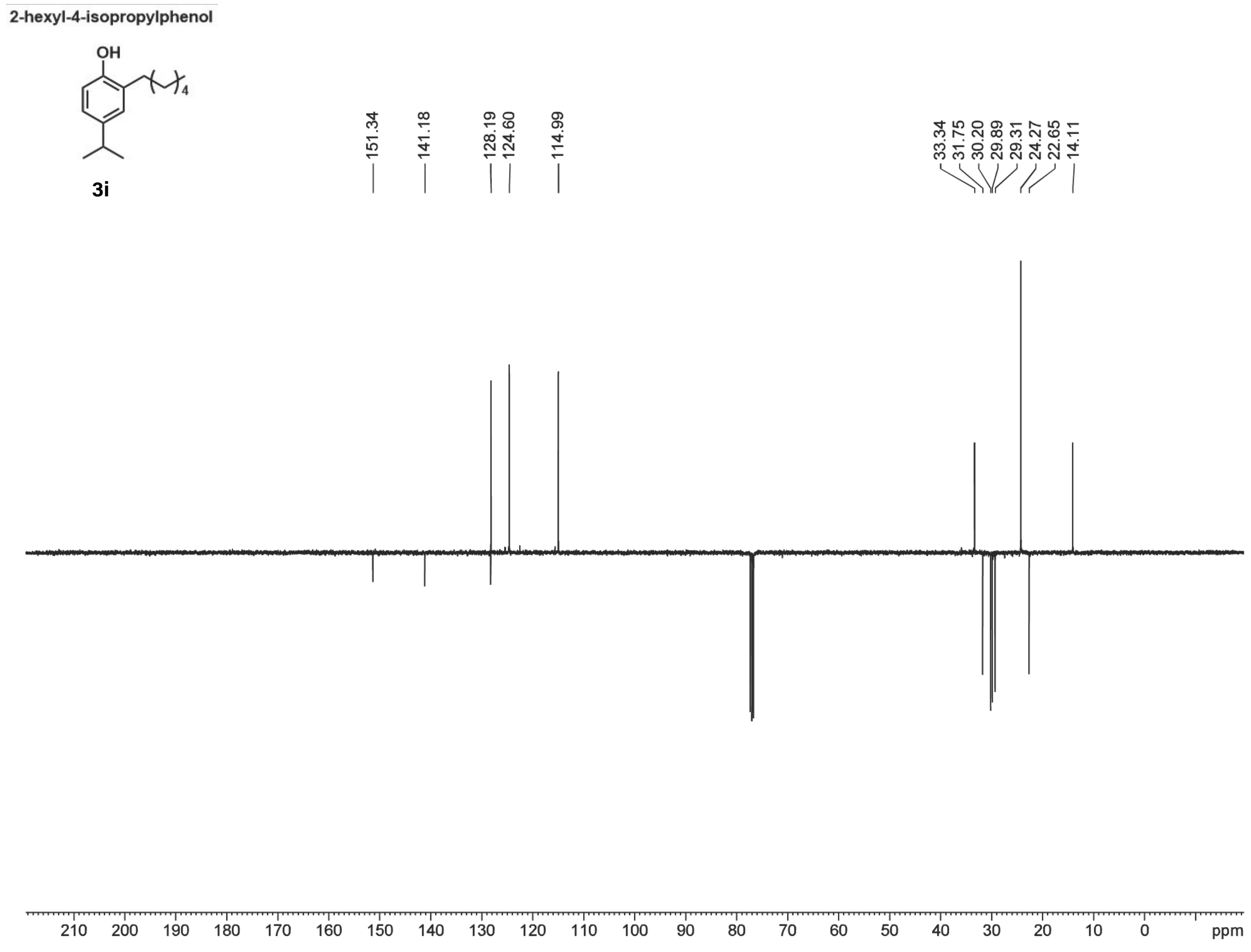

Figure S16. ^{1}H NMR and ^{13}C NMR of 2-hexyl-4-tert-butylphenol (**3j**)

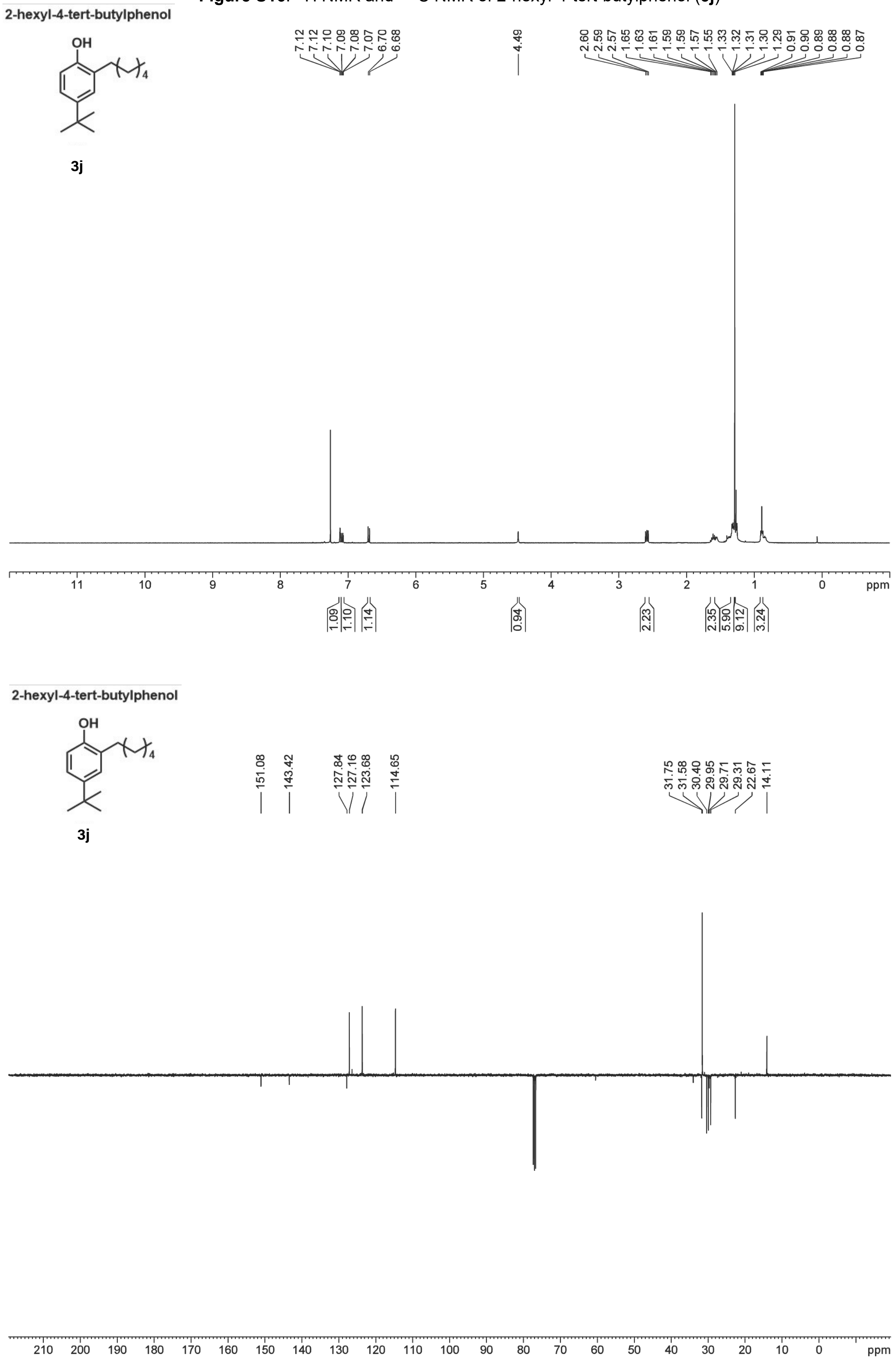

Figure S17. ^{1}H NMR and ^{13}C NMR of 2-pentyl-3,5-dimethoxyphenol (**3l**)

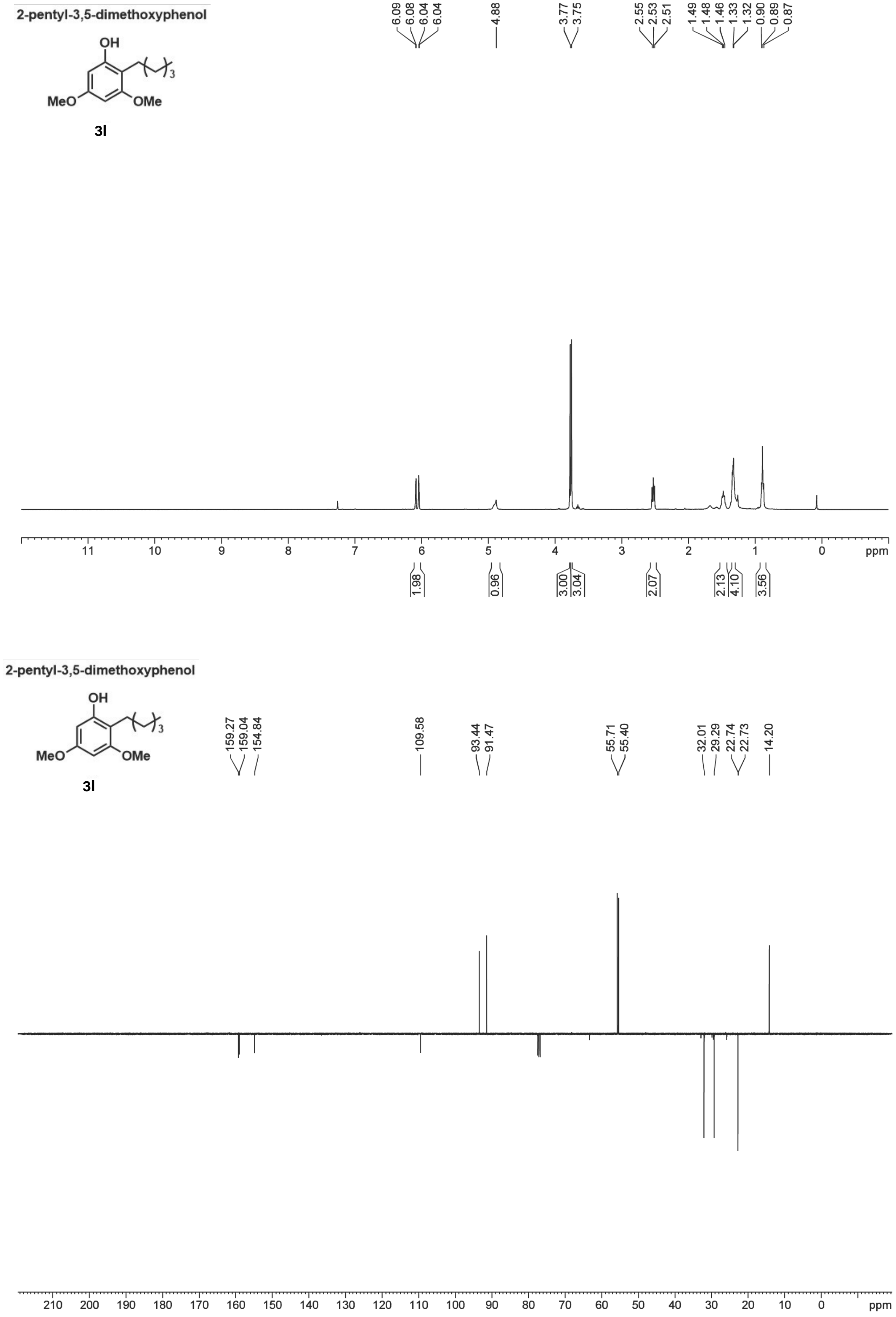

Figure S18. ^{1}H NMR and ^{13}C NMR of 2-heptyl-3,5-dimethoxyphenol (**3m**)

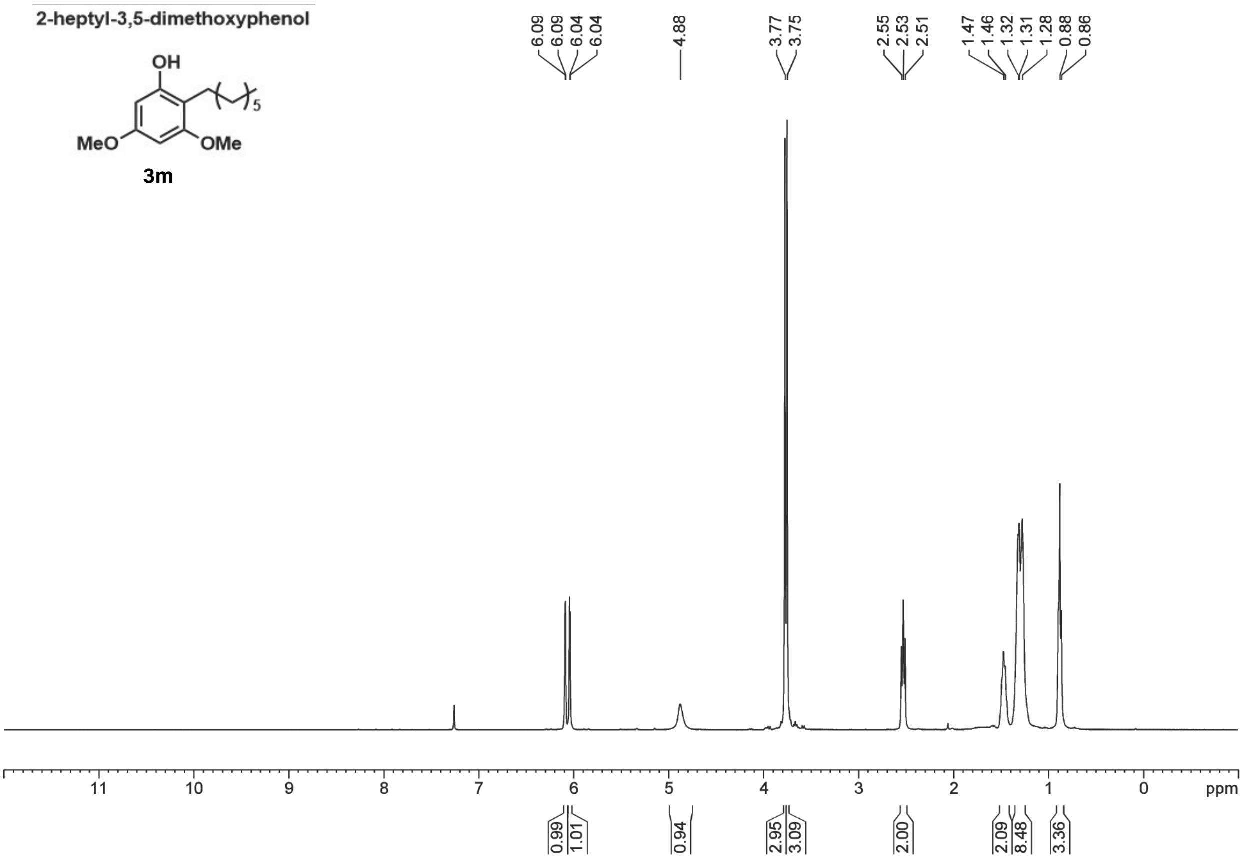

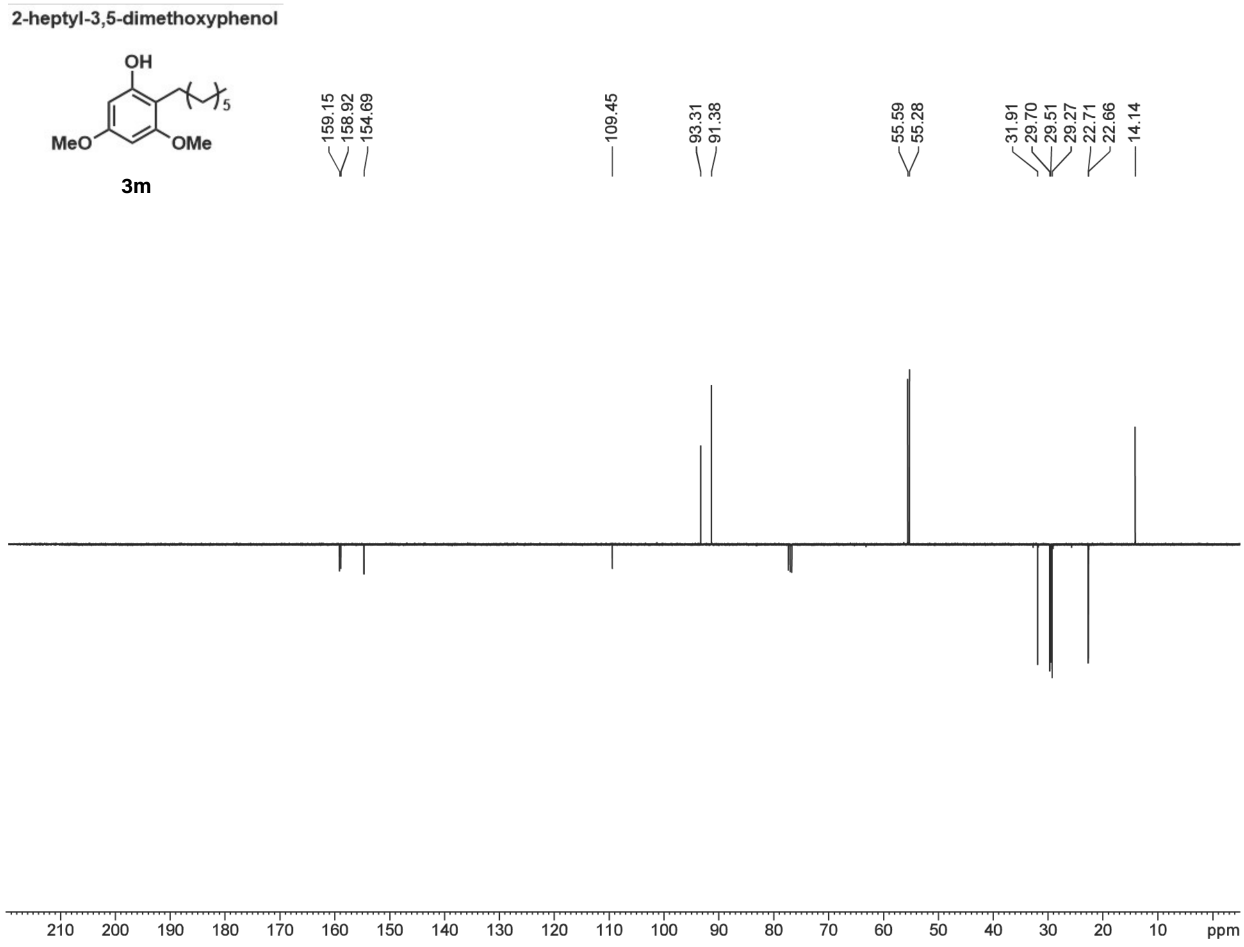

Figure S19. ^{1}H NMR and ^{13}C NMR of 2-octyl-3,5-dimethoxyphenol (**3n**)

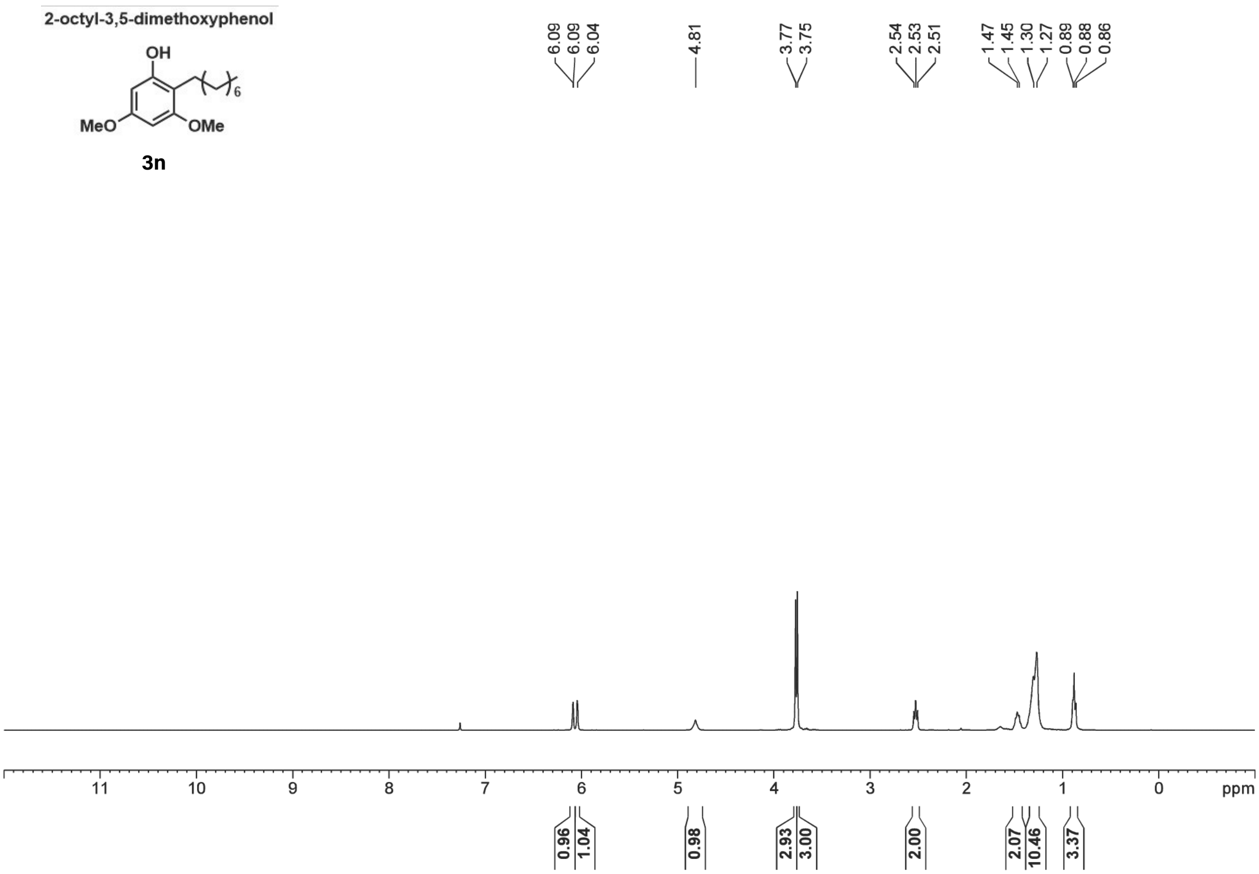

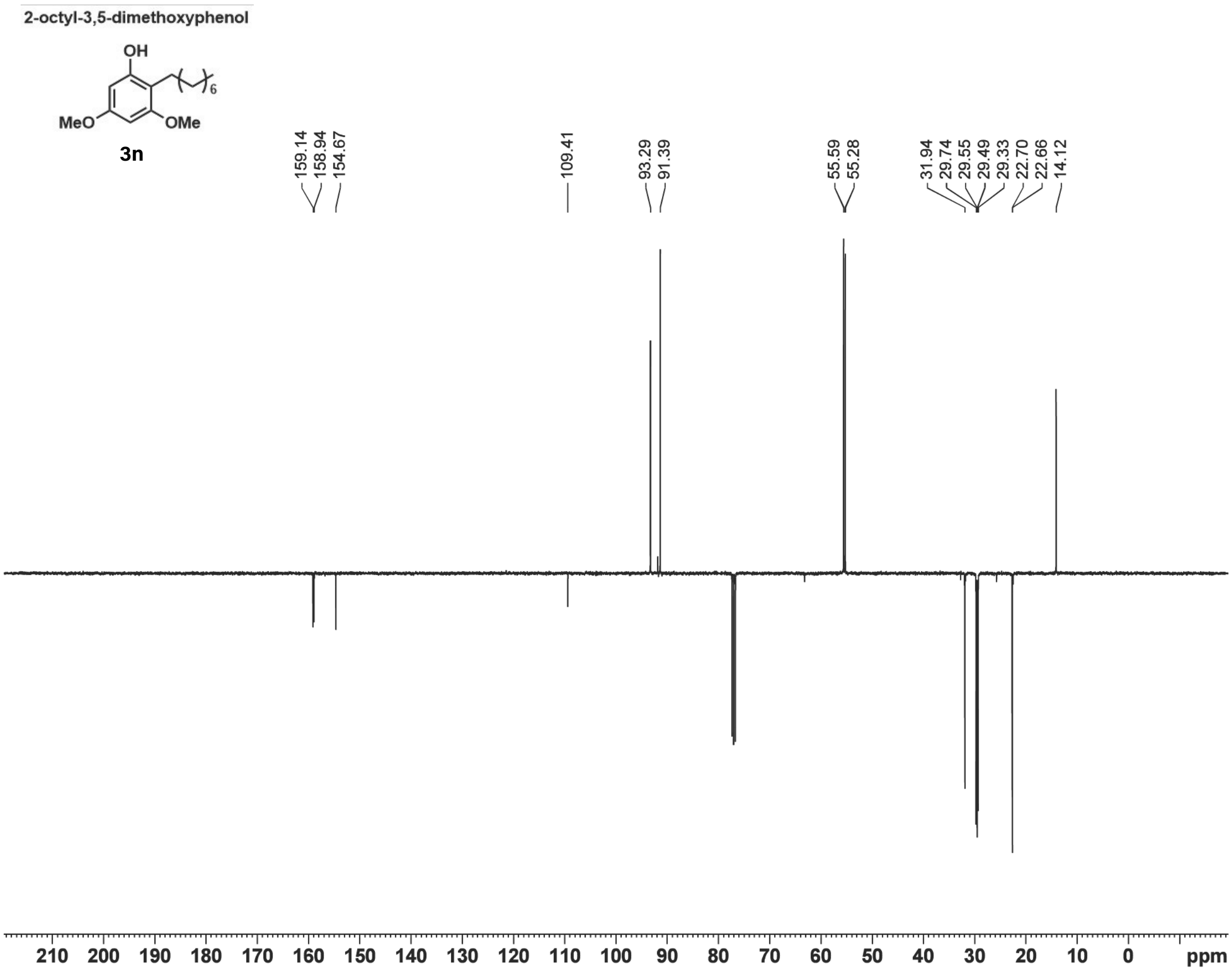

Figure S20. ^{1}H NMR and ^{13}C NMR of 3,5-dimethoxy-2-(3,5,5-trimethylhexyl)phenol (**3o**)

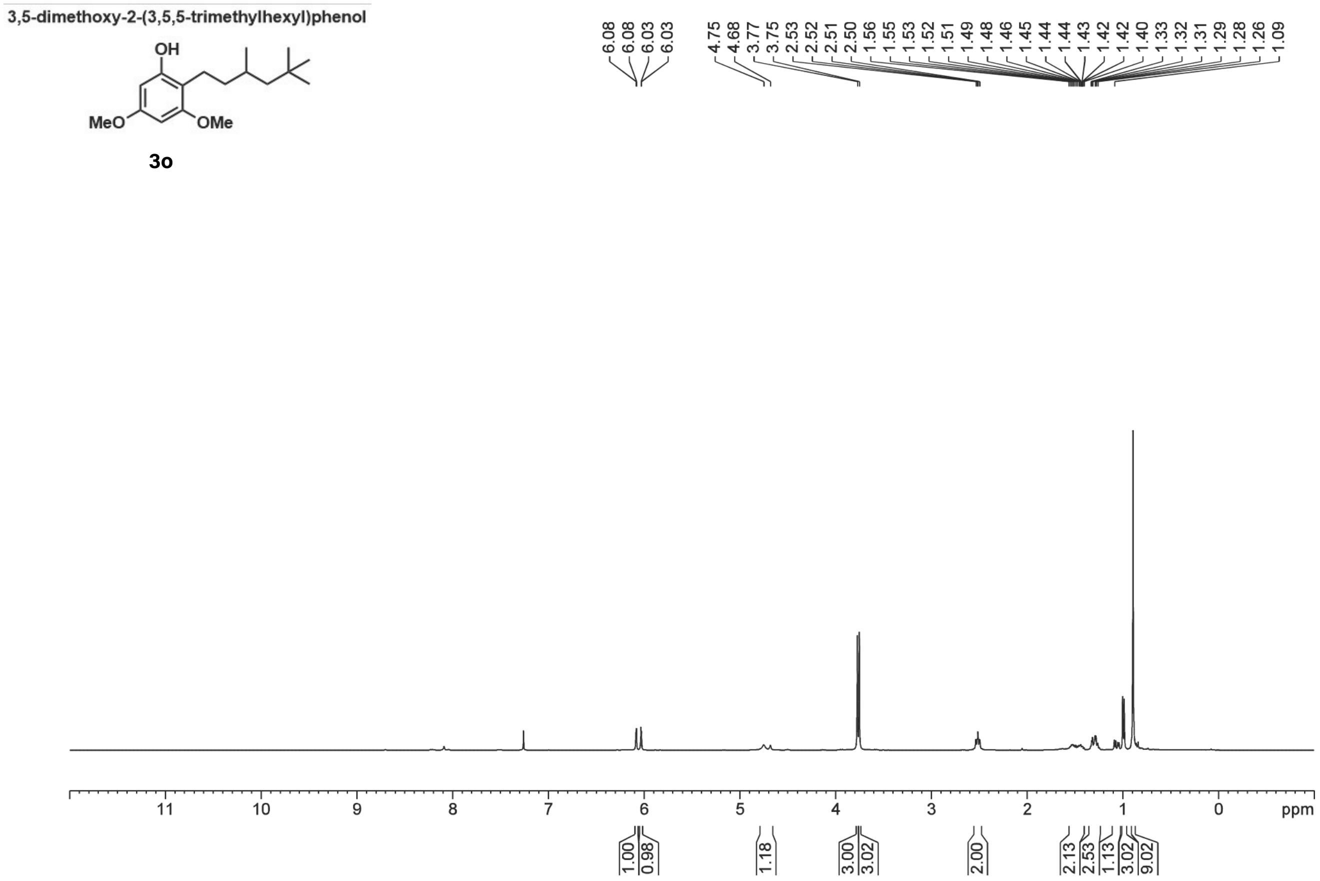

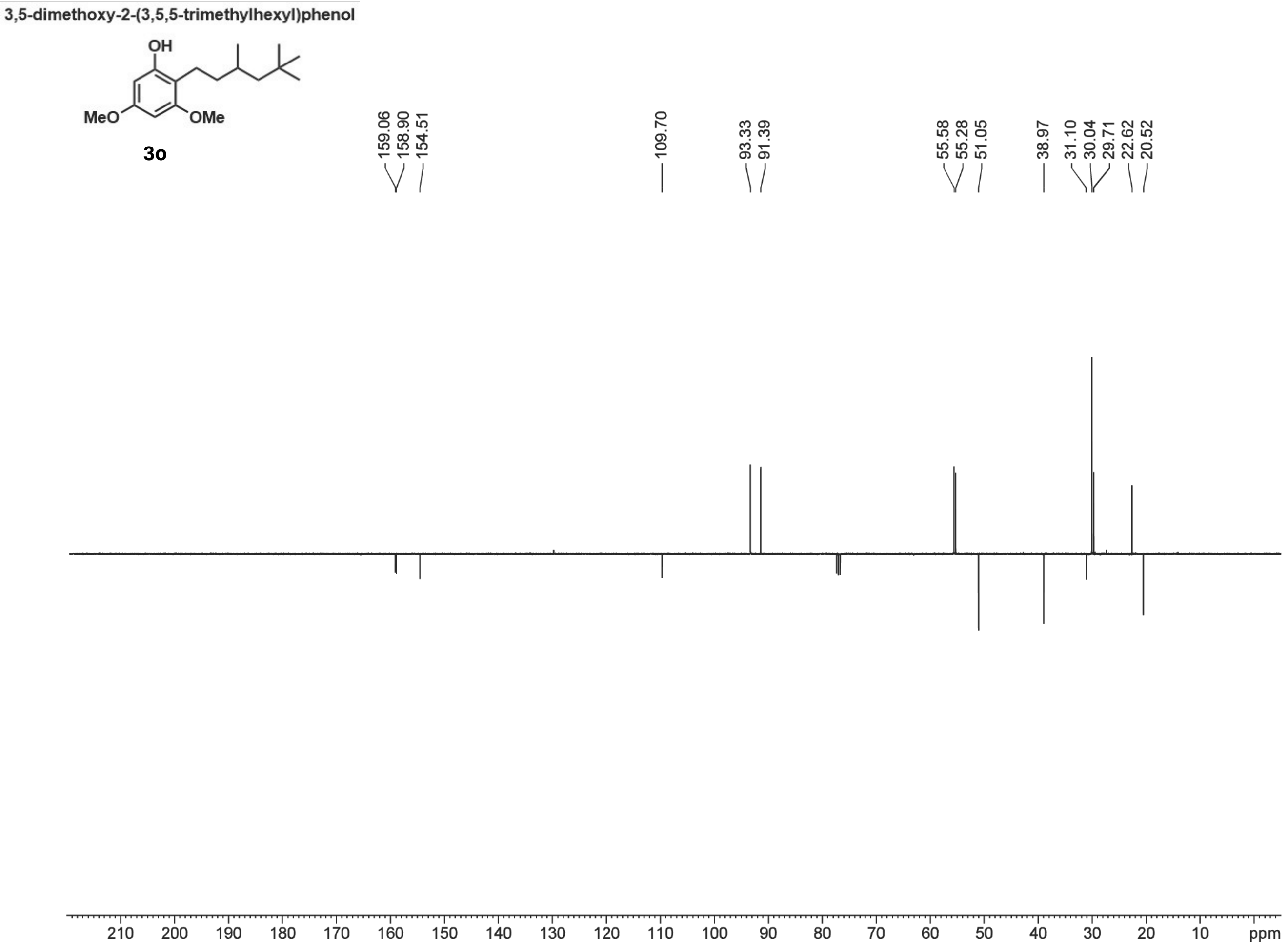

Figure S21. ^{1}H NMR and ^{13}C NMR of 2-isopentyl-3,5-dimethoxyphenol (**3p**)

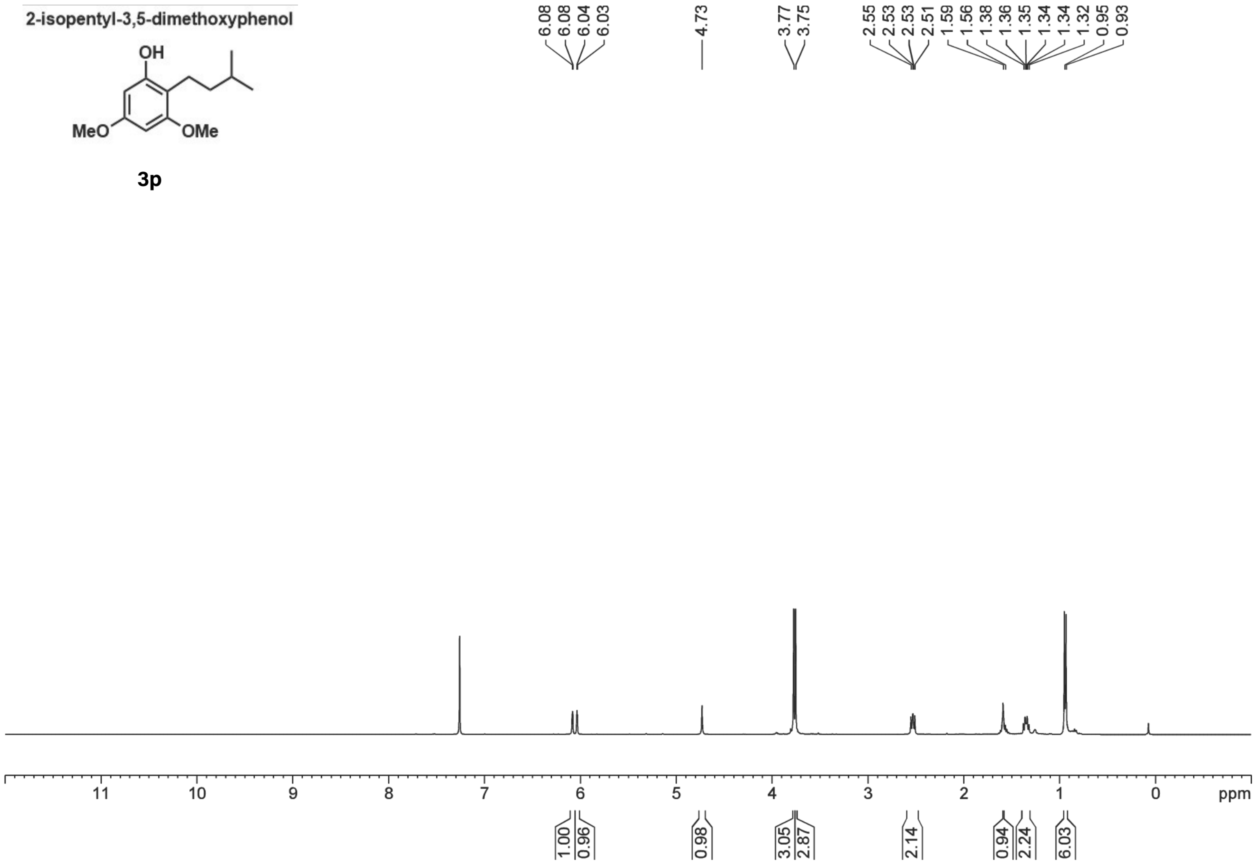

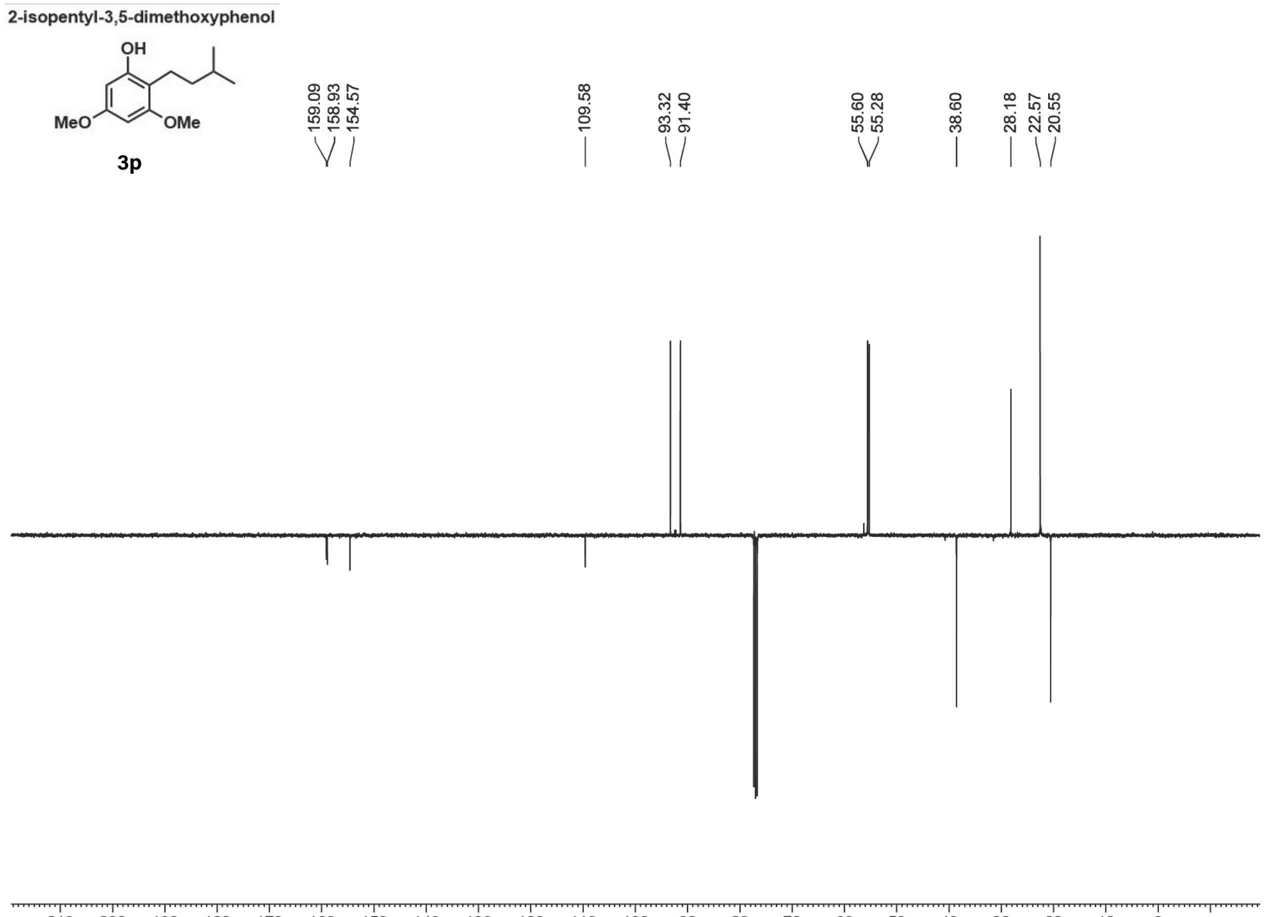

Figure S22. ^{1}H NMR and ^{13}C NMR of 2-pentyl-3,4,5-dimethoxyphenol (**3q**)

2-pentyl-3,4,5-trimethoxyphenol

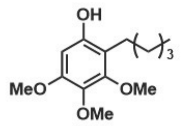

3q

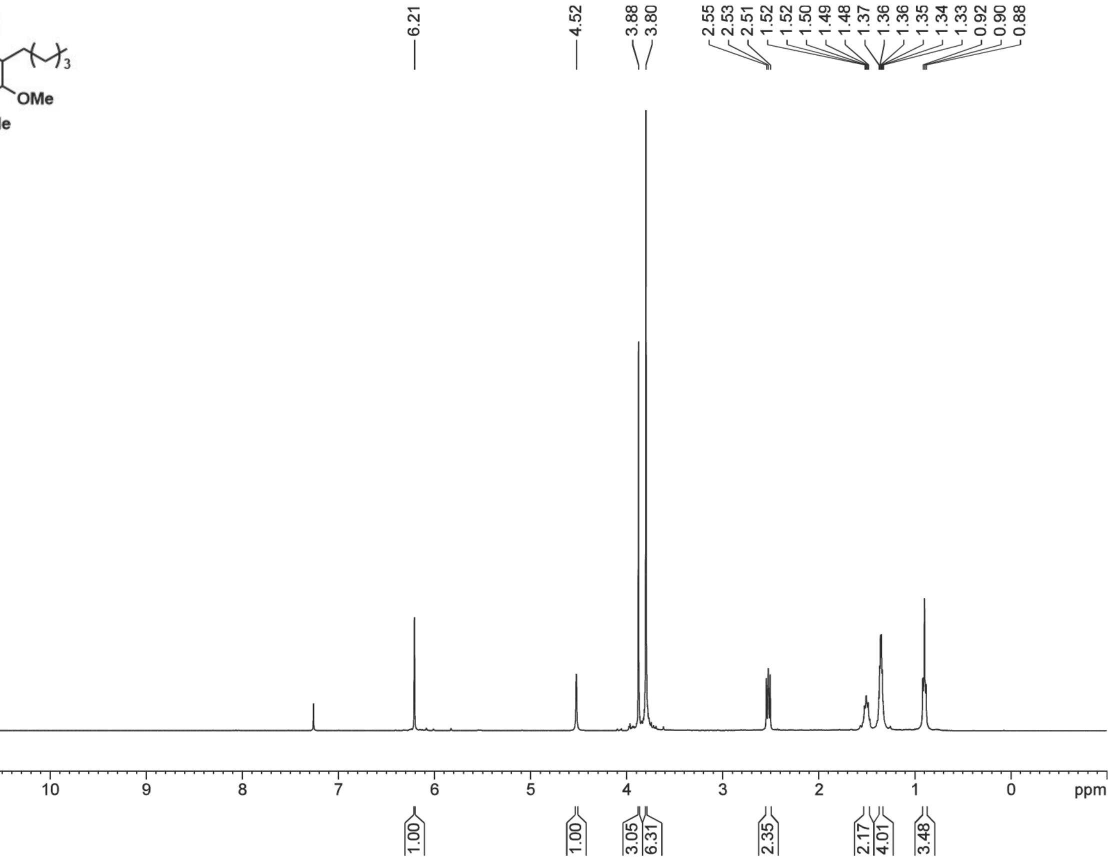

2-pentyl-3,4,5-trimethoxyphenol

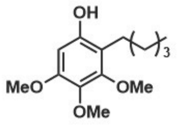

3q

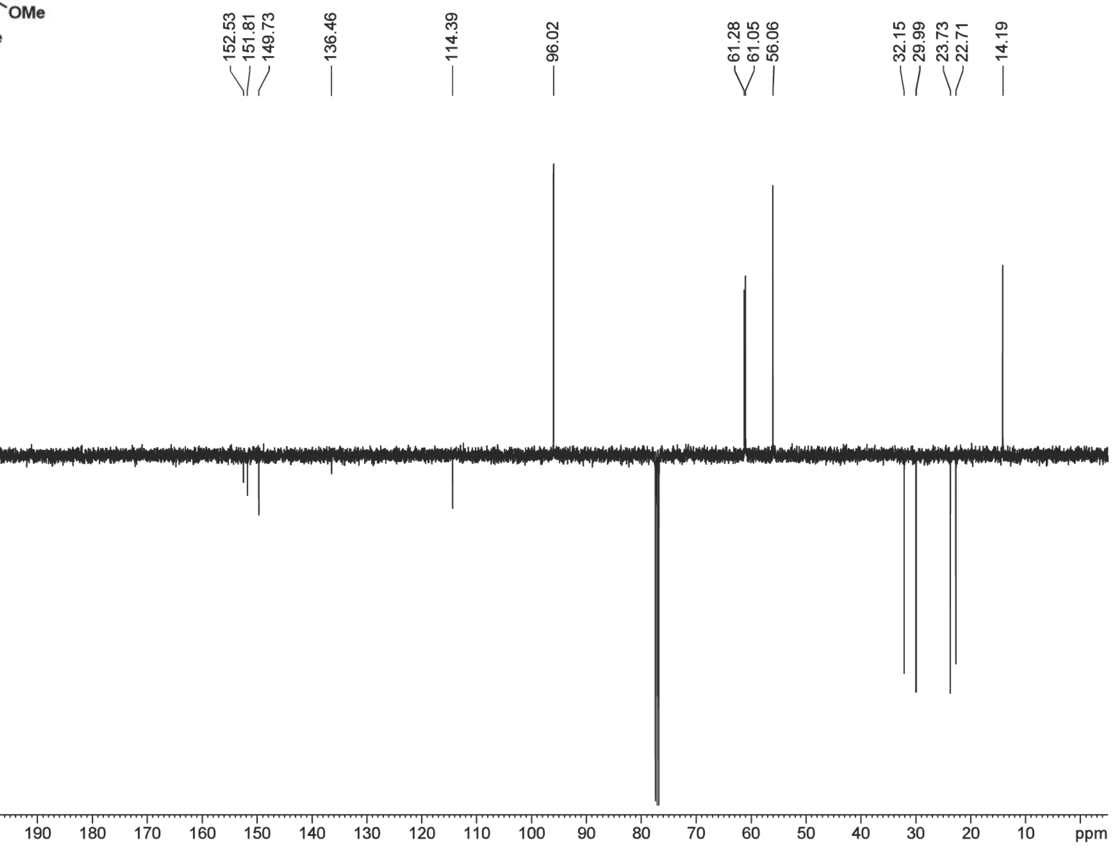

Figure S23. ^{1}H NMR and ^{13}C NMR of 2-heptyl-3,4,5-dimethoxyphenol (**3r**)

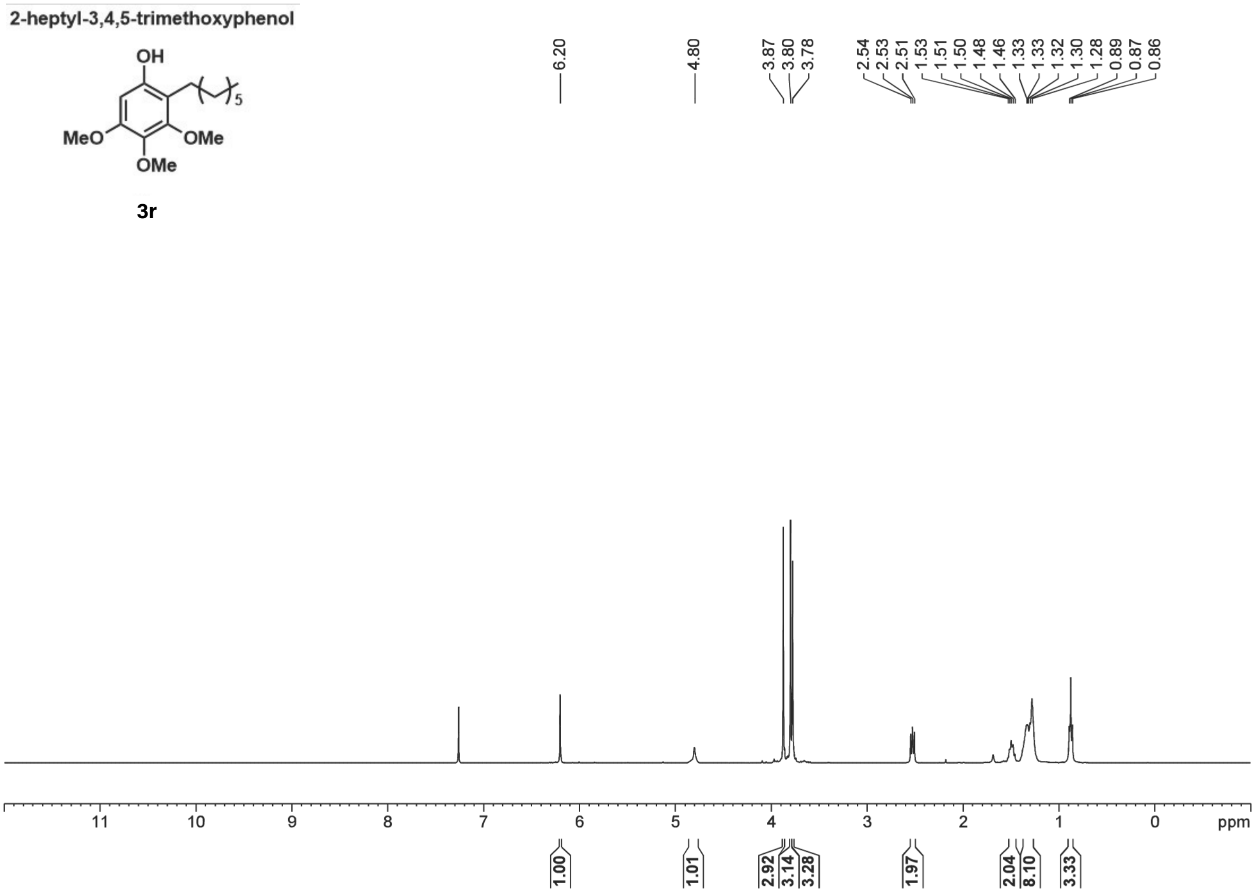

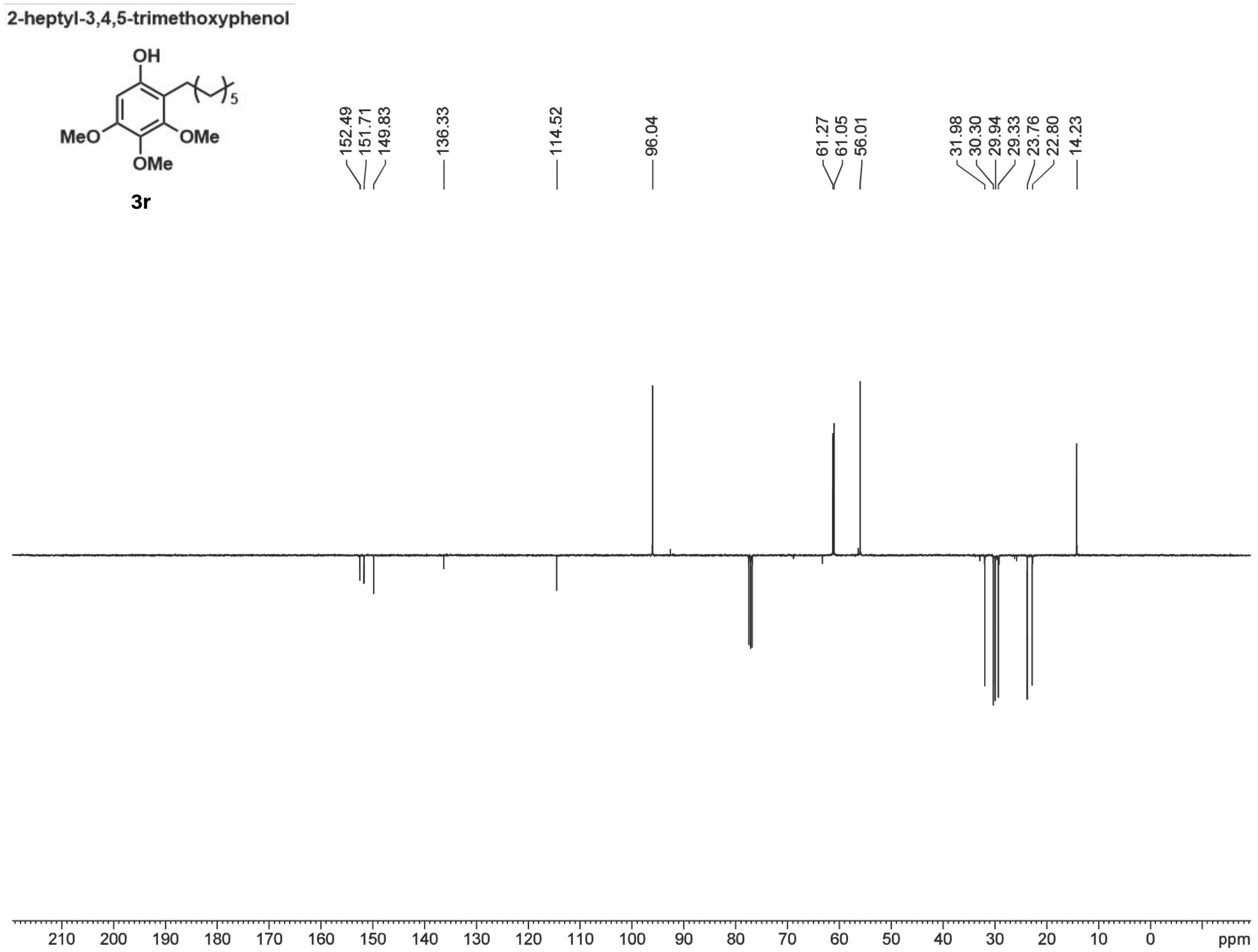

Figure S24. ^{1}H NMR and ^{13}C NMR of 2-octyl-3,4,5-dimethoxyphenol (**3s**)

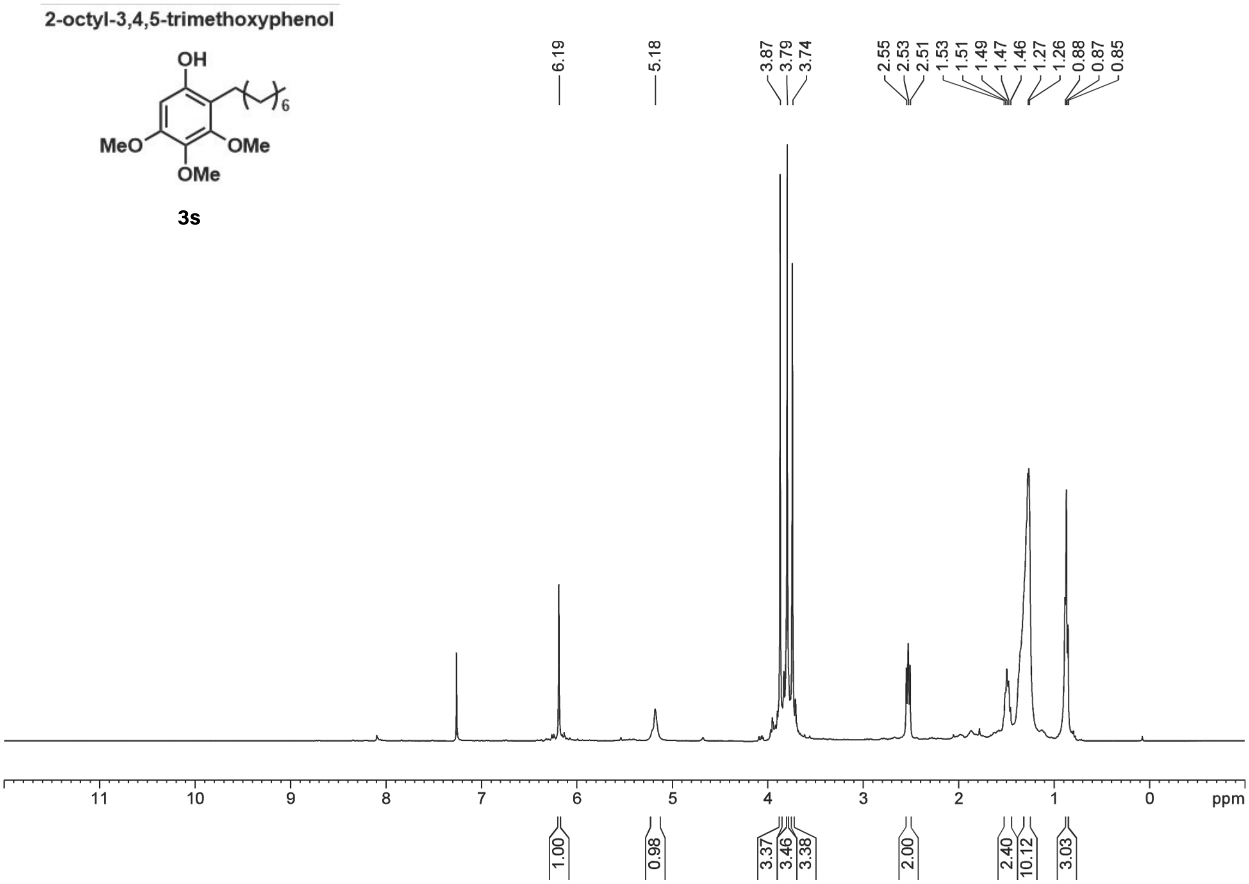

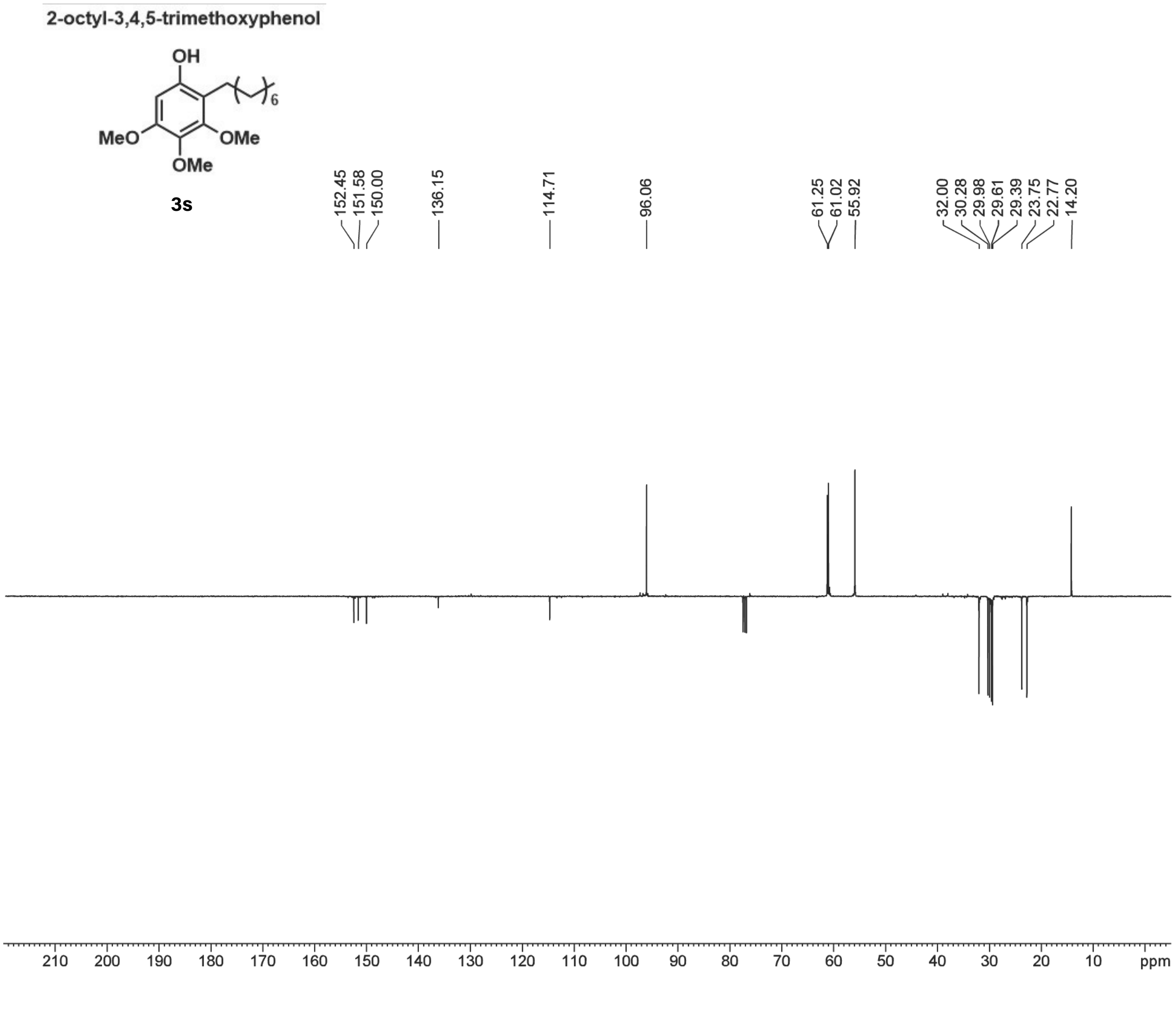

Figure S25. ^{1}H NMR and ^{13}C NMR of 2-isopentyl-3,4,5-dimethoxyphenol (**3t**)

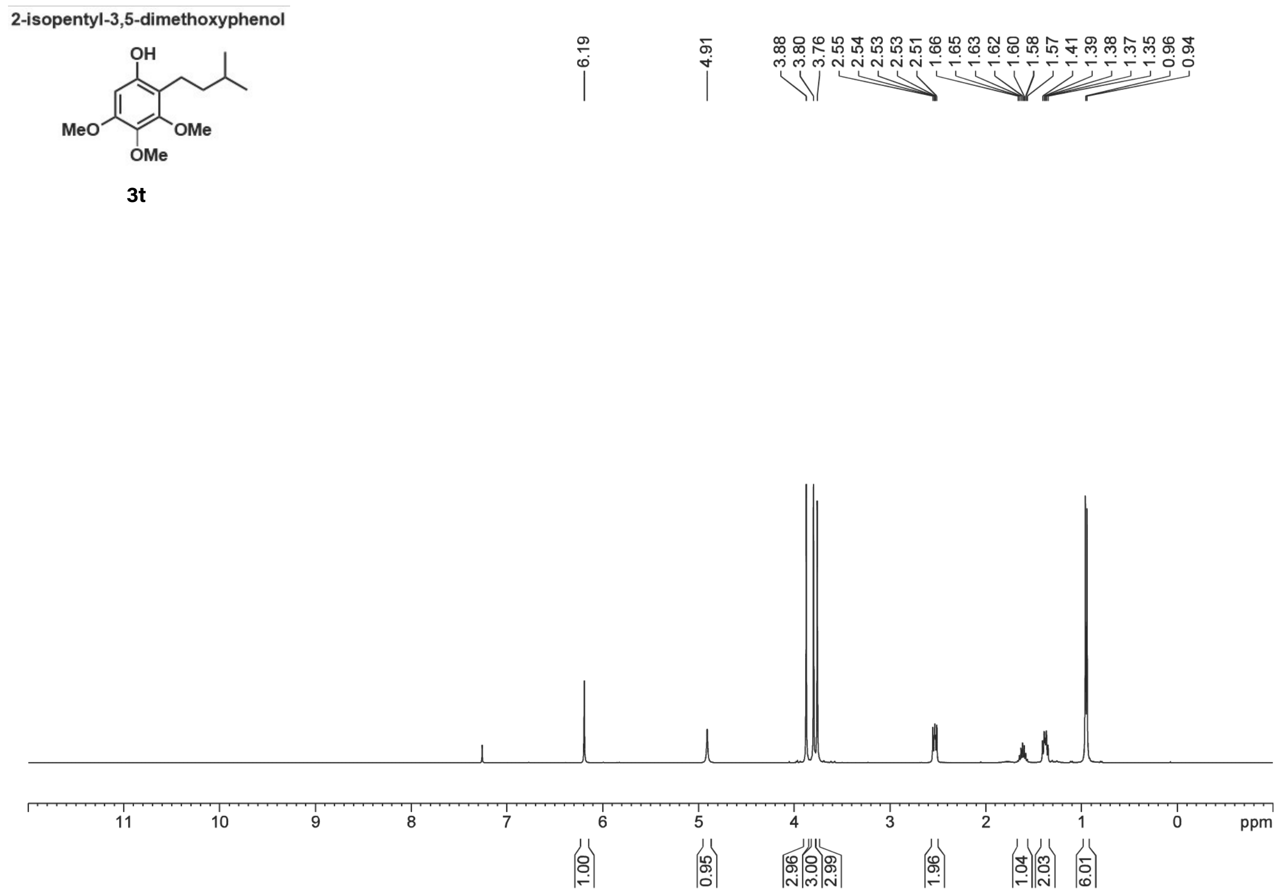

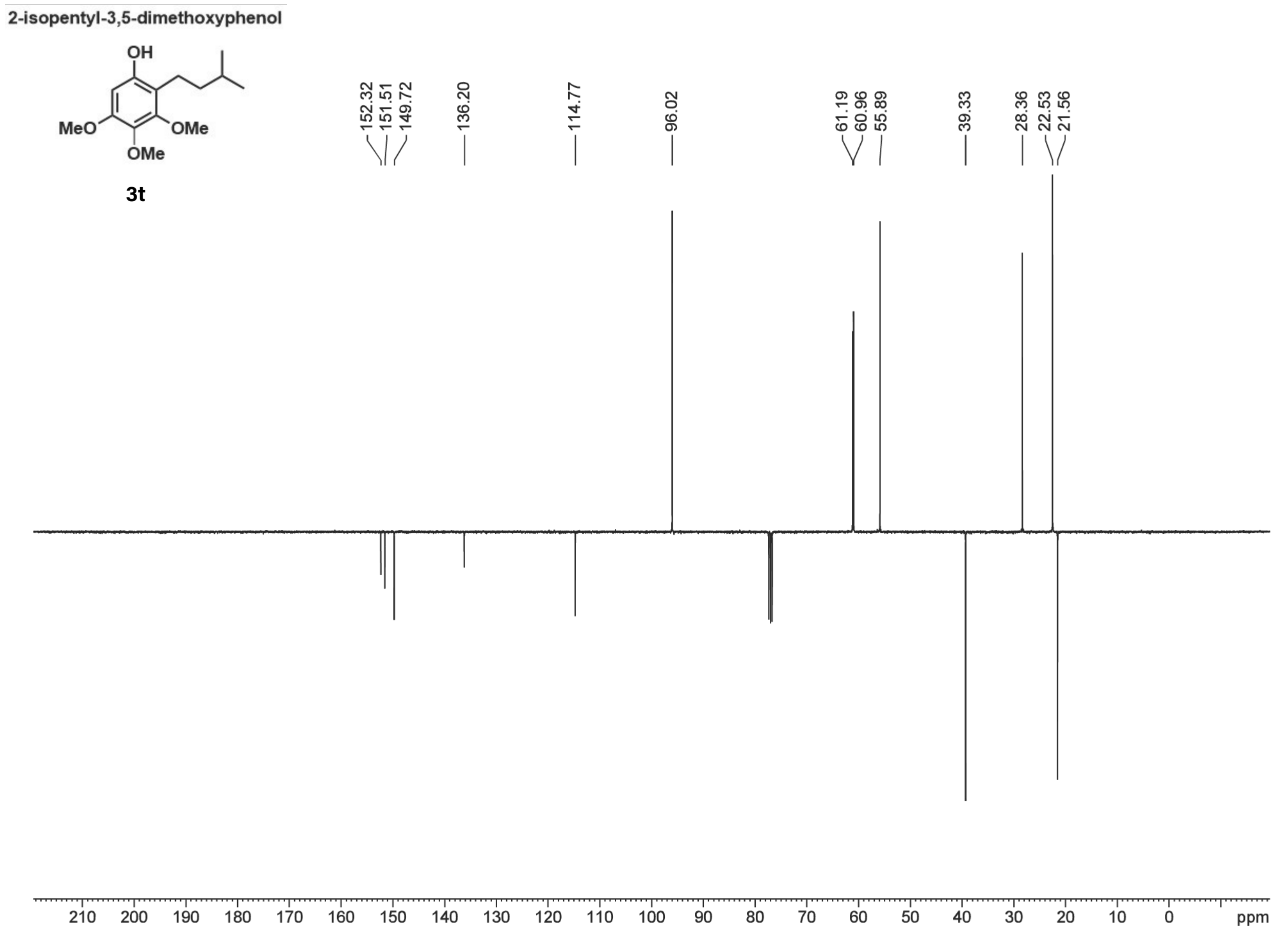

Figure S26. ^{1}H NMR and ^{13}C NMR of amyl-*meta*-cresol (**3u**)

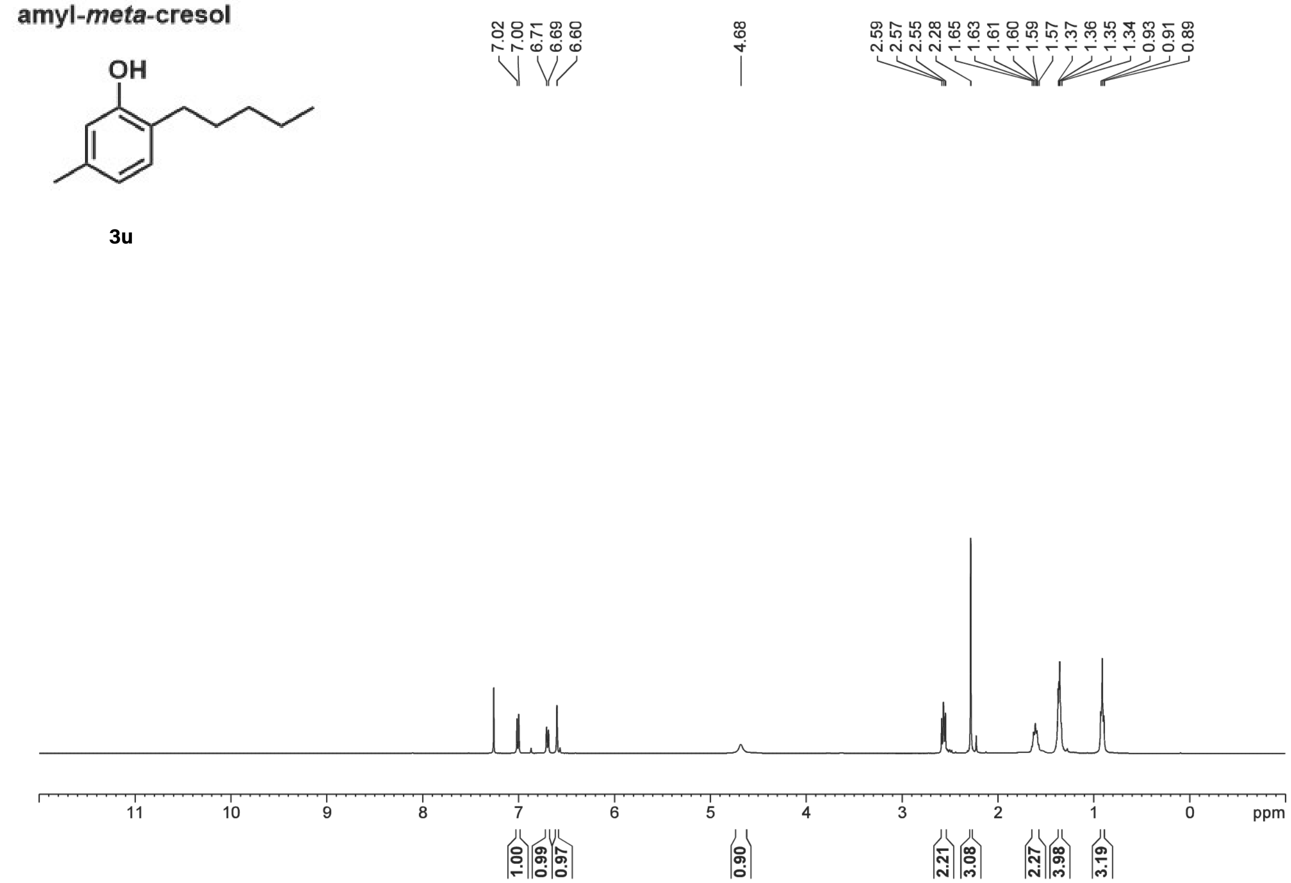

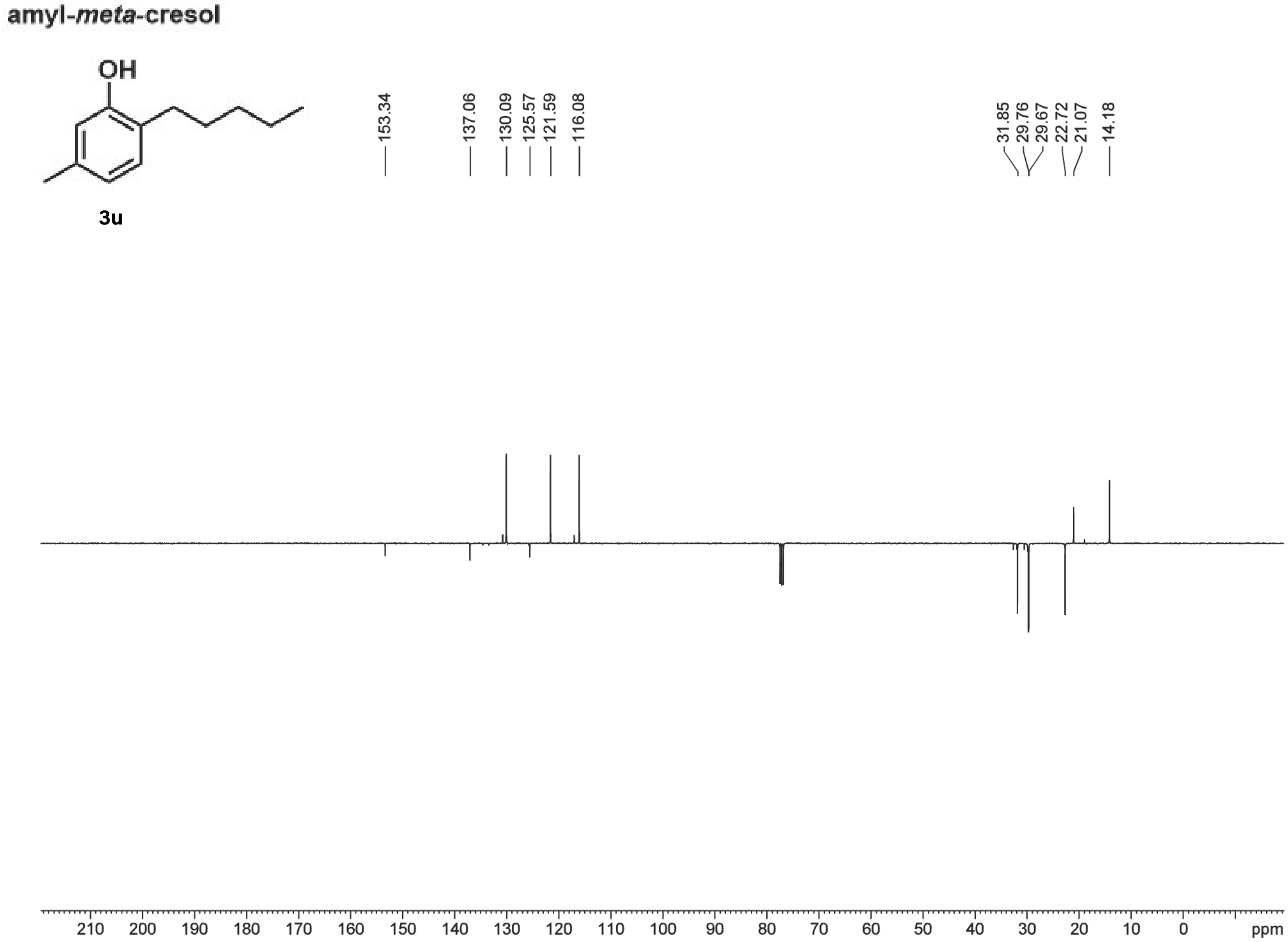

15. Chem21 Qualitative Evaluation

Table S27. *J. Am. Chem. Soc.* **2018**, *140*, 32, 10289–10296

Solvents (First Pass)

		List solvents below
**Preferred solvents**	**water,** EtOH, nBuOH, AcOipr, AcOnBu, PhOMe, MeOH, tBuOH, BnOH, ethylene glycol, acetone, MEK, MIBK, **AcOEt,** sulfolane	
**Problematic solvents:** (acceptable only if substitution does not offer advantages)	DMSO, cyclohexanone, DMPU, AcOH, Ac2O, Acetonitrile, AcOMe, THF, heptane, Me-cyclohexane, toluene, xylene, MTBE, **cyclohexane**, chlorobenzene, formic acid, pyridine, Me-THF	
**Hazardous solvents**: These solvents have significant health and/or safety concerns.	dioxane, pentane, TEA, diisopropyl ether, DME, DCM, DMF, DMA, NMP, methoxyethanol, hexane	DCM
**Highly hazardous solvents:** The solvents which are agreed not to be used, even in screening	Et_2O, Benzene, CCl_4, chloroform, DCE, nitromethane, CS_2, HMPA	DCE

Experimental:
'In a glove box, a phenol (0.5 mmol), a ketone (1.0 mmol), and [(C6H6)(PCy3)(CO)RuH]+ BF4 − (9 mg, 3 mol %) were dissolved in 1,2- dichloroethane (2 mL) in a 25 mL Schlenk tube equipped with a Teflon stopcock and a magnetic stirring bar. The tube was brought out of the glove box, and was stirred in an oil bath preset at 125-140 °C for 16-72 h. The reaction tube was taken out of the oil bath and was cooled to room temperature. After the tube was open to air, the solution was filtered through a short silica gel column by eluting with CH2Cl2 (10 mL), and the filtrate was analyzed by GC-MS. Analytically pure product was isolated by column chromatography on silica gel (230-460 mesh, hexanes/EtOAc). '

Catalyst/enzyme (First Pass)

		Tick
Catalyst or enzyme used, **or** reaction takes place	Green Flag	x
Use of stoichiometric quantities of reagents	Amber Flag	
Use of reagents in excess	Red Flag	

		Tick
Facile recovery of catalyst/enzyme	Green Flag	
catalyst/enzyme not recovered	Amber Flag	x

Critical elements

Supply remaining	Flag colour	Note element
5-50 years	Red Flag	x
50-500 years	Amber Flag	
+500 years	Green Flag	

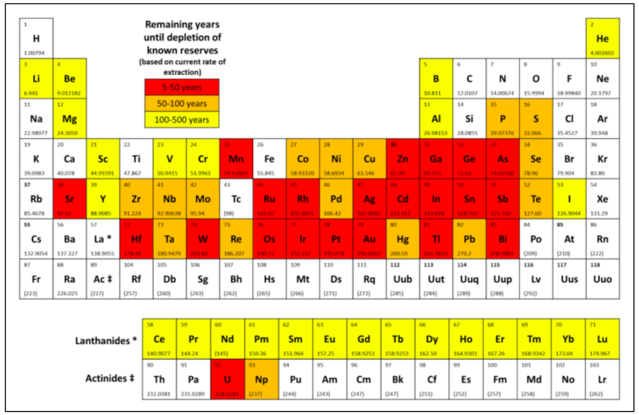

Energy (First Pass)

		Tick
Reaction run between 0 to 70°C	Green Flag	
Reaction run between -20 to 0 or 70 to 140°C	Amber Flag	x
Reaction run below -20 or above 140°C	Red Flag	

		Tick
Reaction run at reflux	Red Flag	x
Reaction run 5°C or more below the solvent boiling point	Green Flag	

Batch/flow

		Tick
Flow	Green Flag	
Batch	Amber Flag	x

Work Up

		List
quenching<br>filtration<br>centrifugation<br>crystallisation<br>Low temperature distillation/evaporation/<br>sublimation (< 140 °C at atmospheric	Green Flag	
solvent exchange, quenching into aqueous solvent	Amber Flag	

solvent exchange, quenching into aqueous solvent	Amber Flag	
chromatography/ion exchange<br>high temperature<br>multiple recrystallisation	Red Flag	x

Health & safety

	Red Flag	Amber Flag	Green Flag	List substances and H-codes	List substances and H-codes	List substances and H-codes
						**Pd/C** not classified as hazardous substance or mixture.
**Highly explosive**	H200, H201, H202, H203	H205, H220, H224	If no red or amber flagged H codes present then green flag			**1-hexanol**<br>H226 Flammable liquid and vapor
**Explosive thermal runaway**	H230, H240, H250	H241				**lithium tert-butoxide**<br>H251 Self-heating; may catch fire
**Toxic**	H300, H310, H330	H301, H311, H331,				**1-hexanol**<br>H302 + H312 Harmful if swallowed or in contact with skin.<br>H319 Causes serious eye irritation.<br>**lithium tert-butoxide**<br>H302 Harmful if swallowed<br>H314 Causes severe skin burns and eye damage
**Long Term toxicity**	H340, H350, H360, H370, H372	H341, H351, H361, H371, H373			**toluene**<br>H361 Suspected of damaging fertility or the unborn child.<br>H373 May cause damage to organs (Central nervous system) through prolonged or repeated exposure if inhaled	
**Environmental implications**	H400, H410, H411, H420	H401, H412		**phenol**<br>H411 Toxic to aquatic life with long lasting effects.	**toluene**<br>H401 Toxic to aquatic life.<br>H412 Harmful to aquatic life with long lasting effects	

Use of chemicals of environmental concern		List substances of very high concern
Chemical identified as Substances of Very High Concern by ChemSec which are utilised	Red Flag	

Number of red flags	Number of amber flags	Number of green flags
4	5	2

Table S28. *Angew. Chem. Int. Ed.* **2021**, *60*, 4043

Solvents (First Pass)

		List solvents below
**Preferred solvents**	**water,** EtOH, nBuOH, AcOipr, AcOnBu, PhOMe, MeOH, tBuOH, BnOH, ethylene glycol, acetone, MEK, MIBK, **AcOEt,** sulfolane	AcOEt
**Problematic solvents:** (acceptable only if substitution does not offer advantages)	DMSO, cyclohexanone, DMPU, AcOH, Ac2O, Acetonitrile, AcOMe, THF, heptane, Me-cyclohexane, toluene, xylene, MTBE, **cyclohexane**, chlorobenzene, formic acid, pyridine, Me-THF	toluene
**Hazardous solvents**: These solvents have significant health and/or safety concerns.	dioxane, pentane, TEA, diisopropyl ether, DME, DCM, DMF, DMA, NMP, methoxyethanol, hexane	
**Highly hazardous solvents:** The solvents which are agreed not to be used, even in screening	Et_2O, Benzene, CCl_4, chloroform, DCE, nitromethane, CS_2, HMPA	

Experimental:
'An oven-dried microwave reacting tube (10.0 mL) was charged with a magnetic stirbar, Pd/C (10 wt%, 15.0 mg, 7 mol% based on Pd contents, vacuum drying under reduced pressure for six hours) and lithium tert -butoxide (1.6 mg, 0.02 mmol). Then, toluene (1.0 mL), phenol (0.2 mmol) and alcohol (0.4 mmol) were added. The tube was sealed with rubber plug and evacuated by three freeze-pump-thaw cycles and backfilled with ultra-purified argon. Replace the rubber plug with an aluminum cover having a teflon pad. The tube was placed in a preheated oil bath at 160 °C and the mixture was stirred vigorously for 24 h. The reaction mixture was cooled to room temperature and filtered through the pad of silica gel. The filtrate was concentrated and the resulting residue was purified via the column chromatography'

Catalyst/enzyme (First Pass)

		Tick
Catalyst or enzyme used, **or** reaction takes place	Green Flag	x
Use of stoichiometric quantities of reagents	Amber Flag	
Use of reagents in excess	Red Flag	

		Tick
Facile recovery of catalyst/enzyme	Green Flag	
catalyst/enzyme not recovered	Amber Flag	x

Critical elements

Supply remaining	Flag colour	Note element
5-50 years	Red Flag	
50-500 years	Amber Flag	x
+500 years	Green Flag	

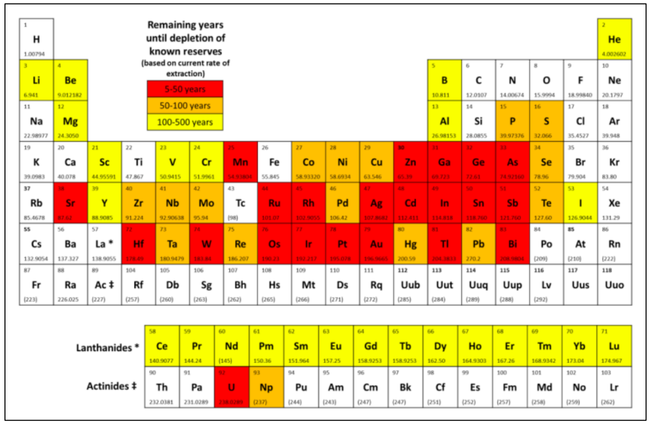

Energy (First Pass)

		Tick
Reaction run between 0 to 70°C	Green Flag	
Reaction run between -20 to 0 or 70 to 140°C	Amber Flag	
Reaction run below -20 or above 140°C	Red Flag	x

		Tick
Reaction run at reflux	Red Flag	x
Reaction run 5°C or more below the solvent boiling point	Green Flag	

Batch/flow

		Tick
Flow	Green Flag	
Batch	Amber Flag	x

Work Up

		List
quenching<br>filtration<br>centrifugation<br>crystallisation<br>Low temperature distillation/evaporation/<br>sublimation (< 140 °C at atmospheric	Green Flag	

chromatography/ion exchange<br>high temperature<br>multiple recrystallisation	Red Flag	x

Health & safety

	Red Flag	Amber Flag	Green Flag	List substances and H-codes	List substances and H-codes	List substances and H-codes
**Highly explosive**	H200, H201, H202, H203	H205, H220, H224	If no red or amber flagged H codes present then green flag			
**Explosive thermal runaway**	H230, H240, H250	H241				
**Toxic**	H300, H310, H330	H301, H311, H331,				**3,5-dimethoxyphenol**<br>H315 Causes skin irritation<br>H319 Causes serious eye irritation<br>H335 May cause respiratory irritation
**Long Term toxicity**	H340, H350, H360, H370, H372	H341, H351, H361, H371, H373		**1,2-dichloroethane**<br>H350 May cause cancer	**dichloromethane**<br>H351 Suspected of causing cancer<br>**2-pentanone**<br>H351 Suspected of causing cancer if inhaled.	
**Environmental implications**	H400, H410, H411, H420	H401, H412				

Use of chemicals of environmental concern		List substances of very high concern
Chemical identified as Substances of Very High Concern by ChemSec which are utilised	Red Flag	

Number of red flags	Number of amber flags	Number of green flags
6	5	2

Table S29. *J. Am. Chem. Soc.* **2021**, *143*, 33, 13428-13440

Solvents (First Pass)

		List solvents below
**Preferred solvents**	**water,** EtOH, nBuOH, AcOipr, AcOnBu, PhOMe, MeOH, tBuOH, BnOH, ethylene glycol, acetone, MEK, MIBK, **AcOEt,** sulfolane	
**Problematic solvents:** (acceptable only if substitution does not offer advantages)	DMSO, cyclohexanone, DMPU, AcOH, Ac2O, Acetonitrile, AcOMe, THF, heptane, Me-cyclohexane, toluene, xylene, MTBE, **cyclohexane**, chlorobenzene, formic acid, pyridine, Me-THF	
**Hazardous solvents:** These solvents have significant health and/or safety concerns.	dioxane, pentane, TEA, diisopropyl ether, DME, DCM, DMF, DMA, NMP, methoxyethanol, hexane	DCM
**Highly hazardous solvents:** The solvents which are agreed not to be used, even in screening	Et_2O, Benzene, CCl_4, chloroform, DCE, nitromethane, CS_2, HMPA	DCE

Experimental:
'In a glove box, complex 1 (18 mg, 3 mol %) was dissolved in 1,2-dichloroethane (0.5 mL) in a 25 mL Schlenk tube equipped with a Teflon stopcock and a magnetic stirring bar. 3-Methoxyphenol (124 mg, 1.0 mmol), 1-hexanal (100 mg, 1.0 mmol), 2-propanol (90 mg, 1.5 mmol) and 1,2-dichloroethane (0.5 mL) were added to the reaction tube. After the tube was sealed, it was brought out of the glove box and was stirred in an oil bath set at 120 °C for 12 h. The reaction tube was taken out of the oil bath and was cooled to room temperature. The product was isolated by a simple column chromatography on silica gel (40-63 μm particle size, hexanes/EtOAc = 80:1 to 10:1).'

Catalyst/enzyme (First Pass)

		Tick
Catalyst or enzyme used, **or** reaction takes place	Green Flag	x
Use of stoichiometric quantities of reagents	Amber Flag	
Use of reagents in excess	Red Flag	

		Tick
Facile recovery of catalyst/enzyme	Green Flag	
catalyst/enzyme not recovered	Amber Flag	x

Critical elements

Supply remaining	Flag colour	Note element
5-50 years	Red Flag	x
50-500 years	Amber Flag	
+500 years	Green Flag	

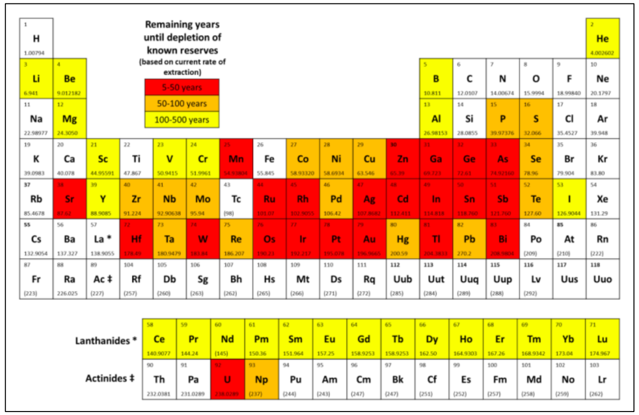

Energy (First Pass)

		Tick
Reaction run between 0 to 70°C	Green Flag	
Reaction run between -20 to 0 or 70 to 140°C	Amber Flag	x
Reaction run below -20 or above 140°C	Red Flag	

		Tick
Reaction run at reflux	Red Flag	x
Reaction run 5°C or more below the solvent boiling point	Green Flag	

Batch/flow

		Tick
Flow	Green Flag	
Batch	Amber Flag	x

Work Up

		List
quenching<br>filtration<br>centrifugation<br>crystallisation<br>Low temperature distillation/evaporation/<br>sublimation (< 140 ^{o}C at atmospheric	Green Flag	
solvent exchange, quenching into aqueous solvent	Amber Flag	

chromatography/ion exchange<br>high temperature<br>multiple recrystallisation	Red Flag	x

Health & safety

	Red Flag	Amber Flag	Green Flag	List substances and H-codes	List substances and H-codes	List substances and H-codes
Highly explosive	H200, H201, H202, H203	H205, H220, H224	If no red or amber flagged H codes present then green flag			**propanol**<br>H225 Highly flammable liquid and vapor
Explosive thermal runaway	H230, H240, H250	H241				
Toxic	H300, H310, H330	H301, H311, H331,			**3-methoxyphenol**<br>H311 Toxic in contact with skin.	**propanol**<br>H319 Causes serious eye irritation.<br>H336 May cause drowsiness or dizziness.
Long Term toxicity	H340, H350, H360, H370, H372	H341, H351, H361, H371, H373		**1,2-dichloroethane**<br>H350 May cause cancer	**dichloromethane**<br>H351 Suspected of causing cancer	
Environmental implications	H400, H410, H411, H420	H401, H412			**1-hexanal**<br>H401 Toxic to aquatic life.	

Use of chemicals of environmental concern		List substances of very high concern
Chemical identified as Substances of Very High Concern by ChemSec which are utilised	Red Flag	

Number of red flags	Number of amber flags	Number of green flags
6	6	2

Table S30. This work in batch recovering catalyst and alcohol

Solvents (First Pass)

		List solvents below
**Preferred solvents**	**water,** EtOH, nBuOH, AcOipr, AcOnBu, PhOMe, MeOH, tBuOH, BnOH, ethylene glycol, acetone, MEK, MIBK, **AcOEt,** sulfolane	EtOAc, SOLVENT FREE
**Problematic solvents:** (acceptable only if substitution does not offer advantages)	DMSO, cyclohexanone, DMPU, AcOH, Ac2O, Acetonitrile, AcOMe, THF, heptane, Me-cyclohexane, toluene, xylene, MTBE, **cyclohexane,** chlorobenzene, formic acid, pyridine, Me-THF	
**Hazardous solvents**: These solvents have significant health and/or safety concerns.	dioxane, pentane, TEA, diisopropyl ether, DME, DCM, DMF, DMA, NMP, methoxyethanol, hexane	
**Highly hazardous solvents:** The solvents which are agreed not to be used, even in screening	Et_2O, Benzene, CCl_4, chloroform, DCE, nitromethane, CS_2, HMPA	

Experimental:
'In a 12 mL screw-cap vial equipped with a magnetic stir bar, 8.5 mg of Pd/C (2 mol%), 5.9 mg of Sc(OTf)3 (3 mol%) and 49.6 mg of 4-methoxyphenol (0.4 mmol) are added. Then, 1 mL of 1-hexanol (20 eq) is added, and the mixture is kept stirring at 160°C for 20 h. Once the reaction is finished, the mixture is cooled to room temperature; Pd/C was filtered off from the reaction mixture using a Hirsh funnel and washed with EtOAc (5 mL) The excess of alcohol is separated from the filtrate by distillation and the residue is purified by chromatographic column using a variable ratio eluent mixture of ETP and EtOAc. The purified product is obtained as yellow oil (75% isolated yield, 62 mg). Pd/C is further washed with 5 mL water. The recovered catalyst was dried at 130°C under vacuum for 3 h and reused without significant change in weight. '

Catalyst/enzyme (First Pass)

		Tick
Catalyst or enzyme used, **or** reaction takes place	Green Flag	x
Use of stoichiometric quantities of reagents	Amber Flag	
Use of reagents in excess	Red Flag	

		Tick
Facile recovery of catalyst/enzyme	Green Flag	x
catalyst/enzyme not recovered	Amber Flag	

Critical elements

Supply remaining	Flag colour	Note element
5-50 years	Red Flag	
50-500 years	Amber Flag	x
+500 years	Green Flag	

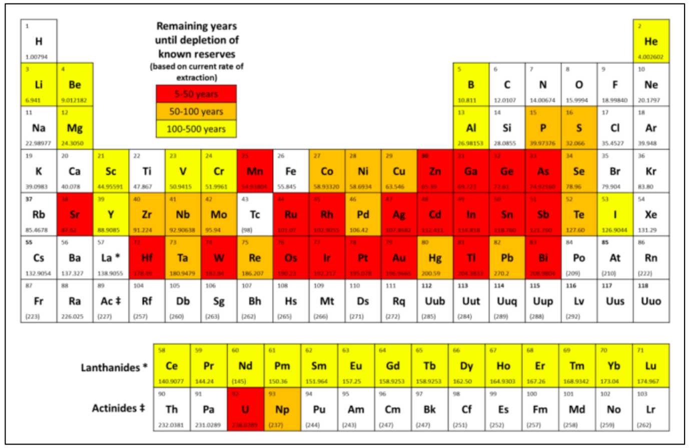

Energy (First Pass)

		Tick
Reaction run between 0 to 70°C	Green Flag	
Reaction run between -20 to 0 or 70 to 140°C	Amber Flag	
Reaction run below -20 or above 140°C	Red Flag	x

		Tick
Reaction run at reflux	Red Flag	
Reaction run 5°C or more below the solvent boiling point	Green Flag	x

Batch/flow

		Tick
Flow	Green Flag	
Batch	Amber Flag	x

Work Up

		List
quenching<br>filtration<br>centrifugation	Green Flag	

crystallisation<br>Low temperature distillation/evaporation/<br>sublimation (< 140 °C at atmospheric pressure)	Green Flag	
solvent exchange, quenching into aqueous solvent	Amber Flag	
chromatography/ion exchange<br>high temperature<br>multiple recrystallisation	Red Flag	x

Health & safety

	Red Flag	Amber Flag	Green Flag	List substances and H-codes	List substances and H-codes	List substances and H-codes
						**Sc(OTf)3** and **Pd/C** not classified as hazardous substances or mixtures.
**Highly explosive**	H200, H201, H202, H203	H205, H220, H224	If no red or amber flagged H codes present then green flag			**1-hexanol**<br>H226 Flammable liquid and vapor
**Explosive thermal runaway**	H230, H240, H250	H241				
**Toxic**	H300, H310, H330	H301, H311, H331,				**1-hexanol**<br>H302 + H312 Harmful if swallowed or in contact with skin.<br>H319 Causes serious eye irritation.<br>**ethyl acetate**<br>H319 Causes serious eye irritation.<br>H336 May cause drowsiness or dizziness
**Long Term toxicity**	H340, H350, H360, H370, H372	H341, H351, H361, H371, H373				
**Environmental implications**	H400, H410, H411, H420	H401, H412			**4-methoxyphenol**<br>H401 Toxic to aquatic life.<br>H412 Harmful to aquatic life with long lasting effects.	

Use of chemicals of environmental concern		List substances of very high concern
Chemical identified as Substances of Very High Concern by ChemSec which are utilised	Red Flag	

Number of red flags	Number of amber flags	Number of green flags
2	3	9

Table S31. This work in flow

Solvents (First Pass)

		List solvents below
**Preferred solvents**	**water,** EtOH, nBuOH, AcOipr, AcOnBu, PhOMe, MeOH, tBuOH, BnOH, ethylene glycol, acetone, MEK, MIBK, **AcOEt,** sulfolane	EtOAc + NO SOLVENT
**Problematic solvents:** (acceptable only if substitution does not offer advantages)	DMSO, cyclohexanone, DMPU, AcOH, Ac2O, Acetonitrile, AcOMe, THF, heptane, Me-cyclohexane, toluene, xylene, MTBE, **cyclohexane**, chlorobenzene, formic acid, pyridine, Me-THF	
**Hazardous solvents**: These solvents have significant health and/or safety concerns.	dioxane, pentane, TEA, diisopropyl ether, DME, DCM, DMF, DMA, NMP, methoxyethanol, hexane	
**Highly hazardous solvents:** The solvents which are agreed not to be used, even in screening	Et_2O, Benzene, CCl_4, chloroform, DCE, nitromethane, CS_2, HMPA	

Experimental:
'The flow streams driven by the HPLC pump containing the solution of $Sc(OTf)_3$ (3 mol%, 118 mg), 3,5-dimethoxyphenol (8 mmol, 1.232 g) and 1-hexanol (20 equiv, 20 mL) was directed through the tube-in-tube reactor packed with Pd/C (10% w/w, 344 mg, 3.8 mmol of Pd) dispersed in quartz (99 ww%) placed in a reactor installed in an aluminium brick at 160 °C at a pressure of 5 Bar of compressed air with 0.5 mL/min flow rate. The reaction mixture was continuously pumped with a residence time inside the reactor of 348 min. The reaction mixture at the outlet of the reactor was collected into a flask, 1-hexanol was removed via distillation under vacuum and the crude mixture was purified by column chromatography.'

Catalyst/enzyme (First Pass)

		Tick
Catalyst or enzyme used, **or** reaction takes place	Green Flag	x
Use of stoichiometric quantities of reagents	Amber Flag	
Use of reagents in excess	Red Flag	

		Tick
Facile recovery of catalyst/enzyme	Green Flag	x
catalyst/enzyme not recovered	Amber Flag	

Critical elements

Supply remaining	Flag colour	Note element
5-50 years	Red Flag	
50-500 years	Amber Flag	x
+500 years	Green Flag	

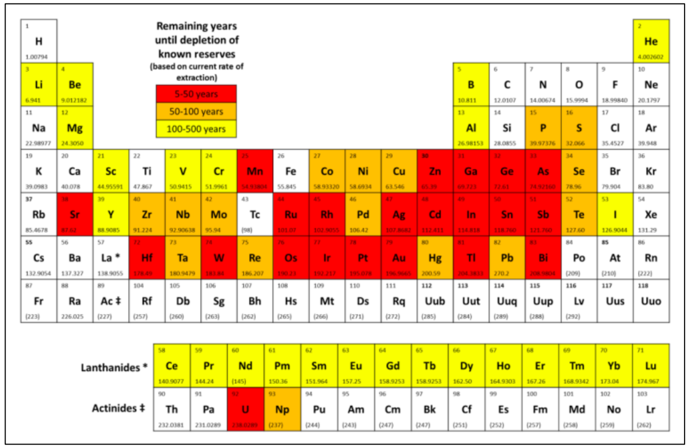

Energy (First Pass)

		Tick
Reaction run between 0 to 70°C	Green Flag	
Reaction run between -20 to 0 or 70 to 140°C	Amber Flag	
Reaction run below -20 or above 140°C	Red Flag	x

		Tick
Reaction run at reflux	Red Flag	
Reaction run 5°C or more below the solvent boiling point	Green Flag	x

Batch/flow

		Tick
Flow	Green Flag	x
Batch	Amber Flag	

Work Up

		List
quenching<br>filtration<br>centrifugation	Green Flag	

crystallisation<br>Low temperature distillation/evaporation/<br>sublimation (< 140 ^{o}C at atmospheric pressure)	Green Flag	
solvent exchange, quenching into aqueous solvent	Amber Flag	
chromatography/ion exchange<br>high temperature<br>multiple recrystallisation	Red Flag	x

Health & safety

	Red Flag	Amber Flag	Green Flag	List substances and H-codes	List substances and H-codes	List substances and H-codes
						**Sc(OTf)3** and **Pd/C** not classified as hazardous substances or mixtures.
**Highly explosive**	H200, H201, H202, H203	H205, H220, H224	If no red or amber flagged H codes present then green flag			**1-hexanol**<br>H226 Flammable liquid and vapor
**Explosive thermal runaway**	H230, H240, H250	H241				
**Toxic**	H300, H310, H330	H301, H311, H331,				**1-hexanol**<br>H302 + H312 Harmful if swallowed or in contact with skin.<br>H319 Causes serious eye irritation.<br>**3,5-dimethoxyphenol**<br>H315 Causes skin irritation<br>H319 Causes serious eye irritation<br>H335 May cause respiratory irritation
**Long Term toxicity**	H340, H350, H360, H370, H372	H341, H351, H361, H371, H373				
**Environmental implications**	H400, H410, H411, H420	H401, H412				

Use of chemicals of environmental concern		List substances of very high concern
Chemical identified as Substances of Very High Concern by ChemSec which are utilised	Red Flag	

Number of red flags	Number of amber flags	Number of green flags
2	1	10
